# Effective, Robust Design of Community Mitigation for Pandemic Influenza: A Systematic Examination of Proposed US Guidance

**DOI:** 10.1371/journal.pone.0002606

**Published:** 2008-07-02

**Authors:** Victoria J. Davey, Robert J. Glass, H. Jason Min, Walter E. Beyeler, Laura M. Glass

**Affiliations:** 1 Uniformed Services University of the Health Sciences, Bethesda, Maryland, United States of America; 2 Office of Public Health and Environmental Hazards, Veterans Health Administration, Department of Veterans Affairs, Washington D. C., United States of America; 3 National Infrastructure Simulation and Analysis Center, Sandia National Laboratories, Albuquerque, New Mexico, United States of America; 4 Albuquerque Public Schools, Albuquerque, New Mexico, United States of America; Tel Aviv University, Israel

## Abstract

**Background:**

The US government proposes pandemic influenza mitigation guidance that includes isolation and antiviral treatment of ill persons, voluntary household member quarantine and antiviral prophylaxis, social distancing of individuals, school closure, reduction of contacts at work, and prioritized vaccination. Is this the best strategy combination? Is choice of this strategy robust to pandemic uncertainties? What are critical enablers of community resilience?

**Methods and Findings:**

We systematically simulate a broad range of pandemic scenarios and mitigation strategies using a networked, agent-based model of a community of explicit, multiply-overlapping social contact networks. We evaluate illness and societal burden for alterations in social networks, illness parameters, or intervention implementation. For a 1918-like pandemic, the best strategy minimizes illness to <1% of the population and combines network-based (e.g. school closure, social distancing of all with adults' contacts at work reduced), and case-based measures (e.g. antiviral treatment of the ill and prophylaxis of household members). We find choice of this best strategy robust to removal of enhanced transmission by the young, additional complexity in contact networks, and altered influenza natural history including extended viral shedding. Administration of age-group or randomly targeted 50% effective pre-pandemic vaccine with 7% population coverage (current US H5N1 vaccine stockpile) had minimal effect on outcomes. In order, mitigation success depends on rapid strategy implementation, high compliance, regional mitigation, and rigorous rescinding criteria; these are the critical enablers for community resilience.

**Conclusions:**

Systematic evaluation of feasible, recommended pandemic influenza interventions generally confirms the US community mitigation guidance yields best strategy choices for pandemic planning that are robust to a wide range of uncertainty. The best strategy combines network- and case-based interventions; network-based interventions are paramount. Because strategies must be applied rapidly, regionally, and stringently for greatest benefit, preparation and public education is required for long-lasting, high community compliance during a pandemic.

## Introduction

### Background

Human influenza pandemics of unpredictable severity and consequence are considered inevitable. Studies using computational models have examined roles of border controls, internal travel restrictions, treatment and prophylaxis with antivirals, isolation of cases, quarantine of household members, school closure, and partially effective vaccine on limiting outbreak sizes [1]–[7]. Others have modeled a pandemic at large scale either from a source in SE Asia [8], [9], or within the US or Great Britain [10], [11] in order to describe effects of vaccination, school closures, and voluntary or imposed partial or total community member quarantine. Still others have focused at the community scale to consider community containment options [12]–[14]. The findings from these and other large and small scale simulation studies underpin the US government's *Interim Pre-pandemic Planning Guidance: Community Strategy for Pandemic Influenza Mitigation in the United States* [15].

Yet, these studies have explored the influence of mitigation strategies through a limited set of defining scenarios. Many questions are left unanswered: Which combinations of interventions (which we refer to as strategies) best controls illness, death, and societal disruption on a community scale? Is the choice of a best strategy robust to biologic, physical, and behavioral human and viral heterogeneity? What are the most important enabling components of effective community mitigation that yield a resilient community, able to surmount a pandemic's effects?

To address these questions, we systematically and broadly explored the effectiveness of community mitigation strategies with a networked, agent-based computational model. Our model, Loki-Infect, simulates transmission within the explicit social contact network of a stylized community. It is parsimonious, incorporating only the critical details that are required to answer our stated questions. We began with a base set of model parameters and ran simulations of a core matrix of 64 community mitigation strategies formed by combinations of 8 independent interventions that encompass the US government's planning guidance [15]. We examined a range of influenza severity from a seasonal influenza outbreak to twice that of the 1918 influenza pandemic and varied model structures and parameter assumptions through an extensive set of perturbations and extensions. From the results, we chose and present the combination of interventions that best limit illness, death, and societal disruption in the face of a 1918-like pandemic and show that this best choice is robust to the model uncertainties considered. Finally, by examining the dependence of strategy effectiveness on parameter variation we identify the critical enablers of community resilience.

## Methods

Loki-Infect is a networked agent-based computational model developed by the National Infrastructure Simulation and Analysis Center (NISAC) at Sandia National Laboratories. In this model, agents represent individuals of various age classes who are linked to each other within and among social groups (such as households, neighborhoods, school classes, clubs, businesses, etc.) to form an explicit contact network reflective of a multiply-overlapping, structured community. Behavioral rules for individuals, their interactions, and the performance of network links are specified to model the spread of influenza. Community mitigation strategies are implemented through modifications of these behavioral rules when a given strategy is imposed during a simulation. In context of pandemic influenza, Loki-Infect has been applied to evaluate social distancing strategies [13], design targeted social distancing [14], evaluate rescinding criteria for mitigation measures [12], and design community containment strategies [16].

A simulation begins by creating a community (in the current case composed of 10,000 individuals) and then seeding it with the infection of an initial group of randomly-chosen community adults. These adults become infectious and may infect others, depending on their contacts and the contacts' age-related susceptibility. Fifty percent of infected persons become ill, 80% of ill persons are “diagnosed” with the pandemic virus and go home where they decrease their interactions with others outside the household. If the sick person is a child, a well adult from the household stays home with the child. Two percent of those that are ill die, the remainder recover. If the simulation includes use of mitigation interventions, these begin immediately at a predefined number of incident cases (10, 30, or 100), are carried out by the community with a specified level of compliance (60% or 90%), and end at a defined threshold of number of new cases in a 7 day period (0 or 3). This immediate implementation includes distribution and administration of antivirals, which is a best case situation. Subsequently, if the number of newly diagnosed individuals rises above the implementation threshold, mitigation strategies are reapplied with a second mitigation cycle. If required, additional cycles based on these beginning and rescinding thresholds are implemented until no infected individuals remain within the community and the simulation ends.

Complete model details are provided in the Supplemental Information **(** **Methods S1** ****). Included in **Methods S1** are specifications of the basic contact network, behavioral rules for the spread of influenza, alternative manifestations of influenza natural history, alternative networks used for infectious contacts, variation of viral infectivity, instigation and boundary condition alternatives, community mitigation interventions, and simulation study design. Links to Excel tables of results are included in **Methods S1**; full data and the model code are available on request from the authors.

We investigated 8 independent mitigation interventions defined in **Table 1**. Network-based interventions affect links and contacts between individuals throughout the entire network and are: School closure (S), Child and Teen social distancing (CTsd), and Adult and Senior social distancing (ASsd) (where adults continue to attend work, although at a 50% reduction in contacts at the workplace). Case-based interventions are applied directly to or around diagnosed individuals to limit transmission and are: household Quarantine (Q), antiviral Treatment (T), household antiviral Prophylaxis (P), and Extended contact Prophylaxis (PEx). Each intervention is implemented singly or in combinations as strategies to yield an 8 by 8 combinatory matrix (**Table 2**). Antiviral interventions T, P and PEx are nested, with P necessarily incorporating T, and PEx necessarily incorporating both T and P. For all, a compliance factor is applied that specifies the percentage of individuals that comply with interventions.

**Table 1:** Mitigation Interventions.

Intervention	Definition
**S**	Schools closed, all school contacts reduced by 90%, household contacts doubled. One adult from each household with a child (11 or younger) stays home from work.
**CTsd**	Social distancing of children and teenagers. All non-school and non-household contacts with or between children and teenagers reduced by 60% and 90%, household contacts doubled.
**ASsd**	Social distancing of adults and seniors. All non-household, non-work contacts within and between adults and seniors reduced by 60% and 90%, work contacts reduced by 50%; household contacts doubled.
**Q**	Household quarantine for 10 days once an individual in the household is diagnosed, all non-household contacts reduced by 60% or 90%, household contacts doubled.
**T**	Antiviral treatment. Individual given antiviral course with probability (60%, 90%) for 5 days immediately after diagnosis, reduces infectivity by 60% [8], [10]
**P**	Household member antiviral prophylaxis. Household members given an antiviral with probability (60%, 90%) for 10 days starting immediately after household reference case is diagnosed, reduces infection susceptibility by 30%, reduces probability of clinical illness by 65%, reduces infectivity by 60% [8], [10]
**PEx**	Extended contact prophylaxis. Household members, workplace contacts, school contacts, work contacts, and neighborhood/extended family contacts of a case are given antivirals with probability (60%, 90%) for 10 days starting immediately after reference case diagnosed; reduces infection susceptibility by 30%; reduces probability of clinical illness by 65%; reduces infectivity by 60%. (Note that school and workplace contact rates used for PEx are much less than the entire school or work groups.)

**Table 2:** Combinatory strategy matrix: Case-based as rows, network-based as columns.

	None	ASsd	CTsd	CTsd, ASsd	S	S, ASsd	S, CTsd	S, CTsd ASsd
**None**	None							
**T**								
**Q**								
**P**								
**Q,T**								
**Q,P**								
**PEx**								
**Q,PEx**								All

S = schools closed; CTsd = child/teenager social distancing; ASsd = adult/senior socials distancing; Q = household quarantine; T = antiviral treatment; P = antiviral prophylaxis of household members; PEx = extended antiviral prophylaxis. Case-based interventions are applied directly to or around diagnosed individuals to limit transmission (T, Q, P, PEx). Network-based interventions are applied to affect links and contacts between individuals throughout the entire network (ASsd, CTsd, S).

### Core Analysis

For our core analysis we used the base contact network from previous Loki-Infect studies [12]–[14], [16]. This base network models mathematically both a closed community with no external interactions as well as a fully open community that is in interaction with like communities implementing identical mitigation strategies and similarly seeded with infectious individuals. The fully open community may also be thought of as geographically contiguous with other identical communities to compose a larger city. Contacts among any of the groups outside the household could therefore originate from anywhere within the city. We refer to the use of this base contact network as ‘regional mitigation’.

We used a natural history of influenza for the core analysis that conforms closely to Ferguson et al. [8], [10]. Because of expected high morbidity associated with a pandemic strain, we add a mean 7 day recovery period after the symptomatic period to the Ferguson-like manifestation for those individuals who are diagnosed and withdraw to the home. During this recovery period, individuals continue to stay at home but are not infectious.

Compliance of 90%, a strategy implementation threshold of 10 diagnosed individuals and a strategy rescinding threshold of 7 days with no newly diagnosed individuals was used for the core analysis as a possible best case, reflecting a situation of excellent surveillance and community participation.

### Perturbations and Extensions

To evaluate the sensitivity of our core model parameters and identify critical enablers of resilience, we varied parameters and model assumptions within a range thought to bound realism: relaxed compliance (60%); delayed strategy implementation thresholds (day after 30 or 100 individuals are diagnosed within the community); relaxed rescinding of strategies (3 cases in 7 days). We also implemented local-only mitigation; alternative influenza natural history (Longini-like and Longini-like with an extended period of infectiousness); two alternative contact networks (similar transmission by all age classes or augmented with additional contact groups); and availability of pre-pandemic vaccine (uniform or age class targeted). Details of all these perturbations and extensions are given in **Methods S1**. Below we provide salient features.

To remove our core assumption of regional implementation of like mitigation strategies, we simulated a community embedded within a region doing nothing to abate the epidemic, referred to as ‘local-only mitigation’. In local-only mitigation, contact with the external regional population occurs exclusively through the work environment. This would represent a town where workers commute from elsewhere or a city where non-household contacts are from within the neighborhood, except at work, where all contacts are from outside.

As a first alternative manifestation of influenza natural history, we implemented that of Longini and colleagues [9], [11]. The critical differences between the Ferguson-like influenza natural history used in our core analysis and that of the Longini-like are the proportion of infected individuals who develop clinical illness (50% in our core analysis vs. 67% for Longini) and a somewhat extended period of infectiousness. As with the Ferguson-like manifestation, a recovery period with a mean of 7 days is added after the symptomatic period for those individuals who are diagnosed and withdraw to the home. As a second alternative manifestation of influenza natural history, we added an extended period of shedding that may accompany particularly novel strains (such as may occur with the H5N1 subtype affecting humans) [17], [18]. Consequently, the Longini-like manifestation's period of infectiousness was extended to the end of the recovery period. As a first alternative contact network, we made transmission capability similar for the young and adults. The enhanced relative infectivity and susceptibility for children and teenagers was removed and the number of contacts for adults within the workplace was increased to put them on par with children and teenagers in schools. While we believe these two characteristics are unlikely in combination, they represent one extreme that bounds uncertainty in the resulting network of infectious contacts. As a second alternative contact network, we augmented the network by placing all 0 to 5 year old children in preschool or play groups and adding child, teen, adult and senior social clubs, teen friend groups, and adult task groups to the basic contact network. Children and teenagers were also given additional random networks to reflect, for example, hallway passing within schools. Finally, the number of links within child classes was increased and the average frequency of contact per link in extended families or neighborhoods was reduced. This alternative network was guided by on a recent characterization of contact networks for children and teenagers [19].

As a final extension, we considered the availability of 50% effective pre-pandemic vaccine applied with 7% community coverage in 3 vaccination strategies: either randomly administered to community members, targeted to children and teens, or targeted to adults. This constitutes a level of availability and assumed efficacy of the proposed pre-pandemic vaccine stockpile in the US [20]. Administering pre-pandemic vaccine to adults reflects a proposed strategy of providing vaccine to protect critical workers [21].

### Simulations

For each combination of parameters and model assumptions that define an intervention combination for the core analysis or perturbations and extensions to examine sensitivity, we varied the viral infectivity about a base defined by an infection rate of 50%, representative of the 1958 pandemic [10]. We scale this base infectivity by factors (*I_F_*) of 0.75, 1.0, 1.25, 1.5, 2.0, 2.5 and 3.0 to yield lower and higher infection rates. An *I_F_* of 1.5 results in the current accepted infectivity for the 1918–1920 pandemic, equivalent to an *R_0_* of about 2.0 (*R_0_* is the number of secondary infections produced by one individual in a susceptible population; when *R_0_* is <1.0 an epidemic cannot continue to propagate [22], [23]). Epidemic severity is a combination of viral infectivity, which determines how many people are infected, and the case fatality rate, which determines how many people die [15]. While any case fatality rate can be applied to the simulation results, we present a common rate of 2% of those with clinical illness (clinical illness occurs in 50% of those infected) as was thought to be reflective of the 1918 pandemic [23].

To capture the stochastic variability that is inherent and expected due to the variability of social network structure, individual links and contacts, and those who are initially infected, we conducted 100 simulations for each combination of parameters. Across the entire simulation set (core, perturbations, extensions), nearly 2 million simulations were conducted in total. Only those simulations that created epidemics (defined as greater than 1 percent of the population infected) were used in analysis of outcomes.

## Results

We first briefly describe the core analysis, we then use the results to design a community mitigation strategy for a 1918-like pandemic, and finally we test the sensitivity of this design to perturbations of the parameters and assumptions of the model. We focus on the outcome measures of infection rates (to which deaths are directly related), the average number of days adults are at home (either sick, quarantined in the home, or tending children sick or dismissed from school) and the community antiviral coverage required for a particular strategy. Full results and their discussion can be found in the **Results and Appendices in** **Methods S1**. Other outcome measures available in **Methods S1** include the number of simulations that yield epidemics, cumulative illness rates, deaths, peak numbers of infected or symptomatic individuals, time to peak infected or symptomatic, epidemic duration, total time of epidemic effects, number of days strategies are imposed, number of strategy cycles needed, and number of infections resulting from external community contacts.

### Core Analysis

**Table 3** displays outcomes for unmitigated epidemics at infectivity factors (*I_F_*) from seasonal influenza-like (*I_F_* .75) to twice 1918-like (*I_F_* 3.0) and compares them to the US Pandemic Severity Index (PSI) [15]. Again, a case fatality rate of 2% of those with clinical illness (thought to be reflective of 1918) is used across all *I_F_*. For any *I_F_*, different case fatality rates and thus severities can be obtained by rescaling the number of deaths resulting from these simulations. As a further reminder, the core analysis reflects the basic contact network with Ferguson-like natural history of influenza, 90 percent compliance, rapid implementation (day after 10 cases diagnosed), restrictive rescinding (0 cases in 7 days) and regional mitigation.

**Table 3:** Pandemic outcomes for unmitigated epidemics by *I_F_* *.

	*I_F_*			
	0.75	1	1.5	3
**% Infected**	29	50	71	92
**% Symptomatic**	14	25	36	46
**No. of deaths**	28	50	71	92
**No. of adult days at home**	1	2	3	4
**Epidemic duration (days)**	104	61	42	27
**PSI equivalent** †	<1	1 to 2	4 to 5	not on PSI scale

* *I_F_* is scaled disease infectivity (uses transmissibility as a measure of pandemic severity).

† PSI is the pandemic severity index from [15], a scale of predicted pandemic impact based on early estimation of case fatality rate.

Case fatality rate here is .02 across all *I_F_*.

Results are for simulations (of 100 done for each *I_F_*) that produced epidemics. An epidemic is defined as when >1.0% of population is infected.

**Figure 1:**
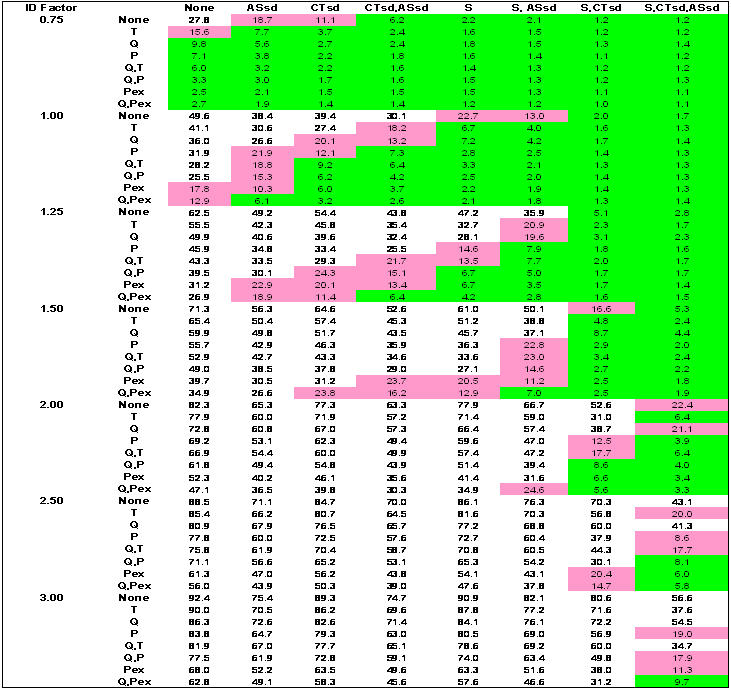
Percentage of population infected. S = schools closed; CTsd = child/teenager social distancing; ASsd = adult/senior socials distancing; Q = household quarantine; T = antiviral treatment; P = antiviral prophylaxis of household members; PEx = extended antiviral prophylaxis. For Ferguson-like disease manifestation and implementation threshold when 10 cases are diagnosed. Case-based interventions are applied directly to or around diagnosed individuals to limit transmission and are: Q, T, P, and PEx. Network-based interventions are applied to affect links and contacts between individuals throughout the entire network and are: S, CTsd, ASsd (where adults continue to attend work, although at a 50% reduction in contacts at the workplace). Case-based interventions vertical; network-based interventions horizontal. Green shading denotes infection rates ≤10 percent of population. Pink shading denotes infection rates between 10 and 25 percent.

**Figures 1** **,** **2** **, and** **3** show results for combinatory intervention matrices over the range of epidemic infectivity (*I_F_*) for the outcomes of infection rates, adult days at home, and antiviral courses used, respectively. Network-based and case-based interventions applied alone or in combination as strategies yield banded green zones where infection rates are 10 percent or less, and pink zones where infection rates are 10 to 25 percent. The less-than-10-percent green zone is concentrated where more interventions are imposed (the lower right corners of each *I_F_* region). An infection rate of 10 percent (green zone) corresponds to a symptomatic illness rate of 5 percent and a diagnosed rate of 4 percent. An infection rate of 25 percent (pink zone) corresponds to a symptomatic illness rate of 12.5 percent and a diagnosis rate of 10 percent.

**Figure 2 pone-0002606-g002:**
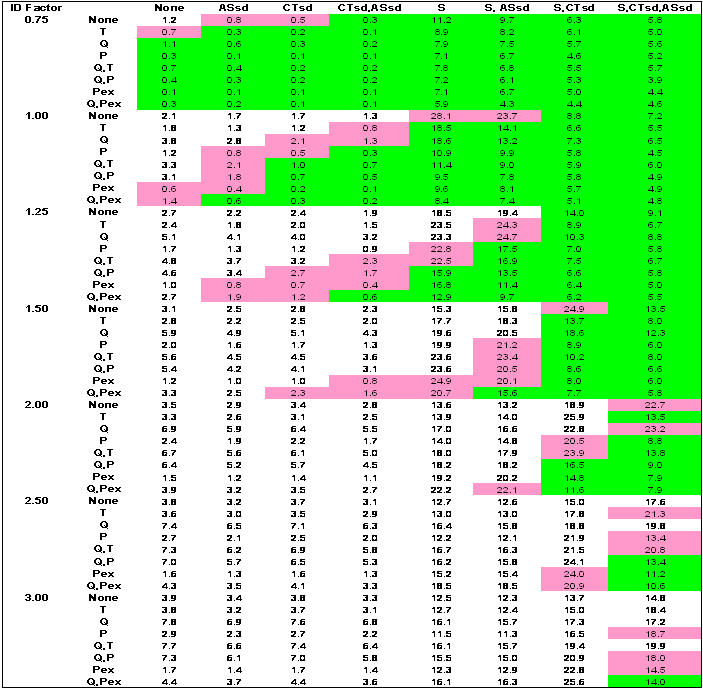
Average adult days at home. S = schools closed; CTsd = child/teenager social distancing; ASsd = adult/senior socials distancing; Q = household quarantine; T = antiviral treatment; P = antiviral prophylaxis of household members; PEx = extended antiviral prophylaxis. For Ferguson-like disease manifestation and implementation threshold when 10 cases are diagnosed. Case-based interventions are applied directly to or around diagnosed individuals to limit transmission and are: Q, T, P, and PEx. Network-based interventions are applied to affect links and contacts between individuals throughout the entire network and are: S, CTsd, ASsd (where adults continue to attend work, although at a 50% reduction in contacts at the workplace). Case-based interventions vertical; network-based interventions horizontal. Green shading denotes infection rates ≤10 percent of population. Pink shading denotes infection rates between 10 and 25 percent.

**Figure 3:**
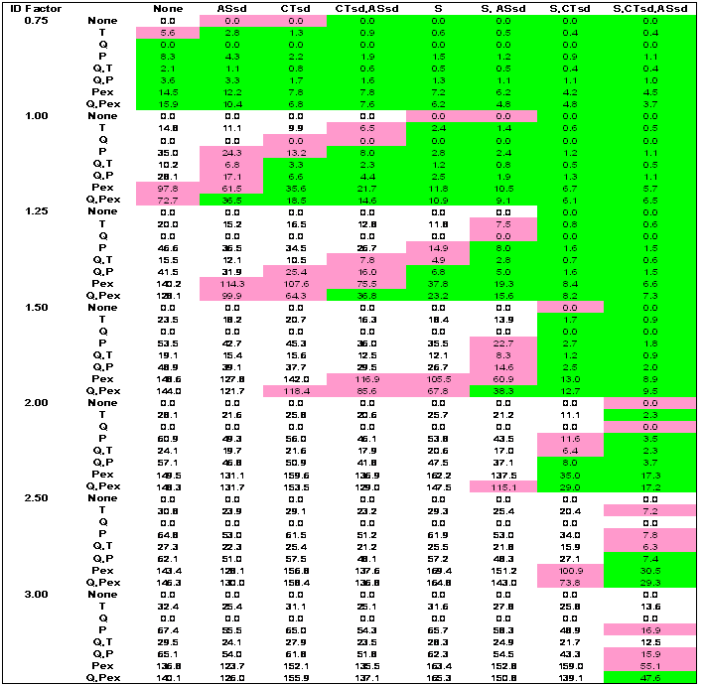
Population antiviral coverage. S = schools closed; CTsd = child/teenager social distancing; ASsd = adult/senior socials distancing; Q = household quarantine; T = antiviral treatment; P = antiviral prophylaxis of household members; PEx = extended antiviral prophylaxis. For Ferguson-like disease manifestation and implementation threshold when 10 cases are diagnosed. Case-based interventions are applied directly to or around diagnosed individuals to limit transmission and are: Q, T, P, and PEx. Network-based interventions are applied to affect links and contacts between individuals throughout the entire network and are: S, CTsd, ASsd (where adults continue to attend work, although at a 50% reduction in contacts at the workplace). Case-based interventions vertical; network-based interventions horizontal. Green shading denotes infection rates ≤10 percent of population. Pink shading denotes infection rates between 10 and 25 percent.

Combinations of network-based interventions can effectively reduce infection rates to less than 10% of the population up to an *I_F_* of 1.5 (**Figure 1**). Above an *I_F_* of 1.5, combinations of network- and case-based interventions are required. At the lowest *I_F_* (0.75), the efficacy of network-based interventions applied alone increases from ASsd, CTsd, CTsd+ASsd, S, S+ASsd, to S+CTsd or S+CTsd+ASsd. At an *ID_F_* of 1.5, this order changes, increasing from CTsd, S, ASsd, CTsd+ASsd, S+ASsd, S+CTsd, to S+CTsd+ASsd. As *I_F_* rises, combinations that include S or ASsd increase in efficacy over those with CTsd alone (because CTsd removes non-school contacts but leaves within-school contacts of children and teenagers). Efficacy of ASsd improves because with increasing *I_F_*, the branching factor for adults in the unmitigated epidemic, which is akin to an age class specific *R_0_*, is pushed above 1.0. Thus, adult contacts become responsible for a larger share of transmission. Since there are more adults in the community, restricting them contributes more to overall epidemic control.

For case-based interventions applied without any network-based interventions, the efficacy increases from T through Q, P, Q+T, Q+P, and PEx, to Q+PEx (**Figure 1**). This order does not change as *I_F_* increases. For an epidemic above an *I_F_* of 0.75, case-based strategies alone cannot contain the infection rate below 10 percent. Note that the model implements use of case-based measures immediately on the 10 case trigger, which would be an unrealistic situation in any community, given difficulties in antiviral distribution.

Containing infection rates to less than 10 percent significantly reduced the total overall burden to the community as measured by the number of days that adults are at home (**Figure 2**). Implementing S+CTsd+ASsd always decreases this number over S+CTsd alone (e.g. 14 vs. 25 days, respectively, for *I_F_* 1.5 where no case based interventions are applied). Implementing S is the major component of the adult days at home measure because in the model, when a child (11 or younger) is diagnosed with influenza or when schools are closed, one household adult stays home to care for the child, and closing the schools requires approximately 22% of adults to be at home minding children. Assuming all these adult days at home are a loss to work productivity is a worst-case assessment. It is likely that some child-minding adults would maintain reasonable work productivity during school closures, because they usually work at home, or through telecommuting, time shifting, or job sharing. Additionally, teenagers present within the household could care for children and thus release the adult babysitter to attend work.

For all strategies that result in infection rates of 10 percent or less (the green zone), no more than 48% population coverage with antivirals is required, and this high value only occurs at an *I_F_* of 3 where all interventions are applied (**Figure 3**). If PEx is excluded, a maximum of only 8 percent antiviral coverage is required and for most of the strategies where infection rates are 10 percent or less, required antiviral coverage is far less. Applying PEx alone as a strategy results in less than 10 percent infected only at an *I_F_* of 0.75. At higher *I_F_*, applying PEx necessitates antiviral coverage of as much as 150 percent of the population, where each individual receives an antiviral course an average of 1.5 times over the course of the epidemic. This greater-than-100-percent coverage is also very ineffective in limiting infection rates to less than 10 or even 25 percent of the population.

### Designing Community Mitigation for 1918-like (PSI 4 to 5) Pandemic

**Figure 4:**
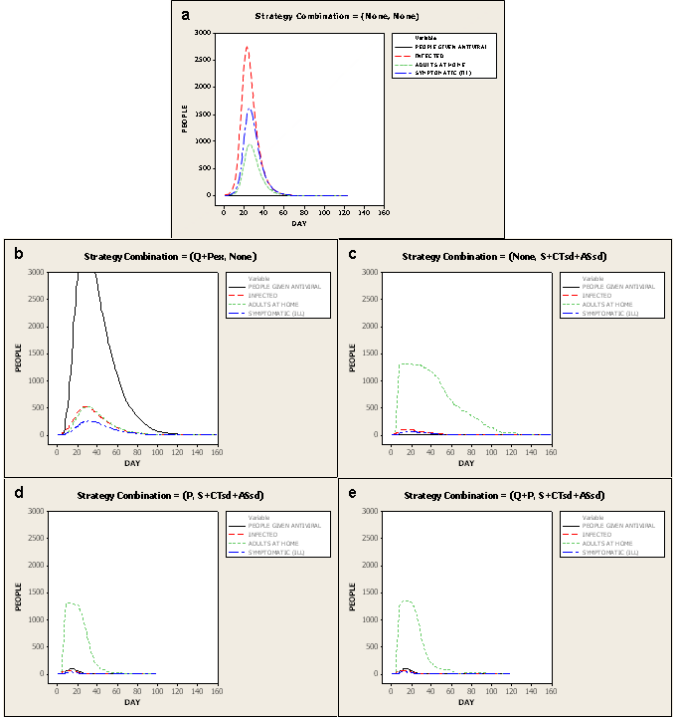
Epidemic effects without and with various mitigation strategies. Plots of numbers of individuals infected, symptomatic, given antivirals, and adult days at home by day of pandemic without mitigation strategies and with mitigation strategies applied for an *I_F_* 1.5 (1918-like; Pandemic Severity Index [PSI] 4–5 pandemic); with Ferguson-like disease manifestation and strategies implemented at 90% compliance with regional mitigation. Plots are averages of all 100 simulations done. Comparisons are to the plot with no mitigation strategies applied. a—no mitigation strategies applied. Note the early peak of 1500 symptomatic cases at day 28 and that approximately 900 adults (9% of the population) are at home from illness at peak. Epidemic effects end by day 60. b—all case-based interventions applied (Q+PEx). Note the significant requirements for antiviral drugs (>30% of population receives antivirals at peak). Symptomatic cases are contained to <250 at peak and adult days at home peak at 500 from illness or home quarantine. The epidemic effects last for 160 days. c—all network-based interventions applied (S+CTsd+ASsd). Note the significant, sustained increase in adult days at home because of the school closings and childcare required, peaking at approximately 1400 adults home/day and tapering off slowly. However, symptomatic cases are contained to <200 at peak. The epidemic effects last approximately 120 days. d—the best strategy we found in these simulations (P+S+CTsd+ASsd). Ill persons are treated with antivirals, household members of ill persons receive antiviral prophylaxis, schools are closed and children's and teenagers' contacts are reduced by 90%, adults' and seniors' non-work contacts are decreased by 90% and workplace contacts by 50%. Note the similar peak of adult days at home (at around 1400) as when only network-based strategies are applied, but with rapid fall-off, with nearly no adult days at home required after approximately day 50. Symptomatic cases are minimized to <200 at peak. Epidemic effects end around day 100. e—the best strategy (P+S+CTsd+ASsd) with Q added. Note that the addition of Q does not change the number of symptomatic cases, but does extend required adult days at home and lengthens the epidemic effects to approximately 120 days.

It is reasonable to plan to mitigate a pandemic like one the world has experienced. For an unmitigated 1918-like pandemic (PSI 4 to 5) with an *I_F_* of 1.5, 71 percent of the population is infected, 36 percent of the infected are symptomatic, and 2 percent of the symptomatic die. **Figure 4** displays average daily numbers of infections, symptomatic cases, individuals given antivirals, and adult days at home plotted over time for the full set of 100 simulations of an unmitigated *I_F_* 1.5 epidemic compared to plots of epidemics with various combinations of network- and case-based interventions employed.

**Figure 1** **,** *I_F_* 1.5 region, shows that a quarter of the strategies yield results with <10 percent infected (green). However, implementing all case-based interventions without network-based interventions can result, at best, in an infection rate of 35 percent (also see **Figure 4b**). Implementing all network-based interventions alone can reduce the infection rate to 5 percent (also see **Figure 4c**). The nonlinearity in the combination of social distancing interventions is of note. S or CTsd alone are not very effective; however, in combination, they reduce the infection rate to 17 percent, an efficacy far greater than when they are singly imposed. In contrast, combining S and ASsd only reduces the infection rate to 50 percent, less than their linear combination when singly imposed **(** **Figure 1** **,** *I_F_* 1.5 region **)**.

In the unmitigated epidemic, adults are home an average of 3 days. For mitigation strategies that yield infection rates of 10 percent or less, adults stay at home an average of 6 to 19 days (**Figure 2**, *I_F_* 1.5 green region). Importantly, the lowest number of adult days at home is found when implementing all network-based interventions layered with case-based interventions.

Without the use of antivirals, 3 strategies result in infection rates below 10 percent (S+CTsd+ASsd; Q+S+CTsd; Q+CTsd+ASsd), all of which necessitate 12 or more adult days at home. When antivirals are unavailable or ineffective, adding Q to network-based strategies decreases infection rates. For example, with S+CTsd+ASsd, 5% are infected with 14 adult days at home. Adding Q (Q+ S+CTsd+ASsd), 4% are infected and adult days at home are 12. But, with effective antivirals (P or PEx implemented) applying Q is of little additional value and increases the number of days adults are at home from 6 to 7 (also compare **Figure 4d** and **Figure 4e**). This finding differs from the US community mitigation guidance [15]. Interestingly, only 2% of the population must be covered with antivirals using P while 10% coverage is required for PEx, with no added benefit in reduced illness, death or adult days at home. (**Figures 1** **–** [Fig pone-0002606-g002] **3**, *I_F_* 1.5 region)

Thus, for a 1918-like pandemic, the community mitigation strategy that minimizes illness and death (2% infected) also minimizes the average number of adult days at home (6 days). This best strategy combines full social distancing interventions with antivirals used for household prophylaxis and treatment (P+S+CTsd+ASsd) (**Figure 4d**). We also find that with P+S+CTsd+ASsd in place, the minimum population coverage of antivirals is required (2%). Because such a strategy would have considerable societal effects, it would likely be acceptable to a community only in a pandemic with a significant mortality rate.

### Sensitivity of Design for a 1918-like (PSI 4 to 5) Pandemic

**Figure 5:**
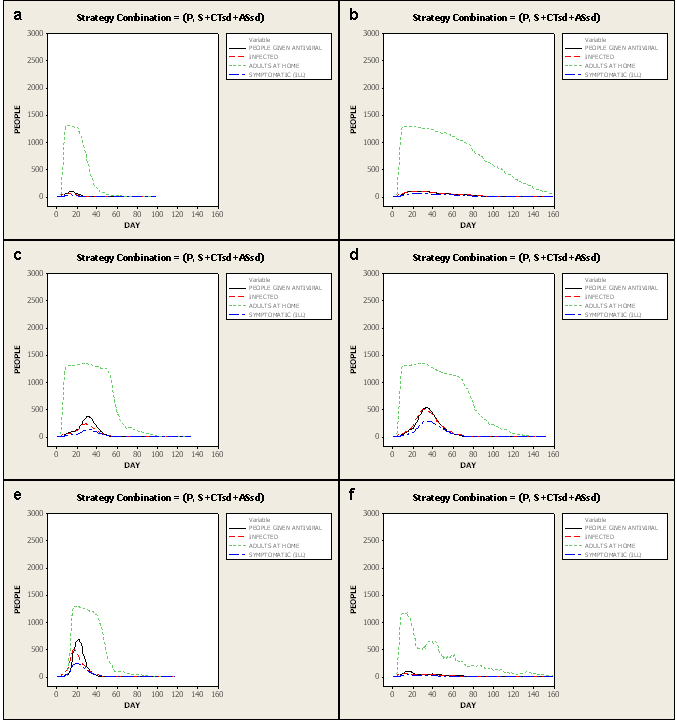
Epidemic effects with perturbed parameter assumptions. Plots of *I_F_* 1.5 epidemics (Ferguson-like disease manifestation) with best strategy applied showing effects of reduced compliance, local-only mitigation, reduced compliance and local-only mitigation, delayed implementation threshold to 100 cases, and relaxed (3-case/7day) rescinding threshold. Plots are averages of all 100 simulations. Comparisons are to the best strategy plot. a—the best strategy found in these simulations (P+S+CTsd+ASsd). b—best strategy applied at 60% compliance. Note the extended duration of epidemic effects (>160 days), length of time antivirals are required, and greatly increased requirements for adult days at home. c—best strategy applied at 90% compliance with local-only mitigation. Note the increase in use of antivirals, extended epidemic effects and increase in adult days at home. d—best strategy applied at 60% compliance with local-only mitigation. Note the higher peaks in numbers of infected and symptomatic near day 40, the increased use and longer duration of need for antivirals and the longer duration of epidemic effects to nearly 160 days. e—best strategy applied with delayed implementation (when 100 cases have occurred). Note the high, early peak of cases and accompanying need for antivirals until the strategy controls the epidemic at day 120. f—best strategy applied with the 3-case/7 day rescinding threshold. Note the extended duration of the epidemic and the erratic downslope of adult days at home as the mitigation strategy cycles off when the rescinding threshold is met and on again when 10 cases occur (the implementation threshold).

Critical results from our evaluation of the sensitivity of the chosen best strategy design for a 1918-like pandemic are displayed in **Figures 5** **and** **6**. Across all model perturbations and extensions, the community mitigation strategy that is best *does not change*, and thus its choice is robust to all considered perturbations. However, some perturbations erode the efficacy more than others and demonstrate the critical enablers of effective mitigation. Full results are available in the extensive set of simulations reported in **Methods S1** **, Tables 9 to 39, and Appendices**. Below we present the results for each, ordered relative to their descending influence on 1) the percentage of the population infected and 2) other measures when the percent infected were the same. Additional comparisons are added where relevant.

**Figure 6:**
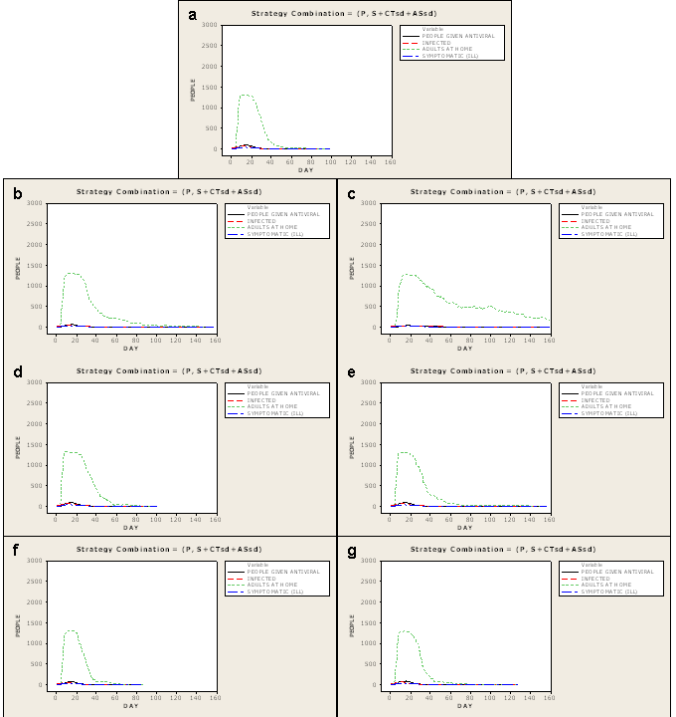
Epidemic effects with extended parameter assumptions. Plots of *I_F_* 1.5 epidemics (Ferguson-like disease manifestation, at 90% compliance, with regional mitigation) showing extensions of parameter assumptions. Plots are averages of all 100 simulations. Comparisons are to the best strategy plot. a—the best strategy found in these simulations (P+S+CTsd+ASsd). b—best strategy under Longini-like assumptions of influenza natural history. Note the similar peak in numbers of infected and symptomatic, but there are additional and extended requirements for antiviral use, adult days at home and longer duration of epidemic effects. c—best strategy with Longini-like with extended period of infectiousness. Note the elongated downslope (and overall increase) of required adult days at home and long duration of epidemic effects. d—best strategy with similar transmission across age classes. Note the minimal increase in duration of adult days at home and duration of epidemic effects. e—best strategy with augmented social networks. Note the slight increase in duration of adult days at home and the significantly increased duration of epidemic effects. f—best strategy with pre-pandemic vaccine targeted to children and teenagers. Note the similarity in curves of required adult days at home and the slightly decreased duration of epidemic effects (from 100 days to 90 days). g—best strategy and pre-pandemic vaccine targeted to adults. Note the lack of benefit on numbers of infected, symptomatic, adult days at home and the increased duration of epidemic effects (from 100 days to 130 days).

#### Implementation Threshold

Delaying implementation until 100 individuals are diagnosed (from 10 diagnosed) erodes efficacy of the best strategy most significantly (**Figure 5e**). The infection rate reaches 13%, adult days at home increase to 12, and an antiviral stockpile of 11 percent coverage is required. Delaying strategy implementation until 30 cases occur also costs the community. More infections occur (5% vs. 2%), more antiviral is required (4%), and the average number of adult days at home increases slightly (to 7 days).

#### Compliance

Reducing compliance to 60 percent from 90 percent also erodes efficacy significantly (**Figure 5b**). Infection rates increase fivefold to 10% as well as do resulting deaths. Almost 7 percent antiviral coverage is required (vs. 2 percent), and adult days at home increases from 6 to 21.

#### Local-Only Mitigation

In a situation of local-only mitigation, the community has less success at controlling epidemics (**Figure 5c and 5d**). Infection rates more than quadruple relative to the situation of regionally applied mitigation (increasing from 2% to 9%). Antiviral requirements increase to 9 percent coverage, and adult days at home double, to 12 days.

#### Rescinding Threshold

Relaxing the rescinding threshold to 3 cases/7 days still results in an infection rate of less than 6% (**Figure 5f**). However, the duration of the epidemic increases from 20 days to 55 days. The longer epidemic duration occurs because the average number of mitigation cycles increases from 1 to 3. The percentage of the population requiring antivirals increases from 2% to 5% and adult days at home increase from 6 to 9 days.

#### Alternative Natural History of Influenza–Longini-like with Extended Period of Infectiousness

The extended period of infectiousness added to the Longini-like manifestation increases the infected population slightly, to 3% from 2% for the Ferguson-like manifestation. Antiviral coverage likewise increases to 3% from 2%. However, the adult days at home quadruple from 6 days to 24 (vs. 10 for the Longini manifestation without the extended period of infectiousness), reflecting the markedly increased epidemic duration (to 117 days compared to 64 days for the original Longini-like manifestation) (**Figure 6c**).

#### Alternative Natural History of Influenza–Longini-like

Changing the influenza natural history to reflect that of Longini (0.67 of infected persons develop clinical illness; individual infectivity is uniform from the pre-symptomatic period through the end of recovery) produces infection rates with network- and case-based strategies implemented very similar to those based on the Ferguson-like manifestation (**Figure 6b**). This holds true with reduced compliance and local-only mitigation as well. The best strategy produces the same results as the Ferguson-like manifestation on infection rates (both 2%); however, the cost in terms of adult days at home increases from 6 days to 10 days. This occurs because of the effects of the increased illness/infection ratio in the Longini manifestation. Antiviral coverage required is slightly higher in the Longini manifestation (3%) for the same reason.

#### Similar Transmission across Age Classes

If similar transmission across age classes is assumed, there is no difference in infection rates compared to when the best strategy is in place in the core network (both 2%) or antiviral coverage needed (both 2%) (**Figure 6d**). The average duration of epidemics is longer for the similar transmission network (27 vs. 20 days for our core network with best strategy). Accordingly, adult days out of work increase from 6 to 7 days. However, there is a significant difference in efficacy with local-only mitigation (9% infected in the core network vs. 25% in the similar transmission network at 90% compliance; antiviral requirements increase accordingly from 9% to 41%). This degradation in efficacy of the best strategy results from the four-fold increase in adult contacts in the work environment. These contacts are assumed to take place with adults from surrounding communities where the epidemic is unchecked.

#### Augmented Social Network

When additional contact groups are added for children and teenagers, less than 2 percent of the population is infected, 2 percent antiviral coverage required, and 7 adult days at home are needed in an epidemic of 26 days' duration with the best strategy applied (**Figure 6e**).

#### Pre-pandemic Vaccine

Adding 50% effective (at prevention of transmission) pre-pandemic vaccine at proposed US stockpile levels of 7% population coverage [20] influences the infection rate slightly when no interventions are instituted. Giving pre-pandemic vaccine only to children and teenagers (700 doses for 2900 children; 24% coverage) decreases the community infection rate most, from 71% to 64%. However, when the best strategy is implemented, pre-pandemic vaccine administered randomly among the population, targeted to children and teenagers, or targeted to adults, has no effect on infection rates, antiviral usage, and adult days at home (**Figures 6f and 6g**).

## Discussion

If death or permanent sequelae of illness from an influenza pandemic could be avoided, it might be acceptable to allow pandemic transmission without intervention. However, history has shown and experts warn that other pandemic consequences are significant—economic losses from decreased work productivity, loss of income, health care surge, and diminution of national security. Because we cannot count on preventing deaths, effects of illness, and societal disruption without deliberately applied interventions, we need strategies that would stop a pandemic. Unfortunately, stopping the global spread of an influenza pandemic may be close to impossible. However, we can design effective community mitigation strategies that work locally much as thinning a forest protects it from devastating fires no matter where lightning may hit.

In this paper, we present a wide-ranging analysis to support the effective, robust design of community mitigation strategies. We examine the tradeoffs of varying uses of social distancing interventions, school closures, antivirals, and pre-pandemic vaccine to locally halt an influenza pandemic in a simulated, explicit, multiply-overlapping network of social contacts forming a stylized community. By focusing on an appropriately contextualized single community, which could be a rural town, a suburb, or a neighborhood within a city, we have evaluated an extensive matrix of mitigation strategies that bracket the proposed US community mitigation guidelines. This exploration enables us to identify and choose community mitigation strategies to implement that minimize illness, death and loss of workforce regardless of transmission to our community from outside and even if antiviral medication or pre-pandemic vaccine were of limited supply or effectiveness. Building on this foundation, we assess the sensitivity of the best mitigation strategy found to variations in influenza natural history, social network configuration, strategy implementation and rescinding thresholds, public compliance, and neighboring community behavior.

For a 1918-like pandemic, the best strategy combines social distancing of all age groups layered with antiviral treatment of ill individuals and antiviral prophylaxis of their household. Implemented rapidly at high compliance and rescinded stringently, this strategy minimized illness to <1 percent of the population, required that <2 percent of the population receive antivirals and limited adult days spent at home to <1 week. This best strategy reflects the recommendations of the US community mitigation guidance, differing only by our finding of the lack of need to quarantine household members of ill persons if antivirals are available and effective for prophylaxis of transmission and illness. The choice of best strategy is robust to changes in the social contact network that removes enhanced transmission by children and teenagers and the number of social contact groups within all age classes and their contacts. The choice of best strategy is also robust to changes in the illness natural history that goes beyond the range currently used in modeling studies found in the literature. However, effectiveness depends on rapid implementation, a strict rescinding criterion, regional implementation and a high degree of public compliance for all interventions.

Based on the findings of our study, we recommend pandemic policy in 3 areas that focus on the critical enablers of resilient community containment: priority for the preparation and implementation of interventions; regional vs. local application of interventions; and targeted administration of pre-pandemic vaccine. In addition, we recommend ongoing study of pandemic behaviors, prevention, and mitigation to reduce existing uncertainties.

The first critical recommendation for policy is that highest priority should be given to the planning and education required for the early triggering and high compliance implementation of network-based interventions such as social-distancing or closing schools, rather than for case-based interventions such as antiviral prophylaxis or household quarantine. For a pandemic similar to 1918, administration of antiviral treatment and prophylaxis at levels above 2 percent population coverage added no benefit and did not remove the necessity of implementing social distancing, closing schools, and reducing contacts within the work environment. Closing schools imposes the largest cost in days adults are at home. However, when the network-based interventions of the best strategy are layered with antiviral treatment and prophylaxis of households, adult days at home can be minimized to an average of 6 days per adult. Our conclusion of the importance of high compliance network-based interventions replicates our past studies [12]–[14], [16] and those of others who have analyzed data available from 1918 [15], [24]–[27]. All of these findings played a role in the recent transition in emphasis within the medical and public health community from planned reliance on antiviral prophylaxis to that of layered non-pharmaceutical interventions [25], [28]. Reducing our strategic dependence on antivirals is further emphasized by studies that show influenza viruses with pandemic potential could exhibit lower sensitivity or develop resistance to available antivirals [29], [30]–[33]. Societal support of parents with children is a critical component for school closure, as families will bear the vast majority of the costs of the resulting adult days at home. Mechanisms including private (company emergency planning and insurance), public (community organization, policy development), and not-for-profit resources could be employed to accomplish a great deal in redistribution of burden.

The second critical recommendation is that a uniform national community mitigation policy should be applied for the benefit of all. Isolated communities implementing effective community mitigation strategies or communities embedded within regions implementing effective mitigation strategies perform equally here. However, simulations in which the community was alone in implementing strategies (local-only mitigation with external contacts through the workplace), show the necessity of regional implementation. Without such regional policy, the best community mitigation strategy still reduces infection rates to less than 10 percent. However, infection and death rates quadruple from their values for the regionally mitigated epidemic, as do antiviral requirements (to 9 percent coverage). The number of days adults are at home also double. Leaving mitigation policy up to individual communities could cost the nation a great deal.

The third critical recommendation for policy is that if pre-pandemic vaccine is available at currently proposed stockpile levels (roughly 7 percent coverage and an assumed 50-percent efficacy at prevention of transmission), the best community mitigation strategy should still be implemented. Simulations show that the most optimal focus of pre-pandemic vaccination in an otherwise unmitigated epidemic at proposed stockpile levels is on children and teens when considering outcomes of infection and clinical illness. However, if our best community mitigation strategy is implemented, pre-pandemic vaccine at proposed stockpiles levels [20] affords little added benefit regardless of the population sector targeted. This pre-pandemic vaccine might then best be targeted to adults who support critical infrastructure (e.g., emergency responders and healthcare, security, and vital utilities workers). Thus, for highest community benefit, individuals who cannot be replaced in infrastructures that must remain operable, such as healthcare and emergency response, should be given the pre-pandemic vaccine. Future studies should evaluate whether a larger pre-pandemic vaccine stockpile and improved vaccine effectiveness would yield enough benefit to change the choice of best community mitigation strategies and our recommendation for policy.

As has been correctly pointed out in an Institute of Medicine review [25], there is uncertainty associated with the predictive ability of pandemic influenza modeling. This uncertainty is fed by our incomplete empirical knowledge of influenza biology and epidemiology, as well as the effectiveness of and public compliance with mitigation interventions. Some of this uncertainty results from the composition of models that are applied to real-world problems. Models can be built to try to emulate the real world and as a result, are extraordinarily complex. Reviewers call for better quantification of model uncertainty [25], [34]. Simulation studies such as ours can avoid much uncertainty by focusing on finding the best strategies for policy consideration and then testing sensitivity of the best strategy choice to perturbations in model parameters and underlying assumptions. Our study elaborates this approach and provides a foundational set of results. This community-scale model focuses on critical components for the local spread of disease—the community structure and use of mitigation strategies. The greatest uncertainties in computational modeling, those associated with the initiation and path of a global pandemic, are avoided. Community-scale analyses can be readily refined in response to evolving knowledge of influenza biology and epidemiology, individual and community behavior, characterization of social contact networks, and mitigation options.

### Disclaimer

The views expressed in this article are those of the authors and do not reflect the official policy or position of the Uniformed Services University of the Health Sciences, Sandia National Laboratories, the Department of Defense, the Department of Veterans Affairs, the Department of Homeland Security, or the United States Government.

## Supporting Information

Methods S1 Supplementary Information that explains the model design and results in detail. Supplementary Information: Effective Robust Design of Community Mitigation for Pandemic Influenza: A Networked, Agent-based Modeling Study.
(0.80 MB PDF)

## References

[1] Ciofi degli Atti ML, Merler S, Rizzo C, Ajelli M, Massari M (2008). Mitigation measures for pandemic influenza in Italy: an individual based model considering different scenarios.. PLoS ONE.

[2] Epstein JM, Goedecke DM, Yu F, Morris RJ, Wagener DK (2007). Controlling pandemic flu: the value of international air travel restrictions.. PLoS One.

[3] Halloran ME, Ferguson NM, Eubank S, Longini IM, Cummings DAT (2008). Modeling targeted layered containment of an influenza pandemic in the United States.. Proc Natl Acad Sci U S A.

[4] McCaw JM, McVernon J (2007). Prophylaxis or treatment? Optimal use of an antiviral stockpile during an influenza pandemic.. Math Biosci.

[5] Riley S, Wu JT, Leung GM (2007). Optimizing the dose of pre-pandemic influenza vaccines to reduce the infection attack rate.. PLoS Med.

[6] Roberts MG, Maker M, Jennings LC, Sertsou G, Wilson N (2007). A model for the spread and control of pandemic influenza in an isolated geographic region.. J R Soc Interface.

[7] Wu JT, Riley S, Fraser C, Leung GM (2006). Reducing the impact of the next influenza pandemic using household-based public health interventions.. PLOS Med.

[8] Ferguson NM, Cummings DA, Cauchemez S, Fraser C, Riley S (2005). Strategies for containing an emerging influenza pandemic in Southeast Asia.. Nature.

[9] Longini IM, Nizam A, Xu S, Ungchusak K, Hanshaoworakul W (2005). Containing pandemic influenza at the source.. Science.

[10] Ferguson NM, Cummings DA, Fraser C, Cajka JC, Cooley PC (2006). Strategies for mitigating an influenza pandemic.. Nature.

[11] Germann TC, Kadau K, Longini IM, Macken CA (2006). Mitigation strategies for pandemic influenza in the United States.. Proc Natl Acad Sci U S A.

[12] Davey VJ, Glass RJ (2008). Rescinding community mitigation strategies in an influenza pandemic.. Emerg Infect Dis.

[13] Glass RJ, Glass LM, Beyeler W (2005). Local mitigation strategies for pandemic influenza.. http://www.sandia.gov/nisac/docs/NISAC_FluMitigationPaperWithFullSOMTables.doc.

[14] Glass RJ, Glass LM, Beyeler WE, Min HJ (2006). Targeted social distancing design for pandemic influenza.. Emerg Infect Dis.

[15] Centers for Disease Control and Prevention (2007). Interim Pre-Pandemic Planning Guidance: Community Strategy for Pandemic Influenza Mitigation in the United States–Early, Targeted, Layered Use of Nonpharmaceuticial Interventions.. http://www.pandemicflu.gov/plan/community/community_mitigation.pdf.

[16] Glass RJ, Min J, Beyeler WE, Glass LM (2007). Design of community containment for pandemic influenza with Loki-Infect.. http://www.sandia.gov/nisac/docs/MatrixReport/RJGMatrixReport.doc.

[17] Leekha S, Zitterkopf NL, Espy MJ, Smith TF, Thompson RL (2007). Duration of influenza A virus shedding in hospitalized patients and implications for infection control.. Infect Control Hosp Epidemiol.

[18] Writing Committee of the Second World Health Organization Consultation on Clinical Aspects of Human Infection with Avian Influenza (H5N1) Virus (2008). Update on avian influenza A (H5N1) virus infection in humans.. NEJM.

[19] Glass LM, Glass RJ (2008). Social contact networks for the spread of pandemic influenza in children and teenagers.. BMC Public Health.

[20] Homeland Security Council (2007). National Strategy for Pandemic Influenza: Implementation Plan, One Year Summary.. http://www.whitehouse.gov/homeland/nspi_oneyear.pdf.

[21] Department of Health and Human Services (2006). HHS pandemic influenza plan, supplement 6, vaccine distribution and use.. http://www.hhs.gov/pandemicflu/plan/sup6.html#S6-II.

[22] Diekmann O, Heesterbeek JAP, Simon L (2000). Mathematical Epidemiology of Infectious Diseases.

[23] Mills CE, Robins JM, Lipsitch M (2004). Transmissibility of 1918 pandemic influenza.. Nature.

[24] Bootsma MC, Ferguson NM (2007). The effect of public health measures on the 1918 influenza pandemic in U.S. cities.. Proc Natl Acad Sci U S A.

[25] Committee on Modeling Community Containment for Pandemic Influenza (2006). Modeling Community Containment for Pandemic Influenza: A Letter Report.. http://www.nap.edu/catalog/11800.html.

[26] Hatchett RJ, Mecher CE, Lipsitch M (2007). Public health interventions and epidemic intensity during the 1918 influenza pandemic.. Proc Natl Acad Sci U S A.

[27] Markel H, Lipman HB, Navarro JA, Sloan A, Michalsen JR (2007). Nonpharmaceutical interventions implemented by US cities during the 1918–1919 influenza pandemic.. JAMA.

[28] Centers for Disease Control and Prevention (2005). Interventions to increase influenza vaccination of health-care workers–California and Minnesota.. MMWR.

[29] De Jong MD, Thanh TT, Kahn TH (2005). Oseltamivir resistance during treatment of influenza A (H5N1) infection.. NEJM.

[30] Hurt AC, Ho HT, Barr I (2006). Resistance to anti-influenza drugs: adamantanes and neuraminidase inhibitors.. Expert Rev Anti Infect Ther.

[31] Hurt AC, Selleck P, Komadina N, Shaw R, Brown L (2007). Susceptibility of highly pathogenic A (H5N1) avian influenza viruses to the neuraminidase inhibitors and adamantanes.. Antiviral Res.

[32] Lipsitch M, Cohen T, Murray M, Levin BR (2007). Antiviral resistance and the control of pandemic influenza.. PLoS Med.

[33] McKimm-Breschkin JL, Selleck PW, Usman TB, Johnson MA (2007). Reduced sensitivity of influenza A (H5N1) to oseltamivir.. Emerg Infect Dis.

[34] Meltzer MI (2008). Pandemic influenza, reopening schools, and returning to work.. Emerg Infect Dis.

