# Supplementary material for: Effective, Robust Design of Community Mitigation for Pandemic Influenza: A Systematic Examination of Proposed US Guidance
Source: PLoS One. 2008 Jul 2;3(7):e2606. doi: 10.1371/journal.pone.0002606 (PMC2432023; doi:10.1371/journal.pone.0002606)
Supplement: Methods S1 — Supplementary Information that explains the model design and results in detail. Supplementary Information: Effective Robust Design of Community Mitigation for Pandemic Influenza: A Networked, Agent-based Modeling Study. (0.80 MB PDF) [file pone.0002606.s001.pdf]

Effective, Robust Design of Community Mitigation for Pandemic Influenza: A Systematic Examination of Proposed U.S. Guidance

Supplementary Information

Victoria J. Davey[‡†], Robert J. Glass[*†], H. Jason Min[*], Walter E, Beyeler[*], Laura M. Glass[§]

[‡]Uniformed Services University of the Health Sciences, Bethesda, Maryland,
and Office of Public Health and Environmental Hazards, Veterans Health Administration, Department of Veterans Affairs, Washington DC.

[*]National Infrastructure Simulation and Analysis Center
Sandia National Laboratories
Albuquerque, New Mexico

[§]Albuquerque Public Schools
Albuquerque, New Mexico

[†]Who have contributed sufficiently to be considered co-first authors

Note: Complete data and the model code are available on request from the authors.

Table of Contents

Methods 1
Loki-Infect Model 1
Base Contact Network 1
Behavioral Rules 4
Alternative Manifestations of Influenza 5
Alternative Networks of Infectious Contacts 7
Choosing Disease Infectivity (I_D) 11
Instigation and Boundary Condition Alternatives 11
Community Mitigation Interventions 12
Simulation Study Design 13

Results 15
Core Simulation Matrix 16
90-Percent Compliance 16
60-Percent Compliance 20
Regional vs. Local-Only Mitigation 22
Perturbations to Core Simulation Matrix 24
Perturbations to Core Simulation Matrix 25
Delaying Implementation Threshold 25
Relaxed Rescinding Thresholds 30
Extensions 35
Longini-like Disease Manifestation 35
Longini-like Disease Manifestation with Extended Duration of Infectiousness 41
Similar Transmission within Children, Teenagers and Adults 46
Augmented Contact Network 51
Administering Pre-pandemic Vaccine 56

Appendix A: Unmitigated Base Case Analysis 61
Observations on Disease Manifestation (comparing Ferguson-like to Longini-like): 61
Observations on I_F for Ferguson- and Longini-like disease manifestations: 63
Observations on 90-percent and 60-percent compliance for Ferguson- and Longini-like disease manifestations: 65

Appendix B: Core Strategy Matrix Results 67

Appendix C: Matrix Perturbation and Extension Results 68

References 70

List of Figures

Figure 1: Example contact network 2
Figure 2: State transitions for natural history of influenza in our model 4
Figure 3: Average population scale infectivity in time for modeled disease manifestations 7

Figure A-1: Epidemic curves for infection by age class and total population (averages of 100 simulations) for Ferguson-like (left) vs Longini-like (right) disease manifestations. Tick refers to day of epidemic. 62
Figure A-2: Epidemic curves for numbers of individuals infected in population of 10,000 for Ferguson-like and Longini-like disease manifestation at I_F 1.0 and 1.5 (averages of 100 simulations). Tick refers to day of epidemic. 62
Figure A-3: Number of adult days at home by day for Ferguson-like and Longini-like disease manifestations at I_F 1.0 and 1.5 (averages of 100 simulations). Tick refers to day of epidemic. 63
Figure A-4: Epidemic curves for number of infected individuals by I_F for Ferguson-like and Longini-like disease manifestations. 64
Figure A-5: Results for 100 simulations (Ferguson-like disease manifestation) on outcome measures of total infected, adult days out, peak infected, peak symptomatic, length of epidemic (tick), and time to peak infections by I_F. Each point represents the results of a single simulation. 64
Figure A-6: Results for 100 simulations (Longini-like disease manifestation) on outcome measures of total infected, adult days out, peak infected, peak symptomatic, length of epidemic (tick), and time to peak infections by I_F. Each point represents the results of a single simulation. 65
Figure A-7: Epidemic curves for infection rates for Ferguson-like (left) and Longini-like (right) disease manifestations by I_F and level of compliance (60 or 90%). Tick refers to day of epidemic. 66

List of Tables

Table 1: Base Community Structure 3
Table 2: Augmented community structure 9
Table 3: Mitigation interventions 12
Table 4: Combinatory strategy matrix: Case-based as rows, network-based as columns 13
Table 5: Simulation Design 14
Table 6: Situation of no or ineffective antivirals, 90% compliance, I_F 1.5 18
Table 7: Core containment strategy combination matrix for infection rates, regionally mitigated, 90 percent compliance 19
Table 8: Situation of no or ineffective antivirals, 60% compliance, I_F 1.5 20
Table 9: Containment strategy combination matrix for infection rates, regionally mitigated, 60 percent compliance 21
Table 10: Containment strategy combination matrix for infection rates, local-only mitigation, 90 percent compliance 23
Table 11: Containment strategy combination matrix for infection rates, local-only mitigation, 60-percent compliance 24
Table 12: Implementation threshold delayed to 100 diagnosed for infection rates, regionally mitigated, 90-percent compliance 26
Table 13: Implementation threshold delayed to 100 diagnosed for infection rates, regionally mitigated, 60-percent compliance 27

Table 14: Implementation threshold delayed to 100 diagnosed for infection rates, local-only mitigation, 90-percent compliance ... 28
Table 15: Implementation threshold delayed to 100 diagnosed for infection rates, local-only mitigation, 60-percent compliance ... 29
Table 16: Rescinding threshold relaxed to 3 cases/7days, for infection rates, regional mitigation, 90-percent compliance ... 31
Table 17: Rescinding threshold relaxed to 3 cases/7days, for infection rates, regional mitigation, 60-percent compliance ... 32
Table 18: Rescinding threshold relaxed to 3 cases/7days, for infection rates, local-only mitigation, 90-percent compliance ... 33
Table 19: Rescinding threshold relaxed to 3 cases/7days, for infection rates, local-only mitigation, 60-percent compliance ... 34
Table 20: Longini-like disease manifestation for infection rates, regionally mitigated, 90-percent compliance ... 37
Table 21: Longini-like disease manifestation for infection rates, regionally mitigated, 60-percent compliance ... 38
Table 22: Longini-like disease manifestation for infection rates, local-only mitigation, 90-percent compliance ... 39
Table 23: Longini-like disease manifestation for infection rates, local-only mitigation, 60-percent compliance ... 40
Table 24: Longini extended infectiousness, for infection rates, regional mitigation, 90-percent compliance ... 42
Table 25: Longini extended infectiousness, for infection rates, regional mitigation, 60-percent compliance ... 43
Table 26: Longini extended infectiousness, for infection rates, local-only mitigation, 90-percent compliance ... 44
Table 27: Longini extended infectiousness, for infection rates, local-only mitigation, 60 percent compliance ... 45
Table 28: Similar transmission within children, teenagers and adults for infection rates, regionally mitigated, 90 percent compliance ... 47
Table 29: Similar transmission within children, teenagers and adults for infection rates, regionally mitigated, 60 percent compliance ... 48
Table 30: Similar transmission within children, teenagers and adults for infection rates, local-only mitigation, 90-percent compliance ... 49
Table 31: Similar transmission within children, teenagers and adults for infection rates, local-only mitigation, 60 percent compliance ... 50
Table 32: Augmented Contact Network, for infection rates, regional mitigation, 90-percent compliance ... 52
Table 33: Augmented Contact Network, for infection rates, regional mitigation, 60 percent compliance ... 53
Table 34: Augmented Contact Network, for infection rates, local-only mitigation, 90 percent compliance ... 54
Table 35: Augmented Contact Network, for infection rates, local-only mitigation, 90 percent compliance ... 55
Table 36: Children and teenager targeted pre-pandemic vaccination on infection rates, regionally mitigated, 90 percent compliance ... 57

Table 37: Children and teenager targeted pre-pandemic vaccination on infection rates, regionally mitigated, 60 percent compliance 58
Table 38: Children and teenager targeted pre-pandemic vaccination on infection rates, local-only mitigation, 90 percent compliance 59
Table 39: Children and teenager targeted pre-pandemic vaccination on infection rates, regionally unmitigated, 60 percent compliance 60

Methods

Loki-Infect Model

Loki was developed by the National Infrastructure Simulation and Analysis Center (NISAC) at Sandia National Laboratories to model critical infrastructures and their interdependencies using a generalized networked agent-based approach [1]. Agent-based models treat entities (individuals and groups) explicitly as "agents". Individual agents are endowed with behavioral rules for internal states and interaction with other agents or the external environment. Such models have been applied in a wide range of fields including economics [2], sociology [3], and, more recently, epidemiology [4]. In addition to representing entities as agents, many applications also require representation of the connections between agents as a network. Within the epidemiological context, a number of theoretical studies show the critical importance of the underlying contact network along which an infectious disease spreads [5, 6]. The Loki simulation approach combines both agents and explicit networks [1].

Loki has been applied to generic congestive cascade [7], power grids [8], payment systems [9, 10], social simulation [11], and infectious diseases [12-15]. Loki-Infect is an infectious disease application of Loki in which agents represent individual people and are linked to each other within and among groups to form a contact network reflective of a multiply overlapping, structured community. Loki-Infect specifies behavioral rules for agents, their interaction, and the performance of network links to model the spread of influenza. Community containment strategies are implemented through modifications of these behavioral rules. In context of pandemic influenza, Loki-Infect has been applied to evaluate social distance strategies [12], design targeted social distancing [13], evaluate rescinding criteria for mitigation measures [15], and design community containment strategies [14] .

Base Contact Network

The model creates an explicit social contact network by first specifying groups of given sizes (or ranges of sizes) within which individuals of specified ages interact (for example, school classes, households, and clubs). It also specifies the average number of individuals with which a person has contact within the group to reflect that within any group, cliques form or are imposed (for example, seating in a classroom). Loki-Infect uses this average number to construct a within-group network that can take a variety of forms. For the stylized community simulated here, the model uses either fully connected, random, or ring networks for each group. Random networks are formed by choosing two individuals, at random, within the group and linking them. This connection process is repeated until the number of links within the group yields the specified average (each individual will have a different number of links). The ring is formed by first placing each individual next to a neighbor and linking them to form a complete circle. Additional links are then made to next nearest neighbors and others symmetrically around the ring (see **Figure 1**). Finally, the model gives links within a group an average frequency of contact (contacts per day). With this approach, an explicit contact network can be built straightforwardly from the experience of community members that exhibits the clustered yet "small-world" character [16] and the multiply-overlapping quality of a structured community [17,18].

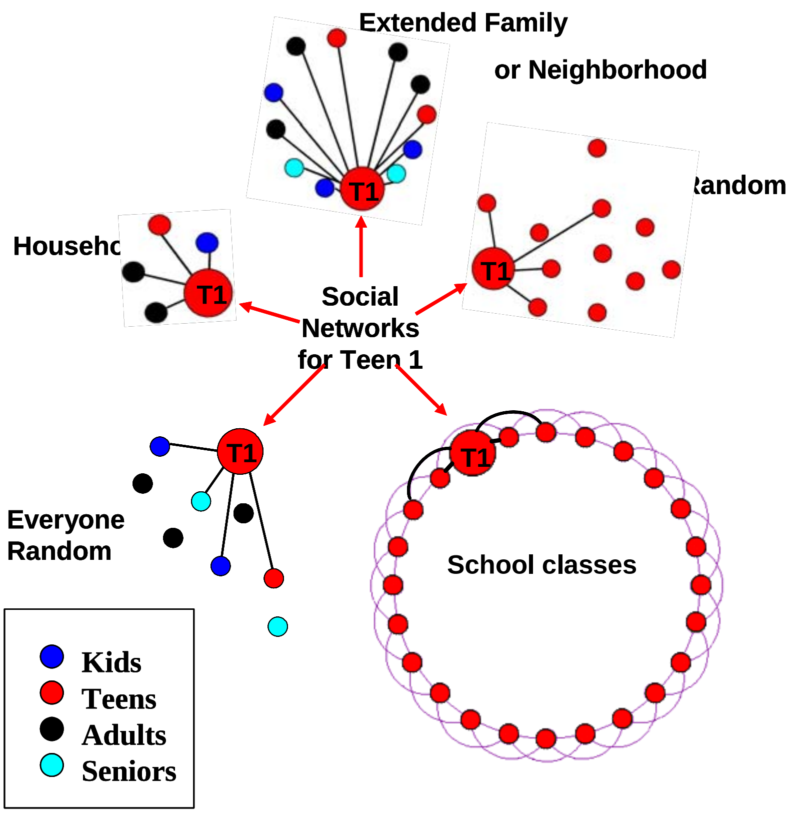

Figure 1: Example contact network

Groups and typical person-to-person links for a model teenager. The teenager (T1) belongs to a household (fully connected network, mean link contact frequency of 6 per day), an extended family or neighborhood (fully connected network, mean link contact frequency of 1 per day), and 6 school classes (ring network with connections to 2 other teenagers on each side, shown as black links; purple links denote connections of other teenagers within the class; mean link contact frequency of 1 per day). Two random networks are also imposed: 1 within the age group (teenager random, average of 3 links per teenager, mean link contact frequency of 1 per day) and 1 across all age groups (overall random, average of 25 links per person [not all links shown], mean link contact frequency of 0.04 per day). Figure 1 from [13].

For this and previous analyses [12-15] we constructed the contact network to represent a stylized community of 10,000 within the U.S. The population conforms to the 2000 US Census and consists of children (0–11 years of age, 17.7 percent), teenagers (12–18 years of age, 11.3 percent), adults (19–64 years of age, 58.5 percent) and seniors (65+ years of age, 12.5 percent) [19]. All individuals belong to multiple groups, each associated with a sub-network of explicit links reflecting their lives within the community**. Figure 1** shows a typical teenager's groups and contact network. **Table 1** reports complete group specifications.

Table 1: Base Community Structure

Groups, membership, networks, and mean frequencies of contact per link. **Table 1** from [13].

**Group (No. of groups in Community)**	**Membership**	**Average No. of links per member**	**Network type and parameters**	**Mean Frequency of contact per link**
**Non-Senior Households (2730)**	1-2 adults<br>0-4 children<br>0-4 teens<br>Mean size 3.13	2.13	Fully connected	6 times a day
**Senior Households (742)**	1-2 seniors<br>Mean size 1.75	0.75	Fully connected	6 times a day
**Extended families or Neighborhoods (800)**	0-2 seniors<br>0-8 adults<br>0-8 teens<br>0-8 children<br>Mean size 12.5	11.5	Fully connected	once a day
**Child classes (69)**	1 class per child,<br>20-35 children in each	4	Ring network,<br>2 neighbors on either side	6 times a day
**Child random (1)**	All children	3	Random network link density 3/1769	once a day
**Teen classes (264)**	six classes per teen,<br>20-35 teens in each	4	Ring network,<br>2 neighbors on either side	once a day
**Teen random (1)**	All teens	3	Random network link density of 3/1129	once a day
**Adult work (351)**	1 work group per adult, 10-50 adults in each	6	Ring network,<br>3 neighbors on either side	once a day
**Adult random (1)**	All adults	3	Random network link density of 3/5849	once a day
**Senior gathering (156)**	1 gathering per senior, 5-20 seniors in each	4	Ring network,<br>2 neighbors on either side	once a day
**Senior random (1)**	All seniors	3	Random network link density of 3/1249	once a day
**Over-all random (1)**	All age classes	25	Random network link density of 25/9999	1/25 a day

Families (adults with children/teenagers) or adults and/or seniors without children/teenagers comprise households. The age class makeup and size of households conform to the statistics of the 2000 Census. All individuals within each household are linked to each other (fully connected sub-network topology) with mean link contact frequencies of 6 contacts per day. Every individual also belongs to one multi-age extended family (or neighborhood) group that has a mean membership of 12.5 and is fully connected with mean link contact frequencies of 1 contact per day.

All children and teenagers go to a preschool or school; children attend a single class per day while teenagers attend 6 (all classes of size 20–35). All adults go to work daily where they interact with other adults (10–50), and all seniors attend senior gatherings (5–20). For contacts within school classes, work, and senior gatherings, we assumed the simplest sub-network topology that imposes local clustering: a ring lattice in which an individual is linked to 2 (for children/teenager classes and senior gatherings) or 3 (adult work) neighboring individuals on each side along the ring (see **Figure 1**). Mean link contact frequencies for children in a single class are 6 contacts per day while teen classes, adult work, and senior gatherings have mean link contact frequencies of 1 contact per day.

To represent additional within-age-class interactions, such as extracurricular activities, playgrounds, bowling leagues, or friends, individuals are explicitly linked to an average of 3 other individuals of the same age class chosen at random at the beginning of the simulation (mean link contact frequency of 1 contact per day). Finally, to emulate a somewhat patterned set of random contacts that come from commercial transactions and other ventures into public spaces, we imposed an explicit random overall network across all age classes with a mean of 25 links per person to yield 1 contact per person per day (mean link contact frequency of 0.04 per day).

Behavioral Rules

The spread of influenza within the contact network is modeled as a series of events. There are 2 classes of events: the transition of an individual between disease states and person-to-person influenza transmission. State transitions follow the natural history of influenza (see **Figure 2**).

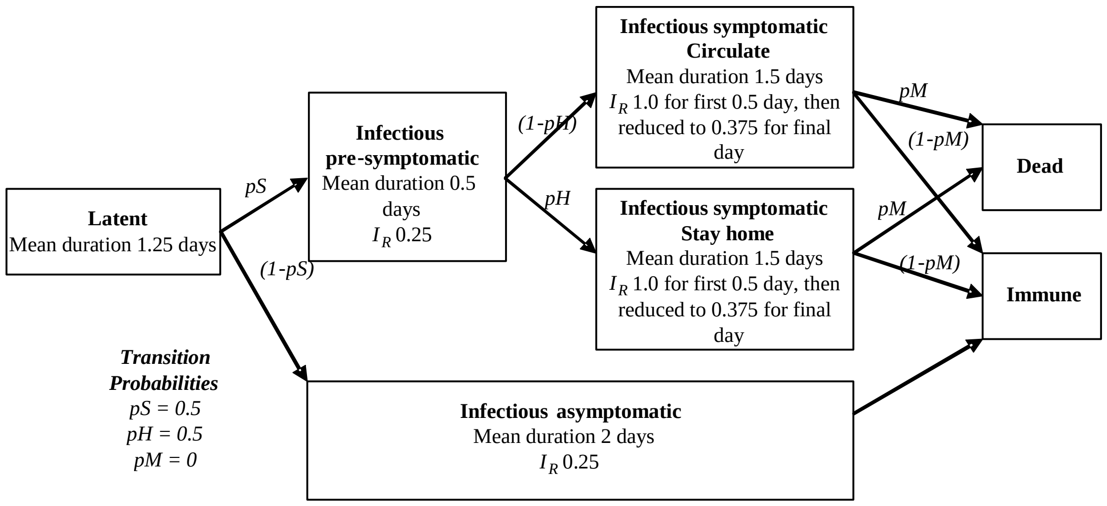

Figure 2: State transitions for natural history of influenza in our model

The duration of each state for a given person is chosen from an exponential distribution. We chose state relative infectivity (I_R) and mean state duration to reflect the infectivity variation of interest. The variations shown in the figure are for the Ferguson-like disease manifestation (see 'Alternative Manifestations of Influenza', below). Figure 2 from [13].

After being infected, the individual enters the latent state. The infected individual then transitions to a pre-symptomatic infectious state or an infectious asymptomatic state with probability *pS* (probability of symptomatic) or 1–*pS*, respectively. Those in whom symptoms develop either stay home with probability *pH* (probability of staying at home if symptomatic), thus influencing their contacts, or continue to circulate with probability 1–*pH*. Infected persons who are asymptomatic continue interacting without behavioral changes. Persons who are symptomatic transition to dead or immune with probability *pM* (probability of death if symptoms occur) or 1–*pM*, respectively, and asymptomatic persons simply transition to immune.

The model evaluates person-to-person transmission events at the beginning of each period during which a person is infectious. Assuming contact events are statistically independent, a transmission time for each of an infectious individual's links within the contact network is chosen from an exponential distribution, with a mean given by the reciprocal of the link's contact frequency scaled by

$$I_D I_R I_A S_P S_A, \qquad \text{Equation 1}$$

Where

I_D = the infectivity of the disease
I_R = the relative infectivity of the disease state
I_A = the relative infectivity of the individual who is transmitting
S_P = the susceptibility of people to the disease (here taken as 1.0)
S_A = is the relative susceptibility of the individual being infected

If the transmission time is less than the time that the individual will be in a particular infectious state (also chosen from an exponential distribution with prescribed means), transmission is scheduled at the chosen time. Otherwise, transmission along that link does not occur during that particular period.

All transmission parameters and contact frequencies may be modified in each of the various states, as well as varied among age classes, by using relative scaling factors such as I_R. In this way, specific disease manifestations and community containment strategies are implemented.

Alternative Manifestations of Influenza

We evaluated 3 alternative manifestations of influenza: one of which conforms closely to Ferguson et al.[20,21], a second that corresponds to Longini and colleagues [22,23] and a third that extends the period of viral shedding of Longini's model for an additional seven days.

Ferguson uses a functional form for infectivity that matches latent period data [24] and viral shedding data [25]. While Ferguson uses this functional form for individuals; we match it at the population scale (averaged across the population). Loki-Infect represents Ferguson's disease manifestation with the following state periods and relative infectivities:

Latent offset (constant 0.75 day)
Latent (mean of 0.5 day)
Infectious pre-symptomatic (mean of 0.5 day, relative infectivity 0.25)
Infectious symptomatic1 (mean of 0.5 day, relative infectivity 1.0)
Infectious symptomatic2 (mean of 1.0 day, relative infectivity 0.375)

Half of infected become symptomatic (*pS* = 0.5), infectious asymptomatic have half the infectivity of symptomatic. Loki-Infect represents asymptomatic infectivity with a constant relative infectivity of 0.25 for a mean of 2 days, starting after the latent period.

Loki-Infect represents Longini's disease manifestation [22, 23] with the following states and relative infectivities:

Latent offset (constant 0.75 day)
Latent (mean of 0.45 day)
Pre-symptomatic (mean of 0.7 day, relative infectivity 1.0)
Symptomatic1 (mean of 3.4 days, relative infectivity 1.0)

Two-thirds of infected develop symptoms (pS = 0.67), infectious asymptomatics have half the infectivity of symptomatics (mean duration 4.1 days, relative infectivity 0.5).

Common to both the Ferguson-like and Longini-like disease manifestations, the analysis uses:

Diagnosis: 0.8 of infectious symptomatics are diagnosed and all diagnosed go home where they remain while sick (*pH* = 0.8; this is age class independent); all non-household contacts are reduced by a compliance factor (60 percent, 90 percent); household contacts remain the same; those that are not diagnosed continue to circulate with no distinction by age class

Babysitting: 1 household adult stays home with a diagnosed child (11 or younger) while they are sick at home or with the child when schools are closed. In this state, all non-household contacts for the babysitter are reduced by a compliance factor (60 percent, 90 percent), household contacts for babysitter are doubled when tending well children (just as for the rest of the household members when schools are closed), but not doubled for tending sick children.

Mortality: *pM* = 0.02, no distinction by age class

Recovery Period: a final recovery period with mean of 7 days is appended to the natural history portrayed in **Figure 2** for every individual who becomes symptomatic, is diagnosed and does not die. Individuals are not infectious during recovery but remain at home and do not go back to school or work.

In the third manifestation, we modified the Longini-like manifestation to extend the period of infectiousness to the end of the recovery period (mean of 7 days, relative infectivity 1.0), as it is possible that the shedding of influenza virus, particularly novel strains like H5N1, may extend beyond the typically expected 5 to 7 days [26, 27]. **Figure 3** shows the infectivity averaged over the population of those who become infected within a typical simulation for all three disease manifestations at an infection attack rate of approximately 50 percent. In addition, viral shedding data of Hayden et al. [25] for normal inter-pandemic influenza are plotted for comparison. Hayden's data are scaled to fit the peak value of infectivity for a disease manifestation. Comparison shows reasonable functional correspondence with average population-scale results for the Ferguson-like manifestation. For Longini-like or Longini-like with extended infectious recovery period disease manifestations, infectivity is lower but longer lasting, reflecting the current uncertainty over viral shedding dynamics of the H5N1 subtype or other influenza viruses with pandemic potential [26].

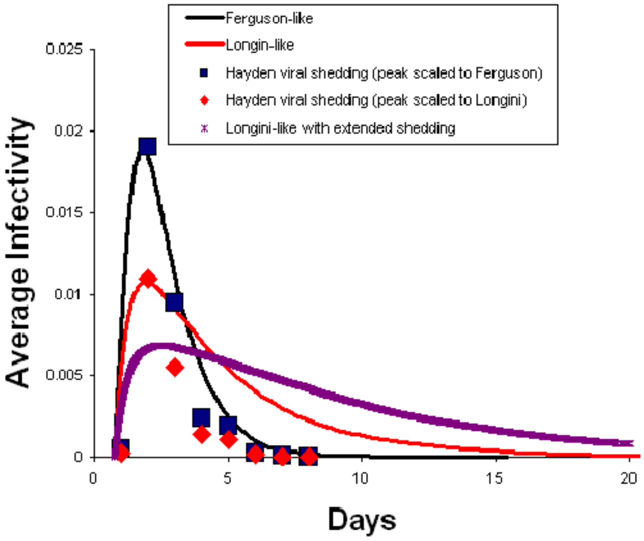

Figure 3: Average population scale infectivity in time for modeled disease manifestations

Note: Viral shedding data from Hayden et al. [25] are shown for comparison scaled to the peak for Ferguson-like and Longini-like disease manifestation.

Alternative Networks of Infectious Contacts

For a given disease manifestation (Ferguson-like, Longini-like, or Longini-like-extended) and disease infectivity (I_D), the network of infectious contacts is dependent on both the contact network and the choice of age class-specific infectivity and susceptibility (I_A and S_A). In the base contact network, we considered I_A and S_A to have equal values within each age class with children, 1.5; teenagers, 1.25; and adults and seniors, 1.0. This assumes that children and teenagers individually are more infective and susceptible, as they have closer contact with others (hugging, wrestling, holding hands, and so forth) and are less likely to wash hands or control coughing or sneezing [28]. As shown previously, this base network of infectious contacts emphasizes transmission among the young and yields age class-specific attack rates within the community reflective of past epidemics (see Figure 6 in [13]).

In this study, we also evaluated two alternatives to our base network. The first adjusts the base contact network and age class-specific infectivity and susceptibility to yield similar transmission within children, teenagers and adults. The second augments the base contact network with additional age-class specific groups to make the contact network more complex and perhaps more representative of today's society.

Similar Transmission within Children, Teenagers, and Adults

The strain of influenza that emerges in the next pandemic may not affect children and teenagers more adversely than adults and seniors. Additionally, the social contact network may have more emphasis on adults within the work environment than currently considered. Both possibilities would reduce the transmission within children and teenagers relative to transmission within the adult population and change the resulting network of infectious contacts. To address this potential, we evaluated an alternative infectious contact network in which the enhanced relative infectivity and

susceptibility for children and teenagers were removed entirely and the number of contacts for adults within the workplace was increased by a factor of 4 to put the adults on par with children and teenagers in schools. While we believe these 2 characteristics to be unlikely, especially in combination, they represent an extreme that likely bounds the uncertainty in the resulting network of infectious contacts.

Augmented Contact Network

As a second alternative, we increased the realism of the contact network by breaking preschoolers (0-5 years of age) out separately (all sent to pre-school during the day) and augmenting the network with additional contact groups. Guided by the survey of Glass and Glass [29], the groups, membership, networks, and mean frequencies of contact per link are given in **Table 2**. Preschooler play groups, child, teen, adult and senior social clubs, teen friends, and adult task groups, were all added to the base contact network. The membership of these groups was constructed by re-sampling the entire pool (e.g., the complete age class) each time a group was created such that individuals could belong to none or more than one of these groups. Children and teenagers were also given an additional random network with an average of 10 links per member (contact frequency once per day) to reflect passing periods within schools. Finally, the number of links within child classes was increased from 4 to 8 and the average frequency of contact per link in extended families or neighborhoods was reduced from 1 per day to 1 per week, also more reflective of data. Simulations using base values for I_A and S_A were found to emphasize transmission within the young much more than the base contact network. A value for I_A and S_A of 1.0 for all age classes (no distinction) yielded age class specific attack rates similar to the base contact network, was reflective of past epidemics, and thus was used in the augmented contact network simulations.

Table 2: Augmented community structure

Groups, membership, networks, and mean frequencies of contact per link. Those groups added to the base contact network are denoted in yellow, modification to base contact groups are denoted in blue.

**Group (# of groups in Community)**	**Membership**	**Average # of links per member**	**Network type and parameters**	**Average Frequency of contact per link**
**Non-Senior Households (2730)**	1-2 adults<br>0-2 preschoolers<br>0-2 children<br>0-4 teens<br>Mean size 3.13	2.13	Fully connected	6 times a day
**Senior Households (742)**	1-2 seniors<br>Mean size 1.75	0.75	Fully connected	6 times a day
**Extended families or Neighborhoods (800)**	0-2 seniors<br>0-8 adults<br>0-8 teens<br>0-4 children<br>0-4 preschoolers<br>Mean size 12.5	11.5	Fully connected	1/7 a day
**Preschooler Classes (52)**	One class per child, 15-20 in each	8	Ring network with radius 4 and rewire probability of 0.5	6 times a day
**Preschooler Play group (177)**	177 groups 3-7 preschoolers in each	5	Random network with link density of 1	once a day
**Preschooler extra random connections (1)**	All preschoolers	3	Random network with link density of 3/884	once a day
**Child classes (35)**	1 class per child,<br>20-35 children in each class	8	Ring network with radius 4 rewire probability of 0.5	6 times a day
**Child passing (1)**	1 passing for children all children	10	Random network with link density of 10/884	once a day
**Child social clubs (88)**	88 groups 5-15 children in each	10	Random network with link density of 1.0	once a day
**Child random (1)**	All children	3	Random network link density 3/884	once a day
**Teen classes (264)**	6 classes per teen,<br>20-35 teens in each class	4	Ring network with radius 2	once a day

**Teen passing (1)**	1 passing periods per day All teens	10	Random network link density 10/1130	once a day
**Teen social clubs (100)**	100 groups 10-35 in each	10	Random network with link density of 0.4347	once a day
**Teen friends (113)**	113 groups 5-15 teens in each	10	Random network with link density of 1.0	once a day
**Teen random (1)**	All teens	3	Random network link density of 3/1129	once a day
**Adult work (351)**	1 work group per adult, 10-50 adults in each	6	Ring network with radius 3	once a day
**Adult task groups (1170)**	1170 Meeting group 3-7 adults in each	5	Random network link density of 1.0	once a day
**Adult social clubs (585)**	585 groups 5-15 adults in each	10	Random network with link density of 1.0	once a day
**Adult random (1)**	All adults	3	Random network link density of 3/5849	once a day
**Senior gathering (156)**	1 gathering per senior, 5-20 seniors in each	4	Ring network with radius 2	once a day
**Senior social clubs (250)**	250 groups with 3-7 seniors in each	5	Random network with link density of 1.0	once a day
**Senior random (1)**	All seniors	3	Random network link density of 3/1249	once a day
**Over-all random (1)**	All age classes	25	Random network link density of 25/9999	1/25 a day

Choosing Disease Infectivity (I_D)

Following the selection of parameter values that define the contact network, disease manifestation, and relative infectivity for various age classes, the overall infectivity of the disease (I_D) is the final and only parameter used to tune the simulations to yield infection rates with different basic reproductive numbers (R_o, or the number of secondary infections produced by one individual in a susceptible population [30]) for an epidemic. For the systematic variation of disease infectivity, we first found a reference I_D that yields approximately a 50-percent infection rate (with a 25-percent illness attack rate for Ferguson-like, and 33-percent illness attack rate for Longini-like disease manifestations) reflective of the 1958 pandemic where the best data exist for age class-specific attack rates [21, 31]. I_D was then scaled by factors (I_F) of 0.75, 1.0, 1.25, 1.5, 2.0, 2.5, and 3.0 to yield both lower and higher infection rates. Note that an I_F of 1.5 yields the current accepted infectivity for the 1918 pandemic, equivalent to an R_o of about 2.0 as estimated by branching factor analysis in this stylized community [13, 32] (**Appendix A** includes branching factor data for simulations of unmitigated epidemics.)

Epidemics of varying severities for a given I_F can be obtained by scaling the mortality rate. A common mortality rate of 0.02, representative of the 1918 pandemic [32], was used here and, because transition to this state within an agent does not influence the outcome of the simulation, varying severities can be obtained from the data directly by scaling the number of deaths that occur.

The I_D for the reference 50-percent infection rate must be chosen independently for each combination of contact network parameters, disease manifestation, and compliance level (compliance influences sick at home and babysitting behavior and, thus, the attack rate) so that comparisons of strategy efficacy may be made evenly across all.

Instigation and Boundary Condition Alternatives

Each simulation is instigated with 10 adults chosen at random (as if a business traveler introduces the virus into the community). With no further introductions from outside, this models a closed community. Mathematically, it also models a fully open community that is in interaction with like communities implementing identical mitigation strategies and similarly seeded with infectious adults. This fully open community can be thought of as one local sub-community that is geographically contiguous with other identical sub-communities to compose a larger city. All contacts in any of the groups outside the household can originate from anywhere within the city.

As a possible worst case and along the lines of recent work by Davey and Glass [15] we also examine the alternative situation where a community is surrounded by a regional population within which no mitigation strategies are implemented. We conceive of contact with this regional population as exclusively through the work environment, and replace contacts along all work links with random contacts from a fully mixed reservoir of external adults. The fraction of those adults who are contagious as a function of time is modeled by the unmitigated epidemic for the given I_F (the regional population is assumed to be uninfluenced by the course of the disease within the local community). Preserving the number of contacts within the work environment, the probability that a contact with one of these contagious individuals will be infectious is modeled by

$$I_D \langle I_R \rangle I_A S_A F, \quad \text{Equation 2}$$

Where

$\langle I_R \rangle$ = the weighted average over symptomatic (reduced by diagnosis) and asymptomatic individuals for the mean contagious period, and

F = the current work frequency scaling for the particular adult agent chosen at random from the community's population.

F reflects the current situation of the adult, such as sick at home or babysitting, and the particular strategy implemented, quarantine or social distancing, all modified by compliance. The relative susceptibility for the person (S_A) could be adjusted if the adult is receiving antiviral prophylaxis. The restriction of external contact to within the normal work environment seems reasonable during a period of pandemic. For example, this could represent a local sub-community embedded within a larger city where all non-household contacts are from the local sub-community *except at work* where contacts are assumed to be with adults from outside the individual's local sub-community.

Community Mitigation Interventions

We applied 8 independent mitigation interventions (S, CTsd, ASsd, Q, T, P, and PEx), as defined in **Table 3**. Each intervention can have varying compliances and thresholds for implementation, yielding an infinite set of combinations. For our analysis, we applied 2 levels of compliance: very good (90 percent) and good (60 percent), and 3 thresholds for intervention implementation: following the diagnosis of 10, 30 and 100 symptomatic individuals within the community.

Table 3: Mitigation interventions

Intervention	Definition
S	Schools closed, all school contacts reduced by 90%, household contacts doubled. One adult from each household with a child (11 or younger stays home from work.
CTsd	Social distancing of children and teenagers. All non-school and non-household contacts with or between children and teenagers reduced by 60% and 90%, household contacts doubled.
ASsd	Social distancing of adults and seniors. All non-household, non-work contacts within and between adults and seniors reduced by 60% and 90%, work contacts reduced by 50%; household contacts doubled.
Q	Household quarantine for 10 days once an individual in the household is diagnosed, all non-household contacts reduced by 60% or 90%, household contacts doubled.
T	Antiviral treatment. Individual given antiviral course with probability (60%, 90%) for 5 days immediately after diagnosis, reduces transmissibility by 60% [20,21]
P	Household member antiviral prophylaxis. Household members given an antiviral with probability (60% , 90%) for 10 days starting immediately after household reference case is diagnosed, reduces infection susceptibility by 30%, reduces probability of clinical illness by 65%, reduces infectivity by 60% [20, 21].
PEx	Extended contact prophylaxis. Household members, workplace contacts, school contacts, and neighborhood/extended family contacts given antiviral with probability (60%, 90%) for 10 days starting immediately after reference case diagnosed; reduces infection susceptibility by 30%; reduces probability of clinical illness by 65%; reduces infectivity by 60%. (Note that school and workplace contact rates used for PEx are much less than the entire school or work groups.)

For a given compliance level, each intervention is implemented separately or in combination to yield a full matrix of combinations for evaluation. Because antiviral interventions T, P, and PEx are nested, with P necessarily incorporating T and PEx necessarily incorporating both T and P, a combinatory matrix with 64 cells is the result. We organize this matrix as in **Table 4** with case-based interventions (Q, T, P, PEx) in columns and network-based interventions (S, CTsd, ASsd) in columns. Case-based interventions act on individuals related to a particular diagnosed case to interrupt transmission. Network-based interventions alter transmission by changing social contact patterns in networks at large. Strategies are combinations of interventions.

Table 4: Combinatory strategy matrix: Case-based as rows, network-based as columns

	**None**	**ASsd**	**CTsd**	**CTsd, ASsd**	**S**	**S, ASsd**	**S, CTsd**	**S, CTsd ASsd**
**None**	None							
**T**								
**Q**								
**P**								
**Q,T**								
**Q,P**								
**PEx**								
**Q,PEx**								All

Community mitigation strategies were rescinded based on the number of newly diagnosed individuals within a 7 day period (corresponding to 2 to 3 generation times of influenza, depending on the disease manifestation). A recent condition study by Davey and Glass [15] revealed that ceasing two social distancing measures, all community sequestering or child sequestering, with no newly diagnosed individuals in 7 days was sufficiently effective to contain an epidemic. Employing this rescinding threshold reduced days adults were home from work by 6% to 32%, in mild and severe epidemics, respectively, as compared to continuing the measures until the last incident case was recovered or dead. Here, we considered two rescinding thresholds, 0 new cases and 3 new cases in 7 days. Subsequent to rescinding, if the number of newly diagnosed individuals were to rise above the implementation threshold (10, 30, or 100 depending on the simulation), mitigation strategies were reapplied and a second mitigation cycle began. If required, additional cycles based on these beginning and ending conditions were implemented until no infected individuals remained within the community.

Simulation Study Design

We designed our simulation study to have three levels: a core, an examination of robustness through perturbations, and an examination of extensions to the core. Simulations were designed as shown in **Table 5.** In our core simulations, we carried out the full matrix with 64 intervention combinations, at 7 I_F, at 2 levels of compliance, and under 2 boundary conditions (of regional or local-only mitigation) with the common features of the Ferguson-like disease manifestation, implementation threshold of 10 diagnosed cases, and rescinding threshold of 0 cases/7 days. The core matrix

constitutes 1,796 parameter combinations spanning a range of illness severity levels (I_F from .75 to 3) reflecting seasonal-like flu outbreaks to pandemics twice as infectious as the 1918 pandemic.

Core parameters were then perturbed by a set of possible alterations in application of strategies through delays of implementation to relaxation of rescinding thresholds. Extensions were explored by changing the disease manifestations (Longini-like and Longini-like-extended), the contact network (similar transmission and augmented contact network) and the availability of pre-pandemic vaccine. Three pre-pandemic vaccination interventions were based on 7-percent coverage of a 50-percent effective vaccine administered 1) randomly, 2) targeted to children and teens, or 3) targeted to adults. For each of these target groups, the vaccine was administered before the initial seeding of infected adults. In total, 10 additional sets of 1,796 parameter combinations were constructed for these perturbations and extensions.

To capture the stochastic variability that is inherent and expected from community to community and epidemic to epidemic due to the variability of social network structure, individual contacts, initially infected, and influenza transmission biology, we conducted 100 simulations for each combination of parameters with different but statistically similar contact networks. All in all, 1,971,200 simulations were run across the core, perturbations and extensions.

Table 5: Simulation Design

Core	▪ 7 I_F from .75 to 3.0 (typical seasonal influenza to twice the transmissibility of 1918)<br>▪ 90% compliance or 60% compliance<br>▪ Regional mitigation or local-only mitigation<br>▪ Common to all:<br>o Ferguson-like disease manifestation<br>o Implementation of strategies at 10 cases in the community<br>o Rescinding of strategies at 0 cases/7days and if epidemic recurs [10 cases], strategies are re-implemented
Perturbations	▪ Delay of implementation to 30 or 100 cases<br>▪ Rescinding of strategies at 3 cases/7 days; if epidemic recurs [with 10 cases], strategies are re-implemented
Extensions	▪ Longini-like disease manifestation<br>▪ Longini-like disease manifestation with extended infectious recovery period<br>▪ Similar transmission within children, teenagers and adults<br>▪ Augmented contact network<br>▪ Availability of pre-pandemic vaccine: 1) randomly, 2) targeted to children and teens, or 3) targeted to adults

Results

Each simulation yields summary and daily output for a variety of outcome measures in addition to documenting the complete sequence of infectious contacts that take place. All output is automatically written into databases that can be queried by input or output to yield statistics of interest. We have focused on 15 outcome measures across all analyses:

1. Number of simulations that yield epidemics (an epidemic is defined as greater than 1 percent of the population infected)
2. Infection rate
3. Illness attack (symptomatic) rate
4. Deaths
5. Peak infected
6. Time to peak infected
7. Peak symptomatic
8. Time to peak symptomatic
9. Epidemic duration (from implementation threshold to last diagnosed)
10. Total time of effects (from initial seeding to last person recovered)
11. Number of days strategies imposed
12. Number of containment cycles needed
13. Number of external infections
14. Number of antiviral courses given
15. Number of days adults are at home (either sick, quarantined, or tending sick or children sent home from school)

For these measures, we calculated means and standard deviations across each set of 100 runs for a given containment strategy combination, I_F, compliance level, boundary condition (regional or local-only mitigation) and other specific parameters defining the simulation set (see **Table 5**). Only those simulations that created epidemics (defined as greater than 1 percent of the population infected) were used in calculating statistics.

For each set we created an Excel workbook where for each of the 15 outcome measures, there are a set of 4 tables and accompanying 3-dimensional (3D) bar graphs (see **Appendix B** and **Appendix C**). The 4 tables and sets of graphs present results for 90-percent compliance and 60-percent compliance with regional mitigation, and 90-percent compliance and 60-percent compliance where adults are in contact with an external unmitigated epidemic (local-only mitigation).

Strategies are organized in each table and graph, with network-based interventions of S, CTsd, and ASsd in columns (x-axis), and case-based interventions of Q, T, P, and PEx in rows (y- axis) (such as shown in **Table 4**), yielding the 64 possible combinations at each of 7 I_F. To aid in viewing these data, those strategies that yield an infection rate that is 10 percent or less are shaded green and those between 10 percent and 25 percent are shaded pink.

Time series plots for daily measures averaged over the set of 100 simulations may be made for any of the combinations. An example set for an I_F of 1.5 (1918-like) and Ferguson-like disease manifestation that considers the outcome measures of individuals infected, individuals symptomatic, individuals treated with antivirals, and adults at home are attached as PDF files in **Appendix B**.

Detailed results are compiled in the Appendices where Excel workbooks are linked. They are organized as follows:

- **Appendix A** contains analyses of unmitigated simulations, branching factors, generation times, fraction of transmission by age class and group, as well as other statistics for both Ferguson-like and Longini-like disease manifestations.
- **Appendix B** contains the core containment combination matrix results for the Ferguson-like disease manifestation, including the reduced compliance level of 60% and local-only mitigation as shown in **Table 5**.
- **Appendix C** contains the additional Excel worksheets for the perturbations and extensions of the core matrix as shown in **Table 5**.

Below we present and discuss these results. Selected tables from the Excel Workbooks within the Appendices for the number of individuals infected are shown to aid in our presentation. We refer the reader to the full Appendices and their links for additional supporting tables and analyses.

Core Simulation Matrix

The core simulation matrix was carried out with the full 64 interventions or combinations of interventions (strategies), at 7 I_F, at 2 levels of compliance, and under 2 boundary conditions (of regional or local-only mitigation) and the common features of the Ferguson-like disease manifestation, implementation threshold of 10 diagnosed cases, and rescinding threshold of 0 cases/7 days. Below we present results for the two compliances with regional mitigation followed by a comparison of regional to local-only mitigation results.

90-Percent Compliance

Table 7 provides a core matrix for infection rates at I_F from 0.75 to 3.0 that result from interventions/strategies applied with 90-percent compliance where regional mitigation is applied and interventions/strategies are implemented when 10 cases occur in the community. At the lowest I_F (0.75), the efficacy of network-based strategies applied alone increases from ASsd, CTsd, CTsd+ASsd, S, S+ASsd, A+CTsd, to S+CTsd+ASsd. As the I_F increases, case-based strategies that include ASsd increase in efficacy relative to those with CTsd. This is because the branching factor for adults is pushed above 1, there are more adults in the community, and ASsd includes the work environment while CTsd does not include school closings. Applied alone, the efficacy of case-based strategies increases from T through Q, P, Q+T, Q+P, and PEx, to Q+PEx. This order does not change as the I_F increases.

Network-based interventions can more effectively contain infection rates than case-based interventions. For influenza virulence above an I_F of 1.0, case-based interventions alone cannot contain the infection rate below 10 percent. Network-based interventions can accomplish this up to an I_D of 1.5, but combined social distancing strategies (S+CTsd+ASsd) are required.

Combining network-based and case-based interventions across the 64 combinations yields banded green zones of strategies (where infection rates are 10 percent or less) and pink zones (where infection rates are 10 to 25 percent) within each I_F region in the tables. The less-than-10-percent green zone is concentrated in the lower right corner of each I_F region of the table where more strategies are imposed. As can be seen in the tables and the 3D bar graphs, there is a sharp falloff in

infection rate within the pink 10- to 25-percent region with more network- and case-based strategies implemented. In addition, all time scales (epidemic duration, total epidemic time, times to peak infected and symptomatic) increase above unmitigated epidemic levels within the pink region. However, once infection rates are controlled to less than 10% of the population (green zone), epidemic time scales are shorter than those of unmitigated epidemics and continue to decrease as one moves to the lower right corner of each I_F region of **Table 7** (where multiple interventions are implemented, see **Table 4**).

Strategies that are as far as possible into the less than 10-percent infection rate (green) zone are most effective overall. Not only are fewer local epidemics triggered with fewer people infected, symptomatic, or dead, epidemics last much less time with greatly suppressed peaks and, ultimately, a much reduced total societal burden. This is borne out in the number of days that adults are at home; implementing S+CTsd+ASsd costs fewer days than S+CTsd alone. Implementing S is the major component of the number of adult days at home because of adults needing to stay at home with children. But, because we assume in the model that all adults go to work, S places many adults at home who might be able to maintain reasonable work productivity (telecommuting, time shifting, job sharing). Thus, adult days at home under S reflects a worst-case situation. Additionally, teenagers present within the household could provide child care and thus allow the adult babysitter to go to work—an option not reflected in this model.

The measure of adult days at home requires further discussion. The model assumes that all adults participate in a work group. A community where all adults work is unlikely; but some work groups could also be interpreted as activity groups composed of nonworking adults. An adjustment for these nonworking adults could be made in the calculation of workforce reduction by reducing the number of adult days at home by the percentage of unemployed. In addition, every day is a work day in these simulations. To adjust for weekends, 2 days out of 7 could be removed from the outcome measure of the number of days adults are absent from work. ASsd includes a reduction in contact frequency at work of 50 percent of normal. Unlike in Davey and Glass [15], this is assumed to be accomplished within the workplace without adults staying at home. If this can not be accomplished, the number of days adults would be absent from work should be increased by the fraction of the time not present in the workplace times the average duration of the ASsd strategy.

As found in a previous study [15], application of a strategy rescinding threshold of 7 days with no new diagnosed individuals works well. The average generation period for the Ferguson-like manifestation is 2.4 days, so this rescinding threshold is just short of 3 generation periods. On average and across all I_F, additional cycles of strategies implemented are needed only 10 percent of the time and it is rare that more than 1 additional cycle must be imposed.

For strategies resulting in infection rates of 10 percent or less (the green zone), antiviral medications are needed for less than 48 percent of the population. If the PEx intervention is excluded, a maximum of only 8 percent coverage is required within the green zone, and for most of the combinations, it is far less. Applying PEx alone is only effective (green) at an I_F of 0.75. At higher I_F, using PEx can result in the need for 150 percent antiviral coverage of the population, where each individual receives an antiviral course an average of 1.5 times over the course of the epidemic. This greater-than-100-percent coverage is also very ineffective (outside the green or pink regions). If an antiviral had simply been given to everyone in the community early enough, 100 percent coverage would have been more effective than the personnel- and time-intensive contact tracing approach of PEx implemented in the model. The combination of reduced infectivity and reduced susceptibility

assumed to result from antiviral prophylaxis yields a 0.28 reduction in infectious contacts and, with full compliance, could theoretically stop an epidemic with an I_F of 3.0.

The trade-offs between supply of antivirals needed for case-based interventions and the community resolve for network-based interventions can be explored on other outcomes as well. For example, in an I_F 1.5 epidemic, a major objective would be to reduce numbers of deaths as well as to minimize adult days at home. An unmitigated I_F 1.5 epidemic leads to 71 deaths here. If the community applied P only (no networked-based interventions), deaths are reduced by 32% (to 48), an intervention requiring 53% AV coverage of the population and using only 2 adult days at home. If S, CTsd, and ASsd were applied (no case-based interventions) deaths could be reduced by 93% (to 5) using 14 adult days at home. And, if P + S+CTsd+ASsd are applied deaths decrease by 97% (to 2), necessitating 6 adult days at home and requiring less than 2% population coverage of antivirals.

As another example, if the community would only support S without imposing CTsd, but had the means for P and Q, the burden of illness and adult days at home could be compared. In an I_F 1.5 epidemic applying S alone, 31% of the population would be ill (vs. 36% in an unmitigated epidemic). With just S + P applied, illness burden is 18%, requiring 36% population coverage of antivirals. With the addition of Q to S + P, the illness burden would decline to 14% with 27% population coverage of antivirals needed. It should be noted that Q, a case-based intervention, helps significantly when limited network-based interventions are in place to interrupt contacts. Q imposes a burden, however, as Q+S+P necessitates 24 adult days at home vs 20 days when S+P are in place.

If there were no antivirals available (or antivirals were determined to be ineffective at limiting illness and transmission) adding Q, the only non-pharmaceutical case-based intervention, to S + CTsd + ASsd helps slightly in an I_F 1.5 epidemic (**Table 6**). This occurs because the increased frequency of household contacts with Q unenhanced by antiviral protection increases disease transmission.

Table 6: Situation of no or ineffective antivirals, 90% compliance, I_F 1.5

	Infection rates, %	Number of Adult days out
S + CTsd + ASsd	5	14
Q + S + CTsd + ASsd	4	12

Table 7: Core containment strategy combination matrix for infection rates, regionally mitigated, 90 percent compliance

For Ferguson-like disease manifestation and implementation of strategies when 10 cases are diagnosed. Case-based strategy combinations on vertical axis, network-focused strategy combinations on horizontal axis. Green shading, infection rates 10 percent or less. Pink shading, infection rates between 10 and 25 percent.

**S** - Schools closed	**CTsd** - Child/Teen social distancing	**ASsd** - Adult/Senior social distancing	**Q** - Household Quantine	**T** - Antiviral Treatment	**P** - Antiviral Prophylaxis	**PEx** - Extended Antiviral Prophylaxis

ID Factor		None	ASsd	CTsd	CTsd,ASsd	S	S, ASsd	S,CTsd	,CTsd,ASs
0.75	None	27.8	18.7	11.1	6.2	2.2	2.1	1.2	1.2
	T	15.6	7.7	3.7	2.4	1.6	1.5	1.2	1.2
	Q	9.8	5.6	2.7	2.4	1.8	1.5	1.3	1.4
	P	7.1	3.8	2.2	1.8	1.6	1.4	1.1	1.2
	Q,T	6.0	3.2	2.2	1.6	1.4	1.3	1.2	1.2
	Q,P	3.3	3.0	1.7	1.6	1.5	1.3	1.2	1.3
	Pex	2.5	2.1	1.5	1.5	1.5	1.3	1.1	1.1
	Q,Pex	2.7	1.9	1.4	1.4	1.2	1.2	1.0	1.1
1.00	None	49.6	38.4	39.4	30.1	22.7	13.0	2.0	1.7
	T	41.1	30.6	27.4	18.2	6.7	4.0	1.6	1.3
	Q	36.0	26.6	20.1	13.2	7.2	4.2	1.7	1.4
	P	31.9	21.9	12.1	7.3	2.8	2.5	1.4	1.3
	Q,T	28.2	18.8	9.2	6.4	3.3	2.1	1.3	1.3
	Q,P	25.5	15.3	6.2	4.2	2.5	2.0	1.4	1.3
	Pex	17.8	10.3	6.0	3.7	2.2	1.9	1.4	1.3
	Q,Pex	12.9	6.1	3.2	2.6	2.1	1.8	1.3	1.4
1.25	None	62.5	49.2	54.4	43.8	47.2	35.9	5.1	2.8
	T	55.5	42.3	45.8	35.4	32.7	20.9	2.3	1.7
	Q	49.9	40.6	39.6	32.4	28.1	19.6	3.1	2.3
	P	45.9	34.8	33.4	25.5	14.6	7.9	1.8	1.6
	Q,T	43.3	33.5	29.3	21.7	13.5	7.7	2.0	1.7
	Q,P	39.5	30.1	24.3	15.1	6.7	5.0	1.7	1.7
	Pex	31.2	22.9	20.1	13.4	6.7	3.5	1.7	1.4
	Q,Pex	26.9	18.9	11.4	6.4	4.2	2.8	1.6	1.5
1.50	None	71.3	56.3	64.6	52.6	61.0	50.1	16.6	5.3
	T	65.4	50.4	57.4	45.3	51.2	38.8	4.8	2.4
	Q	59.9	49.8	51.7	43.5	45.7	37.1	8.7	4.4
	P	55.7	42.9	46.3	35.9	36.3	22.8	2.9	2.0
	Q,T	52.9	42.7	43.3	34.6	33.6	23.0	3.4	2.4
	Q,P	49.0	38.5	37.8	29.0	27.1	14.6	2.7	2.2
	Pex	39.7	30.5	31.2	23.7	20.5	11.2	2.5	1.8
	Q,Pex	34.9	26.6	23.8	16.2	12.9	7.0	2.5	1.9
2.00	None	82.3	65.3	77.3	63.3	77.9	66.7	52.6	22.4
	T	77.9	60.0	71.9	57.2	71.4	59.0	31.0	6.4
	Q	72.8	60.8	67.0	57.3	66.4	57.4	38.7	21.1
	P	69.2	53.1	62.3	49.4	59.6	47.0	12.5	3.9
	Q,T	66.9	54.4	60.0	49.9	57.4	47.2	17.7	6.4
	Q,P	61.8	49.4	54.8	43.9	51.4	39.4	8.6	4.0
	Pex	52.3	40.2	46.1	35.6	41.4	31.6	6.6	3.4
	Q,Pex	47.1	36.5	39.8	30.3	34.9	24.6	5.6	3.3
2.50	None	88.5	71.1	84.7	70.0	86.1	76.3	70.3	43.1
	T	85.4	66.2	80.7	64.5	81.6	70.3	56.8	20.0
	Q	80.9	67.9	76.5	65.7	77.2	68.8	60.0	41.3
	P	77.8	60.0	72.5	57.6	72.7	60.4	37.9	8.6
	Q,T	75.8	61.9	70.4	58.7	70.8	60.5	44.3	17.7
	Q,P	71.1	56.6	65.2	53.1	65.3	54.2	30.1	8.1
	Pex	61.3	47.0	56.2	43.8	54.1	43.1	20.4	6.0
	Q,Pex	56.0	43.9	50.3	39.0	47.6	37.8	14.7	5.8
3.00	None	92.4	75.4	89.3	74.7	90.9	82.1	80.6	56.6
	T	90.0	70.5	86.2	69.6	87.8	77.2	71.6	37.6
	Q	86.3	72.6	82.6	71.4	84.1	76.1	72.2	54.5
	P	83.8	64.7	79.3	63.0	80.5	69.0	56.9	19.0
	Q,T	81.9	67.0	77.7	65.1	78.6	69.2	60.0	34.7
	Q,P	77.5	61.9	72.8	59.1	74.0	63.4	49.8	17.9
	Pex	68.0	52.2	63.5	49.6	63.3	51.6	38.0	11.3
	Q,Pex	62.8	49.1	58.3	45.6	57.6	46.6	31.2	9.7

60-Percent Compliance

Reducing compliance to 60 percent reduces the efficacy of case-based and network-based interventions (**Table 9**). Infection rates increase generally, thus pushing green zones (less than 10 percent infected) and pink zones (10 to 25 percent infected) further to the lower right of each I_F region where the most interventions are implemented. The ability of network- and case-based interventions to control infection rates to less than 10 percent (green) are entirely lost for an I_F above 1.5. Control of infection rates to between 10 percent and 25 percent (pink) are lost for an I_F above 2.0 at this diminished compliance.

Relative rankings within network-based interventions are the same as for 90 percent compliance. However, for case-based interventions, Q falls below T and Q+T falls below P in influence on infection rate outcomes. Additionally, antiviral interventions increase in relative efficacy to become more similar to network-based interventions. Some of this increase in relative efficacy of antivirals occurs because when Q or any of the network-based interventions are implemented, the contact frequency within the household is doubled and this doubling is maintained for both 90-percent and 60-percent compliances.

Reducing compliance also lengthens epidemic time scales, but within most of the green zone (less than 10-percent infection rate) the time scales remain at or below those of unmitigated epidemics. Days adults are home also increase (because time scales increase) and are as much as 3-fold higher than the number needed at 90-percent compliance within most of the green zone. While the number of strategy cycles needed is not significantly influenced, the number of antiviral courses needed increases nearly 4-fold within the green zone. At lower compliance, PEx becomes more effective at reducing adult days at home.

The significance of compliance is demonstrated by an examination of an I_F 1.5 epidemic where ideal mitigation strategies are applied. With P + S + CTsd + ASsd implemented at 90-percent compliance, 2% of the population is infected, adults spend 6 days out of work, and 2% population coverage of antivirals is needed. With compliance at 60%, 10% of the population is infected, adults spend 21 days out of work, and 7% population coverage of antivirals is needed. High compliance limits illness, societal burden, and need for antivirals.

If there were no antivirals (or antivirals were ineffective at limiting illness and transmission) adding Q, the only non-pharmaceutical case-based intervention, to S + CTsd + ASsd helps insignificantly when compliance is low in an I_F 1.5 epidemic (**Table 8**).

Table 8: Situation of no or ineffective antivirals, 60% compliance, I_F 1.5

	Infection rates, %	Number of Adult days out
S + CTsd + ASsd	43	21
Q + S + CTsd + ASsd	43	22

Table 9: Containment strategy combination matrix for infection rates, regionally mitigated, 60 percent compliance

For Ferguson-like disease manifestation and implementation of strategies when 10 cases are diagnosed. Case-based strategy combinations on vertical axis, network-focused strategy combinations on horizontal axis. Green shading, infection rates 10 percent or less. Pink shading, infection rates between 10 and 25 percent.

S - Schools closed	CTsd - Child/Teen social distancing	ASsd - Adult/Senior social distancing	Q - Household Quantine	T - Antiviral Treatment	P - Antiviral Prophylaxis	PEx - Extended Antiviral Prophylaxis

ID Factor		None	ASsd	CTsd	CTsd,ASsc	S	S, ASsd	S,CTsd	,CTsd,ASs
0.75	None	28.0	27.3	26.6	24.5	2.8	2.4	1.6	1.4
	T	14.1	14.7	13.2	9.7	1.8	1.5	1.1	1.2
	Q	21.8	21.3	20.6	17.4	2.1	1.9	1.6	1.5
	P	8.6	7.2	6.9	4.7	1.5	1.3	1.1	1.2
	Q,T	7.9	7.9	6.4	5.9	1.7	1.5	1.2	1.2
	Q,P	6.2	4.7	4.0	3.5	1.4	1.3	1.1	1.1
	Pex	3.3	3.1	2.5	2.3	1.3	1.3	1.2	1.1
	Q,Pex	2.6	2.4	2.4	1.9	1.4	1.2	1.1	1.2
1.00	None	50.0	46.7	48.8	44.8	25.1	20.6	6.0	3.8
	T	41.1	38.1	39.5	35.4	9.5	5.9	2.3	1.8
	Q	44.2	43.2	44.0	41.6	16.3	13.7	5.3	3.9
	P	34.1	30.7	30.6	27.1	4.2	3.2	1.6	1.6
	Q,T	35.2	33.7	33.9	31.7	5.0	3.8	2.0	1.9
	Q,P	29.8	27.0	25.7	23.4	2.9	2.4	1.9	1.6
	Pex	20.1	17.4	16.9	13.9	2.5	2.2	1.5	1.5
	Q,Pex	16.3	13.9	13.2	9.8	1.9	1.9	1.5	1.5
1.25	None	62.9	58.4	61.6	57.3	49.8	44.4	31.2	23.4
	T	55.6	51.1	54.4	49.4	37.0	31.1	10.1	5.9
	Q	57.6	55.9	57.6	55.1	42.4	38.9	28.3	21.8
	P	47.9	44.1	46.4	42.0	23.2	17.4	3.9	3.0
	Q,T	49.4	47.6	49.3	46.1	28.4	23.3	8.3	5.6
	Q,P	43.7	41.5	42.1	39.3	17.4	10.9	3.7	3.1
	Pex	32.8	30.2	30.9	27.9	11.2	7.0	2.6	2.3
	Q,Pex	29.8	27.8	27.9	25.6	6.4	4.9	2.7	2.2
1.50	None	71.4	66.1	70.6	65.8	64.3	58.9	51.9	43.3
	T	65.4	59.9	64.2	58.9	54.7	48.5	34.4	23.7
	Q	66.9	64.5	66.6	64.1	57.9	54.3	48.0	42.9
	P	58.1	53.0	56.7	51.7	43.3	36.8	18.4	9.9
	Q,T	59.7	57.4	59.4	56.3	47.0	42.2	30.4	22.8
	Q,P	53.7	50.7	53.1	49.5	37.7	32.0	16.7	10.9
	Pex	41.6	38.2	40.3	36.4	26.9	21.9	7.5	4.7
	Q,Pex	38.3	35.9	37.7	34.3	21.3	16.6	6.4	4.6
2.00	None	82.6	76.6	81.5	76.5	80.0	75.4	73.0	66.0
	T	78.2	71.2	76.8	70.8	73.7	68.2	62.6	53.4
	Q	78.8	76.0	78.4	75.6	75.3	72.5	70.1	66.1
	P	71.5	64.9	70.2	64.2	65.2	58.9	51.2	40.6
	Q,T	73.2	69.8	72.7	69.5	67.8	63.8	59.2	53.1
	Q,P	67.5	63.6	66.8	62.8	60.6	55.4	48.7	40.0
	Pex	54.1	48.9	53.4	48.1	46.8	41.4	33.1	23.5
	Q,Pex	51.1	47.5	50.6	46.6	42.6	37.9	29.9	23.2
2.50	None	89.0	83.4	88.0	83.1	87.9	84.1	83.6	77.9
	T	85.6	78.5	84.3	78.3	83.5	78.8	76.9	69.0
	Q	85.8	82.9	85.4	82.7	84.2	81.9	81.2	77.9
	P	80.1	73.0	78.7	72.6	77.0	71.7	68.3	59.1
	Q,T	81.5	77.8	81.0	77.4	78.9	75.7	73.6	68.9
	Q,P	76.4	72.2	75.9	71.6	73.0	69.0	65.6	58.8
	Pex	62.9	56.9	62.2	56.3	58.9	53.1	48.1	39.7
	Q,Pex	60.1	55.8	59.4	55.0	55.1	50.6	46.5	39.3
3.00	None	92.8	87.8	91.8	87.6	92.2	89.2	89.4	85.0
	T	90.2	83.7	89.1	83.6	89.3	85.1	84.9	78.4
	Q	90.3	87.4	89.8	87.4	89.6	87.6	87.5	84.9
	P	85.7	78.7	84.7	78.6	84.2	79.3	78.2	70.4
	Q,T	86.9	83.1	86.3	83.0	85.4	82.7	82.1	78.1
	Q,P	82.6	78.1	82.0	77.9	80.6	77.0	75.5	70.0
	Pex	69.7	62.8	68.8	62.6	67.2	61.7	58.8	50.6
	Q,Pex	66.6	61.9	66.4	61.6	64.0	59.3	56.9	50.7

Regional vs. Local-Only Mitigation

When our community is placed within a surrounding region that is implementing no or ineffective mitigation strategies, the effectiveness of strategies in our stylized community degrades (**Tables 10 and 11**). This is the result of contacts of adults between communities within the work environment. In general, infection rates rise and green regions in the tables shrink. Even at 90 percent compliance with strategies applied in our community, the efficacy of local-only mitigation strategies declines to be similar to that of 60 percent compliance with regional mitigation in force.

At 60-percent compliance, continued interaction with contagious adults from outside reduces community strategy efficacy enough so that infection rates of less than 10 percent (green zone) cannot be attained at an I_F of 1.5. Even at an I_F of 1.25, green strategies require PEx with nearly 40-percent antiviral coverage of the population. Within the green zones of **Tables 10 and 11**, time scales all approach that of unmitigated epidemics, and days adults are at home increase by a factor of 2 or 3 from regionally mitigated simulations.

Table 10: Containment strategy combination matrix for infection rates, local-only mitigation, 90 percent compliance

For Ferguson-like disease manifestation and implementation of strategies when 10 cases are diagnosed. Case-based strategy combinations on vertical axis, network-focused strategy combinations on horizontal axis. Green shading, infection rates 10 percent or less. Pink shading, infection rates between 10 and 25 percent.

S - Schools closed	CTsd - Child/Teen social distancing	ASsd - Adult/Senior social distancing	Q - Household Quantine	T - Antiviral Treatment	P - Antiviral Prophylaxis	PEx - Extended Antiviral Prophylaxis

0.75	None	27.9	20.0	15.3	9.4	6.6	3.8	3.1	2.2
	T	18.0	10.6	8.6	4.2	4.2	2.7	2.6	1.9
	Q	15.2	8.1	7.6	4.6	4.5	3.1	3.1	2.3
	P	11.0	5.8	5.2	3.1	3.3	2.4	2.4	1.9
	Q,T	9.6	4.2	5.2	3.2	3.5	2.4	2.6	1.8
	Q,P	7.9	4.2	4.5	2.8	3.1	2.3	2.4	1.9
	Pex	6.6	3.5	4.4	2.8	3.0	2.2	2.4	1.9
	Q,Pex	5.1	3.1	3.9	2.6	2.9	2.1	2.3	1.9
1.00	None	49.5	38.7	40.0	31.4	27.8	18.6	9.5	4.9
	T	41.9	31.8	31.1	22.4	18.5	10.4	7.4	3.8
	Q	37.8	29.3	27.4	19.7	18.3	10.4	9.0	4.8
	P	33.8	24.6	22.2	13.7	12.4	6.3	6.3	3.4
	Q,T	31.6	22.6	20.5	12.9	12.6	6.9	7.2	3.7
	Q,P	28.6	19.5	17.6	9.9	10.7	5.8	6.2	3.5
	Pex	22.7	14.7	15.0	9.0	9.5	5.6	5.9	3.3
	Q,Pex	18.9	11.9	12.5	6.7	8.6	5.0	5.8	3.4
1.25	None	62.4	49.6	54.9	44.8	48.7	37.3	19.9	9.8
	T	56.2	43.7	47.6	37.3	37.9	26.5	14.4	6.9
	Q	52.1	42.5	43.9	35.6	36.1	26.6	18.5	9.5
	P	47.7	36.7	38.1	29.0	27.9	17.4	11.9	5.9
	Q,T	45.8	35.8	36.8	27.5	27.5	17.3	13.4	6.8
	Q,P	41.8	32.1	32.2	22.8	23.3	14.4	11.6	5.9
	Pex	33.4	25.4	26.2	18.6	18.6	11.6	10.2	5.3
	Q,Pex	29.8	21.3	22.1	14.1	16.0	10.0	9.9	5.4
1.50	None	71.1	56.8	65.0	53.4	62.4	51.1	33.8	16.7
	T	66.0	51.6	58.9	47.2	54.0	41.6	24.6	11.4
	Q	61.9	51.4	55.6	46.2	50.8	41.1	30.7	17.4
	P	57.7	44.9	50.1	39.0	42.8	30.9	19.7	9.3
	Q,T	55.7	45.3	48.4	38.4	42.0	30.9	22.4	11.4
	Q,P	51.6	40.8	43.7	33.6	37.4	25.4	19.0	9.4
	Pex	42.8	32.7	35.8	27.3	28.8	19.8	15.4	8.1
	Q,Pex	38.5	29.4	31.2	22.4	25.0	16.4	15.1	8.1
2.00	None	82.2	65.6	77.4	64.0	78.1	67.1	57.3	34.6
	T	78.3	61.3	72.7	58.8	72.5	60.3	46.2	23.5
	Q	74.6	62.7	70.0	59.7	69.0	59.5	52.4	34.3
	P	70.9	55.7	65.0	52.3	62.8	50.3	36.4	17.9
	Q,T	69.0	56.8	64.1	52.9	61.9	51.0	41.6	23.1
	Q,P	64.5	52.0	59.0	47.8	56.4	44.9	34.9	18.3
	Pex	55.5	43.3	50.0	39.2	45.1	34.9	26.1	14.5
	Q,Pex	50.7	40.0	45.1	34.9	40.0	30.7	25.2	14.3
2.50	None	88.4	71.5	84.6	70.5	86.3	76.5	72.4	49.9
	T	85.6	67.4	81.2	66.0	82.4	71.2	63.1	36.9
	Q	82.3	69.6	78.8	67.7	79.2	70.5	67.4	49.6
	P	79.3	62.6	74.6	60.5	74.5	62.8	52.6	28.6
	Q,T	77.7	64.4	73.8	62.0	73.9	63.3	57.6	36.8
	Q,P	73.1	59.7	69.1	56.7	68.5	57.5	49.6	28.4
	Pex	64.4	50.5	59.6	47.7	57.0	46.2	36.9	21.1
	Q,Pex	59.6	47.5	54.7	43.6	51.5	41.5	35.5	20.9
3.00	None	92.2	75.5	89.3	75.0	91.0	82.1	81.4	60.2
	T	90.1	71.7	86.6	70.7	88.2	78.0	74.3	48.1
	Q	87.2	74.2	84.3	73.2	85.5	77.3	76.9	60.2
	P	84.9	67.2	81.2	66.0	82.0	70.9	64.3	38.5
	Q,T	83.3	69.5	80.2	67.8	80.9	71.5	68.8	48.4
	Q,P	79.2	65.2	76.0	62.8	76.2	66.2	61.2	38.6
	Pex	71.1	55.8	67.1	53.8	65.5	54.3	46.2	28.1
	Q,Pex	66.3	53.4	62.2	50.3	60.5	50.2	44.0	27.4

Table 11: Containment strategy combination matrix for infection rates, local-only mitigation, 60-percent compliance

For Ferguson-like disease manifestation and implementation of strategies when 10 cases are diagnosed. Case-based strategy combinations on vertical axis, network-focused strategy combinations on horizontal axis. Green shading, infection rates 10 percent or less. Pink shading, infection rates between 10 and 25 percent.

S - Schools closed	CTsd - Child/Teen social distancing	ASsd - Adult/Senior social distancing	Q - Household Quantine	T - Antiviral Treatment	P - Antiviral Prophylaxis	PEx - Extended Antiviral Prophylaxis

ID Factor		None	ASsd	CTsd	CTsd,ASsd	S	S, ASsd	S,CTsd	,CTsd,ASs
0.75	None	27.8	26.5	26.6	23.8	6.4	4.5	4.3	3.0
	T	18.5	16.1	15.9	12.1	4.3	3.0	3.2	2.3
	Q	23.5	21.9	22.4	19.4	5.6	3.9	4.3	2.9
	P	12.9	9.2	10.2	7.1	3.6	2.7	3.1	2.2
	Q,T	13.8	10.6	12.2	9.3	3.9	2.7	3.2	2.3
	Q,P	10.1	7.1	8.7	5.3	3.6	2.5	2.8	2.2
	Pex	7.2	4.8	6.6	4.2	3.3	2.5	2.8	2.1
	Q,Pex	5.9	4.4	5.4	3.4	3.3	2.4	2.8	2.1
1.00	None	49.4	45.9	48.3	44.2	29.9	23.5	16.7	11.1
	T	41.8	38.5	40.3	35.7	19.8	13.6	11.4	7.1
	Q	44.9	42.6	44.3	41.3	23.9	18.5	16.4	11.2
	P	35.2	31.7	33.7	28.7	14.9	9.4	9.1	5.5
	Q,T	37.0	34.3	36.1	31.9	16.3	11.0	11.2	6.9
	Q,P	31.9	28.8	30.6	25.9	12.8	8.6	9.0	5.6
	Pex	23.7	20.5	22.0	17.4	10.9	7.2	8.0	5.0
	Q,Pex	21.3	17.9	20.0	15.6	10.4	6.7	7.7	5.1
1.25	None	62.1	57.5	61.2	56.4	50.3	44.1	36.5	27.2
	T	56.0	51.0	54.8	49.7	40.6	33.6	25.9	16.9
	Q	57.8	55.1	57.9	54.3	44.6	38.9	34.9	27.4
	P	49.5	44.8	47.9	43.1	32.0	24.5	20.2	12.4
	Q,T	51.2	47.9	50.7	46.3	34.9	28.3	24.5	16.9
	Q,P	45.8	42.3	45.3	40.6	28.3	21.3	19.3	12.2
	Pex	35.3	31.5	34.3	29.5	21.5	15.9	14.7	9.5
	Q,Pex	32.8	29.3	31.7	27.4	20.0	14.3	14.8	9.8
1.50	None	71.1	65.5	70.0	64.8	64.1	58.0	52.9	44.0
	T	65.9	60.0	64.6	58.7	56.1	48.9	42.1	31.4
	Q	67.1	63.8	67.0	63.3	58.9	54.0	51.0	43.8
	P	59.4	53.9	58.3	52.7	47.9	39.9	33.7	23.5
	Q,T	61.1	57.4	61.0	56.5	50.1	44.1	40.4	31.0
	Q,P	56.0	51.9	55.3	50.5	43.7	36.6	32.7	23.3
	Pex	44.2	39.4	43.4	38.1	32.8	26.1	23.3	16.5
	Q,Pex	42.0	37.7	41.0	36.3	29.7	24.2	22.9	16.2
2.00	None	82.2	75.8	81.1	75.5	79.7	74.4	72.7	64.8
	T	78.3	71.1	77.0	70.5	74.2	67.9	64.8	55.1
	Q	78.8	75.1	78.6	74.9	75.6	71.3	70.8	65.1
	P	72.8	65.8	71.7	65.1	66.9	60.6	56.3	45.3
	Q,T	74.2	69.8	73.9	69.4	69.1	63.7	62.7	54.8
	Q,P	69.3	64.4	68.9	63.7	63.1	57.0	55.0	45.3
	Pex	57.1	51.0	56.4	50.3	49.5	43.0	39.5	30.4
	Q,Pex	54.5	49.3	54.1	48.8	46.4	40.5	38.6	30.3
2.50	None	88.5	82.4	87.6	82.2	87.5	83.2	83.4	76.8
	T	85.8	78.2	84.5	78.0	83.8	78.3	77.7	69.2
	Q	86.0	82.0	85.5	81.9	84.4	81.1	81.5	76.7
	P	81.1	73.6	80.0	73.2	78.3	72.1	70.8	61.0
	Q,T	82.2	77.6	81.8	77.4	79.9	75.3	75.6	69.1
	Q,P	77.9	72.6	77.5	72.4	74.7	69.5	69.1	61.2
	Pex	66.1	59.0	65.2	58.4	60.8	54.4	52.0	43.1
	Q,Pex	63.3	57.8	63.0	57.3	58.1	51.9	51.0	42.7
3.00	None	92.4	86.8	91.6	86.8	91.9	88.4	89.1	83.8
	T	90.2	83.2	89.1	83.1	89.3	84.8	85.2	78.0
	Q	90.3	86.7	89.8	86.7	89.6	86.9	87.6	83.8
	P	86.6	79.1	85.4	78.9	84.9	79.6	79.5	71.1
	Q,T	87.4	82.9	86.8	82.7	86.1	82.5	83.1	77.7
	Q,P	83.8	78.6	83.2	78.4	81.9	77.3	77.9	71.0
	Pex	72.9	64.8	71.7	64.5	69.1	62.8	61.7	52.6
	Q,Pex	70.1	63.9	69.6	63.5	66.3	60.4	60.2	52.5

Perturbations to Core Simulation Matrix

The core simulation matrix parameters were perturbed by a set of possible alterations in application of strategies through delays of implementation (after 30 and 100 individuals diagnosed) and through relaxation of rescinding thresholds (to 3 cases in 7 days). We compare these results with the core simulation matrix results below.

Delaying Implementation Threshold

In [13] the delay of the implementation threshold for S+CTsd was shown to rapidly erode effectiveness. We also found this to be the case for the full mitigation strategy combination matrix. And, the higher the I_F, the greater the erosion of strategy effectiveness. Delaying implementation of strategies until 100 individuals are diagnosed (**Tables 12 through 15**) demonstrated that significantly fewer mitigation strategies are available to keep infection rates below 25 percent (green and pink zones). Because treatment with antivirals is not influenced by an implementation threshold, those strategy combinations that include treatment (T, P, and PEx) erode less than those that do not.

The average time for strategy implementation is delayed by 6 days (at 30 diagnosed) to 14 days (at 100 diagnosed) at an I_F of 1.0 in the less-than-10-percent infection rate zone (green) (Ferguson-like, 90% compliance, regional mitigation). For an I_F of 1.5, waiting until 30 or 100 cases are diagnosed results in a strategy implementation delay of 4 days (at 30 diagnosed) to 7 days (at 100 diagnosed) (Ferguson-like, 90% compliance, regional mitigation). These delays translate into similar delay periods for peak numbers of infected and symptomatic individuals. Adult days at home generally decrease slightly (by about a day) within the green zones as fewer days are spent minding children sent home from school. However, required antiviral courses significantly increase for both of the lower infection rate zones (green and pink), especially as I_F increases.

Table 12: Implementation threshold delayed to 100 diagnosed for infection rates, regionally mitigated, 90-percent compliance

For Ferguson-like disease manifestation and implementation of strategies when 100 cases are diagnosed. Case-based strategy combinations on vertical axis, network-focused strategy combinations on horizontal axis. Green shading, infection rates 10 percent or less. Pink shading, infection rates between 10 and 25 percent.

**S** - Schools closed	**CTsd** - Child/Teen social distancing	**ASsd** - Adult/Senior social distancing	**Q** - Household Quantine	**T** - Antiviral Treatment	**P** - Antiviral Prophylaxis	**PEx** - Extended Antiviral Prophylaxis

ID Factor		None	ASsd	CTsd	CTsd,ASsd	S	S, ASsd	S,CTsd	,CTsd,ASs
**0.75**	**None**	27.5	19.8	15.9	11.9	7.8	6.8	5.1	4.4
	**T**	15.7	10.8	6.3	5.8	4.5	4.5	3.6	3.6
	**Q**	14.1	10.9	8.2	7.4	6.1	5.6	4.7	4.8
	**P**	8.8	6.8	5.2	4.9	4.2	3.9	3.5	3.5
	**Q,T**	7.6	6.6	5.1	4.5	4.2	4.2	3.6	3.5
	**Q,P**	7.6	5.9	5.1	4.3	4.0	4.1	3.3	3.5
	**Pex**	6.2	5.3	4.4	4.3	4.2	3.8	3.6	3.3
	**Q,Pex**	4.6	4.5	4.4	4.0	3.9	3.9	3.5	3.7
**1.00**	**None**	49.5	38.9	40.2	32.3	28.8	22.1	10.8	8.8
	**T**	41.0	31.5	29.9	23.1	17.2	12.8	7.2	6.3
	**Q**	36.9	30.2	26.3	22.0	17.8	15.8	9.9	8.8
	**P**	32.4	24.4	20.1	16.1	11.9	9.7	6.6	5.8
	**Q,T**	29.5	22.8	18.3	14.5	11.7	10.0	6.7	6.5
	**Q,P**	27.0	20.4	15.3	12.3	9.9	8.5	6.1	5.9
	**Pex**	20.3	15.2	13.1	10.2	9.1	8.0	5.9	5.6
	**Q,Pex**	16.5	12.7	10.4	9.3	8.2	7.5	6.1	5.8
**1.25**	**None**	62.6	50.0	55.2	45.3	49.2	40.2	20.6	15.0
	**T**	55.8	43.5	46.8	37.3	37.0	28.8	12.6	10.0
	**Q**	50.9	42.8	42.6	36.4	35.3	30.1	17.3	14.6
	**P**	46.5	36.2	36.3	28.9	25.8	19.9	10.8	9.6
	**Q,T**	43.8	35.6	33.9	28.0	24.9	20.2	11.3	9.7
	**Q,P**	40.6	31.8	29.7	24.2	21.4	17.0	10.5	9.0
	**Pex**	31.3	25.0	23.6	18.3	16.4	14.0	9.5	8.1
	**Q,Pex**	27.5	21.5	19.6	16.0	14.6	12.8	9.2	8.4
**1.50**	**None**	71.3	57.1	65.0	54.2	62.4	52.4	34.1	23.3
	**T**	65.4	51.3	58.0	46.8	53.4	42.9	22.7	15.1
	**Q**	60.5	51.2	53.8	46.7	49.6	43.1	28.6	22.4
	**P**	56.3	44.4	47.9	39.1	41.1	32.5	16.8	13.0
	**Q,T**	53.8	44.6	45.7	38.4	40.0	32.8	19.0	15.2
	**Q,P**	49.7	39.9	40.8	33.7	34.0	27.6	16.4	13.1
	**Pex**	40.4	32.0	33.1	26.8	26.9	21.0	13.4	11.9
	**Q,Pex**	36.0	28.6	27.8	23.0	23.0	19.0	12.7	11.4
**2.00**	**None**	82.3	66.3	77.7	64.7	78.2	68.3	59.1	40.5
	**T**	78.1	60.9	72.3	58.8	72.1	60.8	45.6	28.4
	**Q**	73.6	62.5	68.4	60.0	68.2	60.4	50.6	39.5
	**P**	69.7	54.8	63.4	51.4	61.5	51.0	33.7	23.0
	**Q,T**	67.6	55.9	61.6	52.6	60.2	51.6	36.9	27.3
	**Q,P**	63.0	51.4	56.7	47.4	54.6	45.7	30.4	22.1
	**Pex**	52.8	41.8	47.4	38.1	43.1	35.6	24.3	17.8
	**Q,Pex**	47.9	39.0	41.6	34.5	38.3	31.9	22.1	17.9
**2.50**	**None**	88.6	72.3	85.1	71.3	86.3	77.2	74.2	54.6
	**T**	85.4	67.3	81.0	66.0	82.2	71.5	64.0	42.3
	**Q**	81.5	69.4	77.5	67.7	78.6	71.0	65.7	53.2
	**P**	78.3	61.8	73.5	59.8	73.7	62.8	51.4	33.7
	**Q,T**	76.5	63.6	71.9	61.3	72.1	63.6	54.9	39.7
	**Q,P**	72.0	58.8	66.9	55.9	67.1	57.9	46.7	32.7
	**Pex**	61.7	49.0	56.9	46.5	55.1	45.6	35.7	25.8
	**Q,Pex**	56.9	46.3	51.8	43.1	49.9	42.1	32.5	24.8
**3.00**	**None**	92.3	76.6	89.7	76.0	91.0	82.8	82.9	64.2
	**T**	90.0	71.9	86.6	71.1	88.1	78.3	75.4	52.8
	**Q**	86.7	74.2	83.5	73.2	84.8	77.8	75.8	62.6
	**P**	84.2	66.7	80.3	65.4	81.1	70.9	65.2	43.0
	**Q,T**	82.5	69.0	78.8	67.4	80.0	71.7	66.9	50.4
	**Q,P**	78.4	64.2	74.4	62.2	75.4	66.4	59.9	42.5
	**Pex**	68.6	54.6	64.4	52.6	64.0	54.1	46.3	32.8
	**Q,Pex**	63.7	52.1	59.4	49.3	58.9	50.4	42.8	32.0

Table 13: Implementation threshold delayed to 100 diagnosed for infection rates, regionally mitigated, 60-percent compliance

For Ferguson-like disease manifestation and implementation of strategies when 100 cases are diagnosed. Case-based strategy combinations on vertical axis, network-focused strategy combinations on horizontal axis with green shading, infection attack rate 10 percent or less; pink shading, infection attack rate between 10 and 25 percent.

S - Schools closed	CTsd - Child/Teen social distancing	ASsd - Adult/Senior social distancing	Q - Household Quantine	T - Antiviral Treatment	P - Antiviral Prophylaxis	PEx - Extended Antiviral Prophylaxis

ID Factor		None	ASsd	CTsd	CTsd,ASsd	S	S, ASsd	S,CTsd	,CTsd,ASs
0.75	None	28.7	26.9	26.5	24.8	8.3	7.6	6.1	5.5
	T	16.1	14.1	13.3	11.1	4.9	4.2	4.0	3.9
	Q	23.6	22.3	21.8	20.7	7.3	7.3	5.8	5.6
	P	10.1	9.8	8.6	7.7	4.4	4.2	3.9	3.8
	Q,T	10.7	10.5	9.6	9.0	4.2	4.5	4.2	4.2
	Q,P	8.2	7.6	7.3	7.5	3.9	4.3	3.6	3.6
	Pex	5.9	5.9	5.7	5.6	3.9	4.1	3.7	3.6
	Q,Pex	5.9	6.0	5.6	5.0	4.0	4.0	3.6	3.4
1.00	None	50.0	46.6	48.9	45.2	30.8	27.3	18.3	15.3
	T	41.0	38.3	39.7	35.7	18.5	16.2	10.6	9.0
	Q	44.4	43.4	44.7	42.3	25.2	23.3	17.8	15.2
	P	34.0	31.4	31.8	28.6	14.0	11.9	8.3	7.7
	Q,T	35.5	34.4	34.8	32.5	14.5	13.6	9.6	8.9
	Q,P	30.2	28.5	28.2	26.1	11.8	10.6	8.6	7.8
	Pex	21.6	20.1	19.7	18.2	9.9	9.3	7.3	6.9
	Q,Pex	18.9	17.7	17.5	16.0	8.8	8.9	7.2	7.0
1.25	None	62.9	58.4	61.9	57.7	51.6	47.1	38.3	32.9
	T	55.6	51.3	54.3	50.0	40.0	35.0	24.0	20.0
	Q	57.9	56.1	57.9	55.5	45.5	42.1	35.4	32.4
	P	48.3	44.9	47.0	42.8	31.3	27.4	17.9	15.4
	Q,T	49.8	48.0	49.5	46.9	33.6	30.2	22.1	19.9
	Q,P	44.6	42.1	43.4	40.4	26.6	23.9	17.2	15.9
	Pex	33.3	31.3	31.7	29.0	19.1	17.5	13.1	11.6
	Q,Pex	30.9	29.1	29.6	26.8	16.9	15.9	12.7	11.6
1.50	None	71.7	66.5	70.7	65.9	65.3	60.5	54.6	48.0
	T	65.5	60.0	64.4	59.1	55.8	50.2	41.6	34.1
	Q	67.0	64.8	67.0	64.5	59.4	56.7	51.7	47.9
	P	58.4	53.9	57.0	52.6	46.4	41.3	31.8	26.0
	Q,T	60.0	57.6	59.8	56.8	49.3	46.3	38.7	33.9
	Q,P	54.3	51.7	53.6	50.5	42.2	37.7	29.9	25.7
	Pex	42.2	39.0	41.2	37.6	30.5	26.9	20.1	17.6
	Q,Pex	39.2	37.3	38.4	35.7	27.6	24.8	20.0	18.5
2.00	None	82.6	77.0	81.7	76.7	80.3	76.0	74.2	68.0
	T	78.0	71.5	76.9	71.1	74.2	69.1	65.1	57.3
	Q	78.9	76.2	78.7	76.1	76.1	73.3	71.8	68.2
	P	71.7	65.9	70.7	65.3	66.7	61.1	56.0	47.9
	Q,T	73.5	70.3	73.0	69.8	68.7	65.4	62.0	57.4
	Q,P	68.0	64.5	67.7	63.7	62.3	58.4	53.0	47.7
	Pex	54.5	50.2	53.9	49.1	47.4	43.3	37.5	32.1
	Q,Pex	51.6	48.7	51.2	47.8	44.7	40.5	35.9	31.8
2.50	None	88.9	83.5	88.1	83.5	88.1	84.4	84.3	79.1
	T	85.6	78.9	84.5	78.6	83.8	79.2	78.4	71.0
	Q	86.0	83.1	85.6	83.1	84.7	82.9	82.0	79.1
	P	80.4	73.8	79.2	73.4	78.0	72.7	70.7	63.1
	Q,T	81.7	78.2	81.2	78.0	79.6	76.6	75.0	71.2
	Q,P	76.9	72.7	76.5	72.7	74.1	70.5	68.1	63.2
	Pex	63.5	57.9	62.9	57.4	59.3	54.5	50.8	44.4
	Q,Pex	60.6	56.8	60.3	56.3	56.1	52.3	49.3	44.5
3.00	None	92.8	88.0	91.9	87.9	92.3	89.3	89.9	85.8
	T	90.3	84.0	89.1	84.0	89.4	85.4	85.5	79.8
	Q	90.5	87.8	89.9	87.7	89.8	88.1	87.9	85.6
	P	86.0	79.5	84.9	79.3	84.6	80.3	79.7	73.0
	Q,T	87.1	83.5	86.5	83.3	85.9	83.3	83.0	79.8
	Q,P	83.1	79.0	82.5	78.7	81.4	78.3	77.4	72.9
	Pex	70.3	64.0	69.5	63.7	67.6	62.4	60.9	53.5
	Q,Pex	67.7	63.4	67.1	63.0	64.4	60.9	58.5	53.8

Table 14: Implementation threshold delayed to 100 diagnosed for infection rates, local-only mitigation, 90-percent compliance

For Ferguson-like disease manifestation and implementation of strategies when 100 cases are diagnosed. Case-based strategy combinations on vertical axis, network-focused strategy combinations on horizontal axis. Green shading, infection rates 10 percent or less. Pink shading, infection rates between 10 and 25 percent.

S - Schools closed	CTsd - Child/Teen social distancing	ASsd - Adult/Senior social distancing	Q - Household Quantine	T - Antiviral Treatment	P - Antiviral Prophylaxis	PEx - Extended Antiviral Prophylaxis

ID Factor		None	ASsd	CTsd	CTsd,ASsc	S	S, ASsd	S,CTsd	,CTsd,ASs
0.75	None	28.2	20.8	17.6	13.9	10.3	8.7	7.2	6.9
	T	19.0	12.7	10.5	8.0	6.9	5.8	5.6	5.4
	Q	17.2	12.2	11.4	9.5	9.1	8.2	7.0	7.0
	P	12.7	8.9	8.1	6.4	6.3	5.7	5.1	5.3
	Q,T	11.3	8.8	8.0	6.7	6.4	5.8	5.4	5.3
	Q,P	10.4	7.3	6.8	6.2	5.8	5.5	5.1	5.3
	Pex	9.3	7.2	6.8	5.9	5.7	5.5	5.2	5.3
	Q,Pex	8.1	6.4	6.7	5.8	5.6	5.8	5.4	5.4
1.00	None	49.4	39.5	41.0	33.1	31.3	24.1	15.6	11.8
	T	42.0	32.8	32.6	25.4	22.1	16.0	12.2	8.7
	Q	39.3	31.5	30.6	24.6	23.7	18.3	15.3	11.9
	P	34.2	26.2	25.2	18.8	17.4	12.4	11.0	8.5
	Q,T	32.6	24.7	23.9	18.2	17.8	13.1	11.6	9.3
	Q,P	29.8	22.1	20.9	15.5	15.8	11.7	10.7	8.2
	Pex	23.5	17.4	18.2	13.8	14.2	11.2	10.4	8.2
	Q,Pex	20.8	15.7	16.0	12.7	13.2	10.3	10.3	8.1
1.25	None	62.2	50.4	55.6	46.1	50.3	40.9	27.8	18.7
	T	56.2	44.6	48.7	39.1	41.2	31.6	21.2	14.2
	Q	52.7	44.4	46.2	38.7	40.5	32.6	26.2	19.3
	P	48.1	38.1	40.1	31.7	31.9	24.3	18.2	12.9
	Q,T	46.5	37.6	38.7	31.1	31.9	24.8	20.0	14.0
	Q,P	42.8	34.1	35.0	27.3	28.7	21.3	17.7	13.0
	Pex	34.6	27.2	29.1	22.6	22.9	18.1	15.7	11.9
	Q,Pex	31.2	24.1	25.6	19.8	21.4	16.6	15.8	11.9
1.50	None	71.1	57.4	65.5	54.6	63.5	53.3	41.3	28.0
	T	66.0	52.3	59.6	48.6	55.5	45.1	32.0	21.0
	Q	62.8	52.7	57.2	48.9	53.7	45.1	38.0	28.2
	P	57.8	46.3	51.6	41.6	45.8	36.0	27.1	18.6
	Q,T	56.6	46.5	50.1	41.6	45.3	36.8	30.0	20.7
	Q,P	52.4	42.5	46.2	37.3	40.8	32.4	26.2	18.2
	Pex	43.4	34.7	38.1	30.3	32.8	25.7	22.2	16.2
	Q,Pex	39.6	31.7	34.1	27.1	30.2	23.9	21.6	16.4
2.00	None	82.1	66.5	77.7	65.1	78.6	68.5	62.3	44.6
	T	78.4	62.2	73.2	60.1	73.2	62.3	52.7	35.1
	Q	75.1	63.8	71.1	61.7	70.6	62.3	57.4	44.7
	P	71.1	56.9	66.2	54.5	64.4	53.4	44.4	29.6
	Q,T	69.8	58.4	65.3	55.3	64.0	54.4	48.5	35.0
	Q,P	65.4	53.9	60.7	50.7	59.0	49.0	42.5	29.6
	Pex	56.2	45.1	51.7	42.2	48.2	39.4	33.8	25.0
	Q,Pex	51.7	42.0	47.7	38.8	44.3	36.4	33.1	24.3
2.50	None	88.5	72.5	85.0	71.7	86.6	77.4	75.7	57.6
	T	85.6	68.4	81.6	67.3	82.8	72.4	67.6	47.8
	Q	82.6	70.9	79.4	69.6	80.3	72.4	70.7	57.2
	P	79.6	63.9	75.5	62.4	75.6	64.8	58.9	41.0
	Q,T	78.1	65.9	74.7	64.1	74.9	65.9	62.9	47.3
	Q,P	74.1	61.6	70.2	59.2	70.4	60.8	56.2	40.6
	Pex	65.2	52.4	61.1	50.2	58.9	49.7	44.9	32.4
	Q,Pex	60.7	49.9	57.1	47.3	54.9	46.3	42.8	32.7
3.00	None	92.2	76.7	89.5	76.2	91.1	82.8	83.6	66.3
	T	90.1	72.9	86.8	72.1	88.4	78.7	77.5	57.1
	Q	87.6	75.6	85.0	74.8	86.1	78.7	79.1	66.2
	P	85.2	68.9	81.6	67.9	82.8	72.7	70.0	50.5
	Q,T	83.9	71.0	80.8	69.8	81.8	73.3	72.6	57.0
	Q,P	80.1	67.2	77.0	65.5	77.9	68.7	66.7	50.5
	Pex	71.6	57.9	68.2	56.2	67.2	57.3	53.7	39.7
	Q,Pex	67.5	55.7	64.2	53.8	63.2	54.2	51.6	39.5

Table 15: Implementation threshold delayed to 100 diagnosed for infection rates, local-only mitigation, 60-percent compliance

For Ferguson-like disease manifestation and implementation of strategies when 100 cases are diagnosed. Case-based strategy combinations on vertical axis, network-focused strategy combinations on horizontal axis with green shading, infection rate 10 percent or less; pink shading, infection rate between 10 and 25 percent.

S - Schools closed	CTsd - Child/Teen social distancing	ASsd - Adult/Senior social distancing	Q - Household Quantine	T - Antiviral Treatment	P - Antiviral Prophylaxis	PEx - Extended Antiviral Prophylaxis

ID Factor		None	ASsd	CTsd	CTsd,ASsd	S	S, ASsd	S,CTsd	,CTsd,ASs
0.75	None	28.1	26.8	26.6	24.6	10.9	9.4	8.5	8.0
	T	18.2	16.5	16.6	13.6	7.4	6.5	5.9	5.4
	Q	23.6	22.7	22.6	20.2	10.4	8.7	8.4	7.4
	P	14.2	11.9	12.3	10.2	6.4	5.9	5.5	5.3
	Q,T	14.6	13.4	13.6	10.8	6.8	6.1	6.0	5.5
	Q,P	11.9	10.6	10.7	8.7	6.4	5.6	5.7	5.2
	Pex	9.0	8.1	8.8	7.2	6.1	5.7	5.8	5.1
	Q,Pex	8.2	7.5	8.0	6.9	6.0	5.5	5.5	5.2
1.00	None	49.5	46.2	48.3	44.8	33.2	28.0	22.6	17.9
	T	42.0	38.7	40.5	36.5	23.5	19.2	16.4	12.4
	Q	45.2	43.1	45.2	41.8	28.8	24.5	22.0	18.2
	P	36.1	32.8	34.3	30.3	19.2	15.4	13.8	10.9
	Q,T	37.5	34.9	36.7	33.1	20.6	16.7	15.8	12.5
	Q,P	32.8	29.8	31.3	27.8	17.8	14.4	13.9	10.8
	Pex	25.1	22.0	24.2	20.8	15.4	12.2	12.5	10.2
	Q,Pex	23.3	20.6	22.1	18.5	14.7	11.9	12.3	9.8
1.25	None	62.1	57.8	61.3	56.9	51.7	46.3	40.8	33.3
	T	56.0	51.2	55.1	50.0	42.9	36.7	30.8	24.1
	Q	58.1	55.4	58.1	54.9	47.0	42.3	39.2	33.6
	P	49.9	45.4	48.7	43.9	35.7	29.9	25.8	19.9
	Q,T	51.5	48.5	51.2	47.0	37.7	33.0	29.3	24.3
	Q,P	46.4	43.3	45.8	41.8	33.0	26.9	25.2	19.9
	Pex	36.7	32.7	35.4	31.1	26.0	21.6	20.7	16.3
	Q,Pex	34.3	30.5	33.5	29.7	24.7	20.3	20.2	16.4
1.50	None	71.2	65.6	70.1	65.1	64.9	59.4	55.7	48.1
	T	66.1	60.2	64.8	59.2	57.2	51.1	45.8	37.4
	Q	67.4	64.2	67.4	63.8	60.2	56.1	54.0	48.1
	P	59.9	54.7	58.8	53.4	49.8	43.5	38.8	31.6
	Q,T	61.6	58.0	61.2	57.2	52.1	47.0	44.2	37.6
	Q,P	56.6	52.6	56.2	51.7	46.5	40.6	38.1	31.1
	Pex	45.5	40.9	44.7	39.5	36.3	31.2	29.5	23.9
	Q,Pex	43.0	39.1	42.7	38.5	34.3	29.5	28.3	23.9
2.00	None	82.3	76.1	81.2	75.7	80.0	75.0	74.3	67.1
	T	78.3	71.3	77.0	70.9	74.7	68.8	66.7	58.4
	Q	79.0	75.5	78.9	75.2	76.1	72.4	72.2	67.3
	P	73.0	66.3	72.0	65.8	68.4	62.0	59.7	50.6
	Q,T	74.5	70.1	74.0	69.9	70.3	65.5	64.8	58.1
	Q,P	69.9	65.2	69.5	64.8	64.8	59.6	58.1	50.5
	Pex	58.0	52.2	57.3	51.4	51.9	46.0	44.2	37.3
	Q,Pex	55.4	50.8	55.3	50.2	49.3	43.8	43.4	36.8
2.50	None	88.6	82.8	87.7	82.6	87.7	83.6	84.0	78.1
	T	85.7	78.7	84.5	78.4	84.1	79.0	78.8	71.3
	Q	86.1	82.3	85.7	82.2	84.9	81.8	82.2	78.2
	P	81.3	74.1	80.2	74.0	79.1	73.4	72.9	64.6
	Q,T	82.4	77.9	82.0	77.8	80.5	76.3	76.7	71.0
	Q,P	78.4	73.4	78.0	73.3	75.8	71.1	71.0	64.5
	Pex	66.8	60.2	66.0	59.6	62.7	56.5	55.6	48.1
	Q,Pex	64.2	59.1	63.9	58.5	59.9	55.0	54.7	47.9
3.00	None	92.5	87.1	91.7	87.1	92.0	88.7	89.6	84.8
	T	90.3	83.6	89.2	83.5	89.4	85.1	85.9	79.3
	Q	90.4	87.0	89.9	86.9	89.8	87.4	88.2	84.8
	P	86.7	79.8	85.7	79.5	85.4	80.5	81.0	73.6
	Q,T	87.6	83.2	87.0	83.1	86.5	83.1	83.9	79.3
	Q,P	84.2	79.2	83.7	79.1	82.7	78.7	79.3	73.6
	Pex	73.4	66.3	72.5	65.8	70.7	64.6	64.7	56.7
	Q,Pex	71.0	65.6	70.6	65.0	68.0	62.6	63.3	56.7

Relaxed Rescinding Thresholds

A previous study comparing 2 social distancing interventions (school closing with child and teen social distancing or with all-community member social distancing) with no available antivirals showed that a rescinding threshold of 3 cases in 7 days led to an extended epidemic duration, a greater requirement of adult days at home, and an overall increase in infections [15]. We find this here as well. **Tables 16-19** show infection rates at the 2 levels of compliance and regional vs. local-only mitigation.

Important to the consideration of relaxed rescinding thresholds are the effects on measures that quantify burden of the epidemic and its mitigation on the community. The duration of an I_F 1.5 epidemic changes from 42 days (unmitigated) to 20 days (mitigated with best strategy of P + S + CTsd + ASsd at 90% compliance, regional mitigation, and a 0 case/7 day rescinding threshold) to 55 days with the 3 case/7 days threshold. The increased epidemic duration occurs because the number of mitigation cycles that must be applied changes from 1 (best strategy) to 3, and doubles to 6 with 60% compliance. The percentage of the population requiring antivirals increases from 2% to 5% and adult days at home increase from 6 to 9 days; at 60% compliance this degrades to 33 days at the 3 case/7 day rescinding threshold.

Table 16: Rescinding threshold relaxed to 3 cases/7days, for infection rates, regional mitigation, 90-percent compliance

For Ferguson-like disease manifestation and implementation of strategies when 10 cases are diagnosed. Case-based strategy combinations on vertical axis, network-focused strategy combinations on horizontal axis with green shading, infection rate 10 percent or less; pink shading, infection rate between 10 and 25 percent.

S - Schools closed	CTsd - Child/Teen social distancing	ASsd - Adult/Senior social distancing	Q - Household Quantine	T - Antiviral Treatment	P - Antiviral Prophylaxis	PEx - Extended Antiviral Prophylaxis

ID Factor		None	ASsd	CTsd	CTsd, ASsd	S	S, ASsd	S, CTsd	S,CTsd, ASsd
0.75	None	28.1	17.9	14.0	8.5	4.8	4.5	2.5	2.2
	T	15.8	8.8	5.1	3.9	2.7	2.4	1.7	1.8
	Q	11.6	8.3	5.7	4.8	3.6	3.2	2.2	2.1
	P	8.4	5.8	3.5	2.8	2.8	2.3	1.6	1.5
	Q,T	6.7	4.5	3.2	3.2	2.4	2.2	1.8	1.9
	Q,P	5.6	4.0	2.8	2.3	2.2	2.3	1.5	1.7
	Pex	4.8	3.4	3.3	2.3	2.2	2.2	1.6	1.6
	Q,Pex	4.1	3.0	2.6	2.1	2.0	2.1	1.7	1.7
1.00	None	49.5	38.6	38.8	30.7	26.1	18.3	6.3	3.8
	T	40.4	30.8	28.4	20.7	12.8	8.7	3.5	2.6
	Q	36.0	27.9	22.5	18.4	12.6	10.1	5.1	4.3
	P	31.5	22.8	17.5	12.6	8.1	6.4	2.5	2.6
	Q,T	28.6	19.9	13.6	10.3	7.5	5.5	2.9	2.7
	Q,P	26.0	17.7	11.5	9.0	5.4	4.3	2.5	2.4
	Pex	19.8	13.9	10.8	8.7	5.8	4.8	2.4	2.1
	Q,Pex	14.9	10.4	7.7	6.1	4.7	4.3	2.5	2.2
1.25	None	62.4	49.4	54.6	43.9	47.1	36.5	14.0	8.2
	T	55.8	42.6	45.8	36.0	34.7	24.0	7.2	4.6
	Q	50.0	40.9	40.1	33.1	30.5	24.0	10.6	8.0
	P	45.7	35.0	34.9	26.0	21.1	14.8	5.4	3.7
	Q,T	43.3	34.2	30.9	23.8	18.9	14.2	5.7	4.4
	Q,P	39.4	30.0	26.5	20.7	15.8	11.7	4.3	3.8
	Pex	31.5	24.6	22.7	18.1	14.8	11.1	5.0	3.5
	Q,Pex	27.4	20.7	18.6	13.4	10.9	9.0	4.4	3.0
1.50	None	71.2	56.3	64.6	52.6	61.6	50.3	27.9	15.6
	T	65.6	50.5	57.4	45.5	51.4	39.1	14.7	7.3
	Q	59.9	49.6	51.9	44.0	46.5	38.7	20.8	14.5
	P	55.7	43.4	46.3	36.3	37.5	28.1	10.5	5.3
	Q,T	53.2	42.9	43.5	35.6	35.6	25.9	10.9	6.4
	Q,P	49.3	38.4	38.5	30.7	30.1	21.1	8.2	5.7
	Pex	39.7	31.5	33.3	26.8	26.8	20.8	7.7	6.0
	Q,Pex	36.4	28.1	27.9	21.8	21.5	15.7	6.7	5.3
2.00	None	82.1	65.2	77.3	63.3	77.8	66.8	54.4	32.0
	T	78.0	60.1	72.0	57.3	71.3	59.0	37.5	17.6
	Q	72.8	61.0	67.1	57.8	66.2	57.5	43.2	30.2
	P	69.1	53.4	62.4	49.5	59.6	47.6	27.8	12.8
	Q,T	66.8	54.6	60.0	49.9	57.2	47.6	27.1	16.9
	Q,P	62.1	49.4	54.6	44.3	52.0	41.6	23.0	14.4
	Pex	52.3	40.8	46.8	36.8	43.9	35.2	20.1	9.8
	Q,Pex	48.1	37.8	42.3	33.1	39.5	31.0	17.4	10.5
2.50	None	88.5	71.0	84.7	70.0	86.2	76.2	70.9	46.3
	T	85.4	66.2	80.7	64.5	81.8	70.1	58.1	29.9
	Q	80.9	67.8	76.5	65.8	77.3	68.9	60.8	44.9
	P	78.0	59.9	72.5	57.5	72.7	60.6	43.8	23.9
	Q,T	75.8	61.9	70.3	58.9	70.8	60.8	47.5	30.2
	Q,P	71.1	56.6	65.4	52.8	65.3	54.5	38.1	24.5
	Pex	61.3	47.5	56.5	45.2	55.4	46.1	37.4	20.9
	Q,Pex	56.4	44.6	51.2	41.9	51.2	42.6	31.3	17.8
3.00	None	92.4	75.3	89.4	74.6	90.9	82.1	80.7	58.0
	T	90.0	70.6	86.2	69.5	87.8	77.4	71.6	42.6
	Q	86.3	72.6	82.5	71.3	84.1	76.1	72.5	55.6
	P	83.9	64.8	79.3	63.2	80.5	69.1	59.3	32.9
	Q,T	81.9	67.1	77.5	65.1	78.8	69.4	61.8	40.9
	Q,P	77.4	62.0	72.9	59.3	74.1	63.6	53.6	31.6
	Pex	68.0	52.4	63.7	50.6	64.1	53.4	49.6	27.7
	Q,Pex	63.2	49.7	59.0	47.6	58.5	49.9	44.1	28.1

Table 17: Rescinding threshold relaxed to 3 cases/7days, for infection rates, regional mitigation, 60-percent compliance

For Ferguson-like disease manifestation and implementation of strategies when 10 cases are diagnosed. Case-based strategy combinations on vertical axis, network-focused strategy combinations on horizontal axis with green shading, infection rate 10 percent or less; pink shading, infection rate between 10 and 25 percent.

S - Schools closed	CTsd - Child/Teen social distancing	ASsd - Adult/Senior social distancing	Q - Household Quantine	T - Antiviral Treatment	P - Antiviral Prophylaxis	PEx - Extended Antiviral Prophylaxis

ID Factor		None	ASsd	CTsd	CTsd, ASsd	S	S, ASsd	S, CTsd	S,CTsd, ASsd
0.75	None	29.0	27.0	27.3	23.9	6.4	5.3	4.1	3.2
	T	16.0	13.4	12.1	10.4	2.5	2.9	2.2	2.0
	Q	22.4	21.1	21.3	19.9	5.2	4.8	3.3	3.5
	P	10.5	8.1	7.5	6.9	2.6	2.5	1.9	2.1
	Q,T	10.5	9.6	8.5	8.2	2.4	2.8	2.4	2.1
	Q,P	7.2	7.7	5.7	5.1	2.5	2.3	2.4	1.8
	Pex	5.4	4.8	4.2	4.0	2.7	2.5	2.0	2.0
	Q,Pex	4.5	4.2	3.3	3.1	2.3	2.2	1.9	1.8
1.00	None	49.5	46.6	48.8	45.1	27.9	23.1	14.6	12.6
	T	40.8	38.0	39.5	35.4	14.9	13.0	6.9	5.5
	Q	44.3	43.2	44.0	41.9	22.0	19.5	14.1	12.5
	P	33.9	31.2	31.1	27.7	10.4	8.4	5.4	4.7
	Q,T	35.6	33.5	34.2	31.5	11.3	9.7	6.1	6.1
	Q,P	29.9	27.9	27.1	24.5	8.5	7.4	5.4	4.4
	Pex	21.5	20.0	19.5	16.9	6.4	6.6	4.6	4.1
	Q,Pex	18.8	17.0	16.2	14.9	5.8	5.8	4.4	3.9
1.25	None	62.7	58.3	61.9	57.3	50.3	44.7	33.9	28.2
	T	55.5	50.9	54.1	49.4	38.1	32.2	19.7	15.4
	Q	57.4	55.9	57.6	54.9	43.0	39.6	31.5	28.1
	P	47.9	44.1	46.2	42.1	27.6	21.8	13.9	11.1
	Q,T	49.7	47.6	49.2	46.4	29.7	26.1	17.8	15.0
	Q,P	44.0	41.5	43.0	39.5	22.6	20.5	13.6	11.1
	Pex	33.0	30.5	32.1	28.8	18.9	15.7	10.6	7.3
	Q,Pex	30.5	28.8	29.5	27.1	15.8	14.2	9.5	8.2
1.50	None	71.6	66.2	70.6	65.7	64.3	59.4	51.9	44.4
	T	65.4	59.8	64.1	58.8	54.4	48.4	36.8	30.1
	Q	66.8	64.4	66.8	64.0	57.8	54.5	48.7	44.0
	P	58.0	53.0	56.8	51.5	43.7	38.0	27.9	20.3
	Q,T	59.6	57.3	59.4	56.4	47.2	42.9	33.9	29.9
	Q,P	53.8	50.9	53.2	49.4	39.1	34.4	25.4	21.1
	Pex	42.0	38.2	40.6	37.0	31.2	27.2	19.6	16.3
	Q,Pex	39.3	36.8	38.3	35.0	28.5	25.1	19.3	17.9
2.00	None	82.7	76.7	81.7	76.5	80.0	75.5	73.0	66.2
	T	78.1	71.2	76.7	70.7	73.8	68.2	62.7	53.9
	Q	78.8	75.9	78.5	75.7	75.2	72.5	70.0	65.9
	P	71.5	65.2	70.2	64.2	65.2	59.4	52.0	42.9
	Q,T	73.2	69.8	72.8	69.5	67.6	64.0	59.2	53.9
	Q,P	67.4	63.5	67.0	62.9	60.5	55.7	49.7	42.9
	Pex	54.1	49.0	53.4	48.6	48.1	44.0	39.1	33.6
	Q,Pex	51.1	47.9	50.9	46.9	45.5	41.8	38.4	33.2
2.50	None	89.1	83.3	88.0	83.2	87.9	84.1	83.7	78.1
	T	85.7	78.5	84.4	78.2	83.6	78.8	77.0	69.1
	Q	85.8	82.9	85.4	82.7	84.3	82.0	81.2	77.9
	P	80.1	73.0	78.7	72.6	77.0	71.5	68.2	59.2
	Q,T	81.5	77.7	80.9	77.5	78.9	75.5	73.7	69.0
	Q,P	76.4	72.1	75.9	71.7	72.8	68.8	65.7	59.3
	Pex	63.0	56.8	62.3	56.5	59.5	53.9	52.5	46.3
	Q,Pex	60.0	55.7	59.7	55.4	56.5	52.8	50.9	45.9
3.00	None	92.8	87.8	91.9	87.7	92.2	89.2	89.4	84.9
	T	90.3	83.6	89.1	83.5	89.2	85.2	84.7	78.4
	Q	90.3	87.5	89.8	87.4	89.5	87.5	87.4	84.7
	P	85.7	78.7	84.7	78.4	84.2	79.4	78.1	70.2
	Q,T	86.9	83.2	86.2	83.0	85.5	82.9	82.0	78.1
	Q,P	82.5	78.0	81.9	77.8	80.7	77.1	75.6	70.3
	Pex	69.6	62.8	68.9	62.5	67.4	62.3	61.3	54.7
	Q,Pex	66.7	62.0	66.3	61.7	64.4	60.7	59.0	54.5

Table 18: Rescinding threshold relaxed to 3 cases/7days, for infection rates, local-only mitigation, 90-percent compliance

For Ferguson-like disease manifestation and implementation of strategies when 10 cases are diagnosed. Case-based strategy combinations on vertical axis, network-focused strategy combinations on horizontal axis with green shading, infection rate 10 percent or less; pink shading, infection rate between 10 and 25 percent.

S - Schools closed	CTsd - Child/Teen social distancing	ASsd - Adult/Senior social distancing	Q - Household Quantine	T - Antiviral Treatment	P - Antiviral Prophylaxis	PEx - Extended Antiviral Prophylaxis

ID Factor		None	ASsd	CTsd	CTsd, ASsd	S	S, ASsd	S, CTsd	S,CTsd, ASsd
0.75	None	28.2	20.4	16.1	12.3	9.5	7.5	5.8	5.3
	T	18.5	12.3	10.1	7.4	6.1	5.6	4.7	4.1
	Q	15.9	11.2	10.2	8.5	7.7	6.7	5.9	5.1
	P	12.0	8.1	7.5	6.0	5.4	4.9	4.6	4.0
	Q,T	10.9	8.1	7.0	5.9	5.7	5.0	4.6	4.2
	Q,P	10.0	6.7	6.5	5.8	5.5	4.8	4.4	4.0
	Pex	8.0	6.7	6.4	5.6	5.3	4.6	4.3	3.9
	Q,Pex	7.1	6.2	6.0	5.1	5.0	4.6	4.3	4.1
1.00	None	49.4	38.9	40.6	31.7	29.2	20.5	12.0	8.4
	T	42.1	32.8	32.2	23.6	20.0	12.7	9.2	6.2
	Q	37.9	29.7	28.4	21.8	20.4	13.8	11.9	8.0
	P	34.0	25.1	23.4	16.3	15.0	10.0	8.3	6.0
	Q,T	31.8	23.4	21.6	15.0	14.6	9.9	8.9	6.3
	Q,P	29.4	20.4	18.7	12.8	12.7	9.0	8.1	5.8
	Pex	23.0	16.7	17.0	12.0	12.2	9.1	7.8	5.7
	Q,Pex	20.1	14.2	14.5	10.3	10.6	8.3	7.7	6.0
1.25	None	62.3	49.7	55.1	45.2	49.0	37.9	23.3	13.2
	T	56.4	44.0	48.1	37.5	39.2	27.9	16.7	9.5
	Q	51.9	42.7	44.5	36.2	37.3	28.7	21.3	13.4
	P	47.9	37.0	38.9	29.4	29.1	20.1	14.1	8.6
	Q,T	45.9	36.4	36.9	28.0	29.0	20.3	15.3	9.7
	Q,P	42.2	32.7	33.1	24.3	25.1	17.1	13.6	8.2
	Pex	34.2	26.5	28.0	20.5	21.1	15.2	12.4	8.0
	Q,Pex	30.4	23.3	24.0	17.5	19.0	13.0	11.7	8.0
1.50	None	71.4	56.8	65.1	53.6	62.7	51.5	36.5	20.5
	T	66.2	51.7	59.2	47.4	54.3	42.4	27.1	14.5
	Q	62.1	51.8	55.9	46.8	51.7	41.9	32.6	21.1
	P	58.0	45.3	50.1	39.8	43.5	32.2	22.0	12.4
	Q,T	56.0	45.3	48.8	39.1	43.3	32.5	24.8	14.3
	Q,P	51.8	41.3	44.3	34.0	38.1	27.3	20.8	12.3
	Pex	43.0	33.6	37.0	29.1	31.4	23.4	18.2	11.1
	Q,Pex	39.3	30.4	33.2	24.7	28.2	19.8	17.4	11.2
2.00	None	82.2	66.0	77.5	64.3	78.3	67.5	58.6	36.9
	T	78.5	61.4	73.0	59.0	72.5	60.5	47.6	26.3
	Q	74.6	62.8	70.3	60.0	69.4	59.9	53.8	37.6
	P	71.1	55.9	65.4	52.8	63.1	51.2	38.9	21.7
	Q,T	69.2	57.1	64.5	53.3	62.4	51.4	43.6	27.1
	Q,P	64.9	52.3	59.5	48.0	56.8	45.7	37.3	21.5
	Pex	55.7	43.7	50.7	40.5	46.5	36.9	30.0	18.5
	Q,Pex	51.3	40.9	46.3	36.8	42.5	34.5	28.6	17.5
2.50	None	88.6	71.8	84.9	70.8	86.4	76.5	72.7	51.1
	T	85.7	67.7	81.5	66.1	82.6	71.3	63.7	39.6
	Q	82.4	69.8	79.0	68.1	79.5	70.5	68.1	51.2
	P	79.4	62.8	74.9	60.8	74.8	62.8	54.3	32.2
	Q,T	78.1	64.7	73.8	62.1	74.0	63.7	58.5	39.7
	Q,P	73.4	60.0	69.3	57.0	68.9	57.9	51.5	32.9
	Pex	64.8	51.0	60.2	48.5	57.4	47.9	40.5	26.0
	Q,Pex	60.1	48.3	55.7	45.6	53.7	44.5	40.0	26.3
3.00	None	92.3	75.7	89.4	75.3	91.1	82.2	81.8	61.3
	T	90.3	71.9	86.6	71.1	88.3	78.1	74.5	50.2
	Q	87.4	74.5	84.5	73.4	85.7	77.4	77.4	61.1
	P	85.0	67.5	81.3	66.3	82.1	71.1	65.7	41.9
	Q,T	83.6	69.9	80.4	68.2	81.2	71.7	69.5	50.0
	Q,P	79.5	65.3	76.2	63.1	76.6	66.2	62.5	41.9
	Pex	71.5	56.2	67.2	54.2	65.9	55.5	49.3	34.0
	Q,Pex	66.5	53.8	63.1	51.6	61.1	52.0	47.8	33.7

Table 19: Rescinding threshold relaxed to 3 cases/7days, for infection rates, local-only mitigation, 60-percent compliance

For Ferguson-like disease manifestation and implementation of strategies when 10 cases are diagnosed. Case-based strategy combinations on vertical axis, network-focused strategy combinations on horizontal axis with green shading, infection rate 10 percent or less; pink shading, infection rate between 10 and 25 percent.

**S** - Schools closed	**CTsd** - Child/Teen social distancing	**ASsd** - Adult/Senior social distancing	**Q** - Household Quantine	**T** - Antiviral Treatment	**P** - Antiviral Prophylaxis	**PEx** - Extended Antiviral Prophylaxis

ID Factor		None	ASsd	CTsd	CTsd, ASsd	S	S, ASsd	S, CTsd	S,CTsd, ASsd
0.75	None	28.3	27.0	26.9	24.4	9.8	8.8	7.7	6.6
	T	19.2	16.9	16.9	13.7	6.8	6.0	5.6	4.9
	Q	23.9	22.5	22.8	19.8	8.8	7.7	7.2	6.7
	P	14.0	11.9	12.0	9.8	6.2	5.5	5.4	4.7
	Q,T	14.9	12.9	13.1	10.8	6.4	5.7	5.4	5.0
	Q,P	11.2	9.6	10.5	8.8	5.7	5.4	5.2	4.8
	Pex	9.0	7.5	8.3	7.2	5.4	5.2	5.0	4.5
	Q,Pex	7.9	7.3	7.6	6.4	5.8	5.0	5.1	4.6
1.00	None	49.6	46.3	48.9	44.7	31.7	25.4	20.3	15.1
	T	42.3	38.8	40.9	36.3	22.9	17.5	14.4	10.1
	Q	45.2	42.9	45.1	41.9	27.2	22.0	20.2	14.9
	P	36.4	32.4	34.3	29.7	17.8	13.3	12.0	9.0
	Q,T	37.4	34.4	36.8	32.8	19.3	14.4	14.1	10.0
	Q,P	32.8	29.7	31.5	27.1	15.8	12.0	12.0	8.8
	Pex	24.9	21.5	23.6	19.6	14.2	11.3	10.7	8.2
	Q,Pex	23.1	19.3	21.8	17.8	12.9	9.8	10.6	8.2
1.25	None	62.7	57.8	61.7	56.9	51.4	45.1	38.6	30.4
	T	56.7	51.6	55.4	49.9	42.2	34.5	28.7	20.3
	Q	58.3	55.5	58.4	54.8	45.8	40.2	37.3	30.4
	P	50.1	45.5	48.8	43.6	34.4	27.2	23.6	16.9
	Q,T	51.9	48.3	51.3	47.1	36.9	30.6	27.8	20.9
	Q,P	46.5	42.9	45.7	41.2	31.2	24.9	23.2	16.9
	Pex	36.7	32.1	35.4	30.5	25.3	20.1	19.2	13.1
	Q,Pex	34.0	30.4	33.3	28.7	23.3	18.8	18.5	13.6
1.50	None	71.7	65.8	70.8	65.2	65.0	58.6	54.1	45.5
	T	66.5	60.1	65.4	59.2	57.1	50.0	44.2	34.4
	Q	67.8	64.4	67.6	63.7	59.8	54.5	52.4	45.4
	P	60.3	54.3	59.3	53.2	49.1	41.6	36.8	27.6
	Q,T	62.0	57.9	61.7	57.1	51.3	45.2	42.4	34.2
	Q,P	56.7	52.3	56.2	51.3	45.4	38.3	35.9	26.8
	Pex	45.5	40.2	44.6	38.9	35.1	30.3	27.7	21.7
	Q,Pex	43.0	38.7	42.5	37.4	33.2	27.7	26.6	20.9
2.00	None	82.7	76.1	81.7	75.8	80.2	74.8	73.7	65.9
	T	78.8	71.5	77.6	71.1	74.9	68.6	66.0	56.1
	Q	79.4	75.4	79.1	75.2	76.3	71.9	71.7	65.8
	P	73.6	66.4	72.4	65.6	68.0	60.8	57.9	47.7
	Q,T	75.0	70.2	74.4	69.9	70.1	64.5	64.0	56.3
	Q,P	70.0	65.0	69.8	64.4	64.1	57.7	56.8	47.7
	Pex	58.3	51.7	57.3	50.9	51.3	44.8	42.9	35.1
	Q,Pex	55.5	50.3	55.1	49.4	48.5	43.4	42.4	35.2
2.50	None	89.0	82.9	87.9	82.6	87.9	83.5	83.9	77.5
	T	86.1	78.7	84.9	78.4	84.5	78.9	78.6	70.0
	Q	86.4	82.4	85.9	82.3	84.9	81.5	82.3	77.4
	P	81.6	74.1	80.6	73.8	78.8	72.8	71.7	62.3
	Q,T	82.9	78.0	82.4	77.8	80.5	76.0	76.5	69.9
	Q,P	78.5	73.2	78.1	72.9	75.6	70.1	70.3	62.3
	Pex	67.1	59.8	66.1	59.0	62.1	55.7	54.9	46.9
	Q,Pex	64.2	58.5	63.9	57.9	59.3	53.8	53.6	46.4
3.00	None	92.7	87.2	91.9	87.2	92.3	88.8	89.6	84.3
	T	90.7	83.6	89.5	83.5	89.7	85.2	85.7	78.5
	Q	90.6	87.1	90.2	87.0	90.0	87.2	88.0	84.3
	P	87.0	79.7	86.0	79.5	85.5	80.0	80.4	72.0
	Q,T	87.9	83.2	87.2	83.2	86.6	82.9	83.9	78.6
	Q,P	84.3	79.2	83.8	78.9	82.5	78.0	78.8	72.1
	Pex	73.7	65.5	72.6	65.2	70.1	63.7	63.3	55.9
	Q,Pex	70.9	64.7	70.6	64.5	67.4	61.7	62.1	55.4

Extensions

Extensions to the core simulation matrix were explored through changes in the disease manifestation (Longini-like and Longini-like-extended), the contact network (similar transmission and augmented) and the availability of pre-pandemic vaccine. We also examined 3 pre-pandemic vaccination strategies based on 7-percent coverage of a 50-percent effective vaccine (at prevention of transmission) administered 1) randomly, 2) targeted to children and teens, or 3) targeted to adults. We compare these results with the core simulation matrix results below.

Longini-like Disease Manifestation

Once the disease infectivity (I_F) is calibrated in the model to yield an infection attack rate of approximately 50 percent for the unmitigated epidemic, total and age class-specific infection attack rates for the Ferguson-like and Longini-like disease manifestations are indistinguishable across the full range of I_F and for both 60 and 90 percent compliance (well within 1 standard deviation (*SD*) of each other). Maximum branching factors by age class and overall (an estimate of R_o, see [13]) are indistinguishable up to an I_F of 3.0 (an epidemic with infectivity twice that of 1918). At an I_F of 3.0 the maximum branching factor for the Longini manifestation falls below that of Ferguson by approximately 10 percent. Infectious contact fractions by age class and by transmission context also show no significant difference (nearly all within 1 *SD* of each other).

However, because the time scale of the Longini-like manifestation of epidemics is longer, all measures influenced by time scale are increased. For the unmitigated epidemic, generation time increases by approximately 40 percent (from 2.37 to 3.35 days), epidemic duration increases by 55 percent, total time of epidemic effects increases by 20 percent, time to peak infected increases by 36 percent, and time to peak symptomatics increases by 31 percent. Peak infected also increases slightly by 10 percent. Because of the 33-percent increase in the probability of becoming symptomatic if infected (*pS*) within the Longini-like disease manifestation, the total number of symptomatics is increased (34 percent) as well as their peak value (28 percent). These values for symptomatics translate directly into a 34 percent increase in deaths. The combination of the increase in *pS* and the longer time scales translates into a 61 percent greater number of days that adults are at home sick or tending sick children for the unmitigated epidemic.

Tables 20 through 23 show infection rates for the full set of mitigation interventions, at the range of I_F, 60 and 90-percent compliance, and regional vs. local-only mitigation. For network-based (social distancing) interventions, infection rates for the Longini-like disease manifestation are nearly identical to the Ferguson-like manifestation and those strategies within either green or pink zones do not change. However, for case-based interventions including antivirals, the Longini-like manifestation results in strategies having greater efficacy on infection rates (green and pink zones increase). This happens because the increased time scale of the epidemic allows antiviral prophylaxis to better capture the spreading disease. Over all mitigation strategies, the infection rate for the Ferguson-like manifestation is slightly higher; the average difference at 90-percent compliance is 1.5 percent (maximum, 11.6; minimum -6.9; *SD*, 2.2 percent). With decreasing compliance and local-only mitigation, this difference decreases somewhat, and the range and *SD* decrease by almost half.

While the symptomatic rate for the unmitigated epidemic is 33 percent greater for the Longini-like manifestation, the average difference between Ferguson-like and Longini-like across the 4 mitigation intervention combination tables is insignificant (between -3.9 and -6.3 with *SD* of 4.6 across both 60 and 90-percent compliance and regional vs. local-only mitigation). Most of these differences occur

for strategies that are ineffective. When effective mitigation strategies are implemented, the 2 manifestations produce nearly identical outcomes (differences in illness rates almost all below 1 percent).

Over all mitigation strategies, the Longini-like manifestation requires more antiviral courses, with an average difference at 90-percent compliance of -4.9 percent (maximum, 28.6; minimum, -52.1; *SD*, 12.6). These average differences do not change significantly with decreasing compliance and local-only mitigation. Once again, differences lessen significantly as mitigation strategies achieve an infection attack rate of 10 percent or less (green zone).

The average difference between adult days at home at 90 percent compliance for Ferguson-like and Longini-like manifestations is -3.6 (maximum, 4.6; minimum, -16.6; *SD*, 3.8). This increase in days adults are home for the Longini-like manifestation does not change significantly with decreasing compliance and contact with the external unmitigated epidemic. Unlike for illness attack rate and antiviral courses, these average differences also do not change significantly moving into the green zone.

Table 20: Longini-like disease manifestation for infection rates, regionally mitigated, 90-percent compliance

For Longini-like disease manifestation and implementation threshold when 10 cases are diagnosed. Case-based strategy combinations vertical, network-focused strategy combinations horizontal. Green shading, infection rates 10 percent or less; pink shading, infection rates between 10 and 25 percent.

S - Schools closed	CTsd - Child/Teen social distancing	ASsd - Adult/Senior social distancing	Q - Household Quantine	T - Antiviral Treatment	P - Antiviral Prophylaxis	PEx - Extended Antiviral Prophylaxis

ID Factor		None	ASsd	CTsd	CTsd,ASsd	S	S, ASsd	S,CTsd	,CTsd,ASs
0.75	None	28.1	19.1	11.1	6.8	2.5	2.0	1.3	1.3
	T	13.7	7.3	3.0	2.4	1.5	1.6	1.1	1.2
	Q	8.9	4.3	2.4	1.9	1.7	1.5	1.4	1.4
	P	4.3	2.4	1.7	1.5	1.2	1.2	0.0	1.1
	Q,T	3.5	2.7	1.8	1.5	1.3	1.3	1.1	1.0
	Q,P	2.4	2.3	1.4	1.6	1.4	1.2	1.1	0.0
	Pex	2.5	1.8	1.3	1.6	1.3	1.2	1.1	1.1
	Q,Pex	1.6	1.4	1.5	1.3	1.3	1.2	1.2	1.1
1.00	None	50.4	39.1	39.9	31.0	23.3	13.6	1.9	1.5
	T	40.1	29.3	25.6	16.2	6.4	3.4	1.5	1.4
	Q	34.2	25.7	16.8	11.2	5.1	3.7	1.5	1.6
	P	28.8	18.0	7.9	4.6	2.4	2.1	1.3	1.3
	Q,T	24.7	13.8	5.3	4.6	1.9	2.0	1.5	1.3
	Q,P	19.8	10.3	3.9	2.8	2.0	1.7	1.3	1.2
	Pex	13.1	6.8	3.3	2.9	1.9	1.5	1.4	1.2
	Q,Pex	6.1	3.9	2.2	2.0	1.6	1.5	1.5	1.4
1.25	None	63.2	50.2	55.0	44.4	47.7	37.3	5.5	2.6
	T	54.7	42.0	44.2	34.3	31.1	18.8	2.2	1.9
	Q	49.2	39.9	37.4	31.4	26.5	18.8	3.6	2.5
	P	43.3	32.3	28.9	20.4	10.1	5.4	1.7	1.5
	Q,T	40.4	30.8	23.6	15.7	6.9	5.3	1.7	1.7
	Q,P	36.3	25.8	14.9	9.9	4.4	2.7	1.7	1.5
	Pex	28.8	20.6	15.9	7.9	4.5	2.8	1.7	1.5
	Q,Pex	20.9	13.0	5.6	4.4	2.4	2.2	1.5	1.6
1.50	None	71.8	57.1	65.3	53.3	62.0	51.0	20.9	5.7
	T	64.6	49.8	56.1	44.4	49.4	37.4	4.1	2.2
	Q	58.7	49.2	49.7	43.4	43.8	36.4	9.7	5.2
	P	53.8	41.0	43.0	33.2	30.6	17.8	2.2	1.9
	Q,T	51.0	40.2	38.1	31.3	26.3	16.6	2.9	2.1
	Q,P	45.9	35.2	31.4	23.2	15.5	8.5	2.1	1.9
	Pex	38.2	29.1	28.4	19.9	13.5	7.3	2.0	1.6
	Q,Pex	32.2	23.5	16.5	10.3	5.8	4.3	2.0	1.7
2.00	None	82.5	66.1	77.6	64.2	78.1	67.7	55.5	29.3
	T	77.4	59.8	71.0	56.9	69.8	58.0	30.5	6.3
	Q	71.8	60.8	65.6	57.4	64.6	56.6	40.9	25.7
	P	67.3	51.7	59.5	47.2	55.7	43.2	8.6	3.5
	Q,T	64.8	53.0	56.7	47.2	53.0	43.2	13.1	5.3
	Q,P	59.5	47.0	49.4	39.8	44.8	33.2	5.4	3.2
	Pex	51.9	39.5	44.4	34.3	38.7	28.3	4.5	2.7
	Q,Pex	45.5	35.1	35.4	26.7	27.2	17.6	3.5	2.6
2.50	None	88.6	71.8	84.9	70.9	86.3	76.8	72.3	48.7
	T	84.8	66.0	79.9	64.3	81.0	69.6	56.1	21.1
	Q	79.9	68.1	75.1	65.8	75.8	68.3	60.6	45.9
	P	76.1	58.5	70.1	55.8	69.6	57.3	32.3	7.4
	Q,T	73.7	60.7	67.4	56.8	67.4	57.5	39.7	17.7
	Q,P	68.5	54.6	61.1	49.8	60.5	49.0	19.3	6.8
	Pex	61.0	46.8	55.1	42.9	52.3	41.0	16.1	4.8
	Q,Pex	54.9	42.6	47.6	36.9	43.9	33.0	9.0	4.6
3.00	None	92.4	76.1	89.4	75.5	90.9	82.5	81.7	60.6
	T	89.5	70.3	85.6	69.4	87.1	76.7	70.5	38.5
	Q	85.1	73.1	81.4	71.7	82.9	75.6	72.5	58.1
	P	82.2	63.5	77.4	61.7	78.1	66.5	52.5	15.7
	Q,T	79.9	66.3	75.2	63.7	75.9	66.8	57.4	35.2
	Q,P	74.9	60.0	69.4	56.3	70.0	59.1	41.9	13.9
	Pex	68.2	52.0	63.0	49.4	62.2	50.9	32.8	8.2
	Q,Pex	62.3	48.3	56.2	44.1	54.4	43.3	23.6	8.2

Table 21: Longini-like disease manifestation for infection rates, regionally mitigated, 60-percent compliance

For Longini-like disease manifestation and implementation threshold when 10 cases are diagnosed. Case-based strategy combinations vertical, network-focused strategy combinations horizontal. Green shading, infection rates 10 percent or less; pink shading, infection rates between 10 and 25 percent.

S - Schools closed	CTsd - Child/Teen social distancing	ASsd - Adult/Senior social distancing	Q - Household Quantine	T - Antiviral Treatment	P - Antiviral Prophylaxis	PEx - Extended Antiviral Prophylaxis

ID Factor		None	ASsd	CTsd	CTsd,ASsd	S	S, ASsd	S,CTsd	,CTsd,ASs
0.75	None	28.8	26.9	26.7	23.8	2.6	2.4	1.4	1.4
	T	13.2	11.5	8.9	7.6	1.6	1.7	1.2	1.5
	Q	22.7	21.1	20.5	17.5	2.0	1.8	1.5	1.4
	P	6.0	5.5	3.3	3.4	1.4	1.3	1.2	1.2
	Q,T	7.4	6.7	5.2	4.8	1.3	1.3	1.3	1.1
	Q,P	4.2	3.1	2.6	2.2	1.2	1.6	1.2	1.0
	Pex	2.1	2.2	1.8	2.1	1.6	1.2	1.2	0.0
	Q,Pex	1.9	1.6	1.5	1.6	1.3	1.1	1.1	1.3
1.00	None	50.1	47.2	49.0	45.2	26.2	20.7	6.4	4.4
	T	39.7	36.7	37.8	34.2	8.0	4.0	2.1	1.9
	Q	44.5	43.4	44.2	42.1	16.3	12.6	5.1	4.7
	P	30.8	28.0	27.1	23.6	2.4	2.2	1.5	1.5
	Q,T	33.4	31.5	31.8	29.3	3.7	2.8	2.0	2.0
	Q,P	26.6	22.8	21.4	18.1	2.4	2.1	1.5	1.6
	Pex	14.6	12.7	11.6	8.3	2.0	1.8	1.5	1.5
	Q,Pex	10.7	8.7	7.0	6.1	1.7	1.7	1.3	1.3
1.25	None	63.0	58.8	61.9	57.9	50.1	45.2	33.4	25.5
	T	54.1	50.1	52.7	48.6	34.5	28.4	8.6	5.3
	Q	57.9	56.3	57.6	55.8	42.0	38.4	30.1	25.5
	P	45.8	41.4	43.2	39.4	16.3	10.3	3.5	2.6
	Q,T	48.4	46.4	47.4	45.2	23.0	18.7	6.8	5.5
	Q,P	41.2	38.7	39.1	36.1	10.1	6.7	3.0	2.6
	Pex	30.0	27.5	28.3	24.9	5.4	4.2	2.3	2.0
	Q,Pex	26.8	24.6	23.8	21.3	3.7	3.2	2.4	1.8
1.50	None	71.8	66.9	70.7	66.3	64.4	59.5	53.3	45.9
	T	64.3	59.1	63.2	58.0	52.2	46.3	32.7	23.1
	Q	67.0	64.9	66.7	64.6	57.5	54.6	49.1	45.4
	P	55.9	51.1	53.9	49.6	38.0	31.7	13.7	7.1
	Q,T	58.5	56.5	58.0	55.3	43.5	39.2	29.1	22.8
	Q,P	51.4	48.3	50.0	46.9	30.1	24.8	10.4	7.3
	Pex	39.7	36.4	38.4	34.4	20.8	14.6	4.6	3.1
	Q,Pex	36.5	34.0	34.6	32.1	13.4	10.4	3.7	3.2
2.00	None	82.8	77.5	81.7	77.1	79.9	76.0	73.7	67.8
	T	77.4	70.9	75.8	70.3	72.3	67.2	61.7	52.9
	Q	78.7	76.5	78.5	76.3	75.1	72.6	70.6	67.5
	P	69.6	63.4	67.9	62.5	61.6	55.8	47.2	36.5
	Q,T	72.2	69.3	71.6	68.9	65.6	62.2	58.2	53.2
	Q,P	65.3	61.5	64.4	61.0	56.1	51.5	43.9	36.3
	Pex	53.1	48.1	51.8	47.1	43.5	38.0	27.9	18.3
	Q,Pex	49.8	46.4	48.9	45.3	38.3	34.7	24.8	18.2
2.50	None	89.1	83.9	88.0	83.8	87.8	84.5	83.9	79.2
	T	84.9	78.3	83.5	78.0	82.6	78.1	76.1	69.0
	Q	85.8	83.6	85.3	83.4	84.1	82.3	81.6	79.0
	P	78.2	71.5	76.9	71.1	74.7	69.0	65.2	56.2
	Q,T	80.5	77.4	79.9	77.3	77.3	74.5	73.1	68.6
	Q,P	74.4	70.4	73.7	70.0	69.6	65.4	62.2	56.0
	Pex	62.4	56.1	61.2	55.7	57.0	51.3	45.8	37.3
	Q,Pex	59.0	54.9	58.4	54.2	52.7	47.7	43.5	37.7
3.00	None	92.8	88.3	92.0	88.2	92.1	89.5	89.7	85.7
	T	89.7	83.5	88.5	83.4	88.5	84.7	84.3	78.3
	Q	90.2	88.0	89.8	88.0	89.3	87.9	87.7	85.7
	P	84.1	77.4	82.8	77.2	82.1	77.4	75.9	68.3
	Q,T	86.0	82.9	85.5	82.8	84.3	82.0	81.4	78.0
	Q,P	80.5	76.6	80.0	76.4	78.1	74.9	73.4	67.9
	Pex	69.3	62.5	68.4	62.1	66.0	60.6	57.8	49.3
	Q,Pex	66.2	61.5	65.7	61.0	62.2	57.8	55.2	49.5

Table 22: Longini-like disease manifestation for infection rates, local-only mitigation, 90-percent compliance

For Longini-like disease manifestation and implementation threshold when 10 cases are diagnosed. Case-based strategy combinations vertical, network-focused strategy combinations horizontal. Green shading, infection rates 10 percent or less; pink shading, infection rates between 10 and 25 percent.

S - Schools closed	CTsd - Child/Teen social distancing	ASsd - Adult/Senior social distancing	Q - Household Quantine	T - Antiviral Treatment	P - Antiviral Prophylaxis	PEx - Extended Antiviral Prophylaxis

ID Factor		None	ASsd	CTsd	CTsd,ASsc	S	S, ASsd	S,CTsd	,CTsd,ASs
0.75	None	28.7	20.4	15.6	9.9	6.3	4.2	3.4	2.5
	T	17.3	9.4	7.8	4.5	3.9	2.6	2.5	2.0
	Q	13.5	7.6	6.9	4.3	4.7	3.2	3.3	2.6
	P	8.8	4.2	4.8	3.0	3.2	2.4	2.4	2.0
	Q,T	7.9	4.2	4.7	3.1	3.4	2.5	2.5	2.1
	Q,P	6.3	3.6	3.6	2.6	3.0	2.2	2.4	1.9
	Pex	5.4	3.3	4.1	2.6	3.0	2.2	2.4	2.0
	Q,Pex	4.3	2.7	3.4	2.5	2.7	2.2	2.4	1.9
1.00	None	49.9	39.2	40.2	31.6	28.4	18.8	9.5	4.7
	T	41.1	30.8	29.7	21.2	16.9	9.4	6.7	3.5
	Q	36.7	27.4	25.6	17.7	16.8	10.0	8.7	4.8
	P	31.0	21.8	18.8	11.2	10.7	5.7	5.6	3.2
	Q,T	28.8	18.0	16.9	9.8	10.4	5.7	6.3	3.5
	Q,P	24.9	15.4	13.4	7.2	8.6	4.7	5.5	3.1
	Pex	19.5	12.2	12.6	7.3	8.0	4.6	5.2	3.0
	Q,Pex	15.2	8.2	9.9	5.2	7.2	4.1	5.2	3.0
1.25	None	62.6	50.2	55.1	45.2	49.0	38.5	20.3	9.8
	T	55.3	42.9	46.0	36.1	36.4	25.2	13.3	6.2
	Q	51.0	41.5	42.1	33.7	34.6	25.3	18.2	9.5
	P	45.5	34.4	34.9	25.6	23.8	13.7	10.5	5.1
	Q,T	43.1	33.4	32.3	23.9	24.0	14.4	12.3	6.4
	Q,P	39.0	28.5	26.9	18.1	18.8	10.3	10.2	5.2
	Pex	31.5	23.2	23.7	15.8	16.0	9.4	9.2	4.8
	Q,Pex	26.6	17.9	18.6	11.3	13.7	7.8	8.9	4.6
1.50	None	71.4	57.1	65.0	53.7	62.6	51.7	34.3	17.6
	T	64.9	50.7	57.5	46.1	52.2	40.2	22.7	10.4
	Q	60.8	50.6	53.5	45.2	49.5	40.0	30.4	17.3
	P	55.4	43.1	46.7	36.3	38.5	26.5	17.0	8.2
	Q,T	53.5	42.6	44.7	35.5	38.2	26.6	20.2	10.3
	Q,P	48.9	37.7	38.8	28.4	31.1	19.7	16.3	8.1
	Pex	41.3	31.5	33.6	24.7	25.8	17.0	13.7	7.2
	Q,Pex	36.1	26.1	27.7	18.5	21.5	13.5	13.4	7.2
2.00	None	82.3	66.2	77.3	64.3	77.9	67.8	58.5	36.8
	T	77.4	60.7	71.5	58.0	71.2	59.1	44.2	22.5
	Q	73.5	62.3	68.7	59.1	67.6	58.6	52.4	36.9
	P	68.9	54.0	62.5	50.2	59.5	47.1	32.8	15.9
	Q,T	67.1	55.3	61.4	50.6	58.4	47.5	39.2	22.1
	Q,P	62.1	49.7	55.1	43.9	51.5	39.9	30.7	15.9
	Pex	54.8	42.4	48.7	37.8	42.9	32.7	24.3	12.7
	Q,Pex	49.4	38.2	42.5	32.0	37.1	26.5	23.2	12.7
2.50	None	88.3	71.8	84.4	70.8	86.0	76.5	72.8	51.8
	T	84.8	66.7	79.9	65.2	81.2	70.1	61.2	35.9
	Q	81.2	69.4	77.4	67.5	78.0	69.4	66.9	51.8
	P	77.4	60.8	72.1	58.6	71.5	59.7	48.2	25.1
	Q,T	75.6	62.9	71.2	60.0	70.6	60.3	54.9	35.6
	Q,P	70.7	57.4	65.5	53.5	64.4	53.0	45.0	25.2
	Pex	64.0	49.6	58.6	46.6	55.2	44.5	34.4	18.8
	Q,Pex	58.5	46.3	53.0	41.3	48.9	38.3	32.7	19.2
3.00	None	92.1	75.9	89.1	75.2	90.9	82.2	81.7	62.2
	T	89.4	71.1	85.5	70.2	87.3	77.2	72.8	47.6
	Q	86.4	74.4	83.3	73.2	84.2	76.5	76.5	61.9
	P	83.1	65.8	79.0	64.3	79.5	68.4	60.9	35.1
	Q,T	81.5	68.5	77.9	66.5	78.2	68.9	66.6	47.6
	Q,P	77.0	63.2	72.9	60.3	73.1	62.1	56.6	35.3
	Pex	70.8	55.4	66.1	52.9	64.2	52.8	44.4	25.6
	Q,Pex	65.4	52.1	60.8	48.5	58.1	47.5	41.7	26.0

Table 23: Longini-like disease manifestation for infection rates, local-only mitigation, 60-percent compliance

For Longini-like disease manifestation and implementation threshold when 10 cases are diagnosed. Case-based strategy combinations vertical, network-focused strategy combinations horizontal. Green shading, infection rates 10 percent or less; pink shading, infection rates between 10 and 25 percent.

S - Schools closed	CTsd - Child/Teen social distancing	ASsd - Adult/Senior social distancing	Q - Household Quantine	T - Antiviral Treatment	P - Antiviral Prophylaxis	PEx - Extended Antiviral Prophylaxis

ID Factor		None	ASsd	CTsd	CTsd,ASsd	S	S, ASsd	S,CTsd	,CTsd,ASs
0.75	None	28.9	26.5	26.8	23.5	6.9	4.6	4.5	3.3
	T	16.8	13.5	14.8	10.6	4.2	2.9	3.3	2.4
	Q	23.5	22.1	22.2	19.3	5.7	3.8	4.4	3.2
	P	10.1	7.5	8.9	5.6	3.5	2.6	3.0	2.2
	Q,T	11.8	8.9	10.7	7.5	3.9	2.8	3.2	2.4
	Q,P	8.1	5.7	6.5	4.4	3.4	2.5	2.8	2.2
	Pex	5.6	4.0	5.4	3.6	3.1	2.5	2.8	2.1
	Q,Pex	4.9	3.5	4.8	3.1	3.1	2.4	2.8	2.2
1.00	None	49.5	45.8	48.4	44.7	29.4	23.4	17.2	11.3
	T	40.5	36.8	38.8	34.6	17.6	12.4	10.5	6.5
	Q	44.9	43.0	44.6	41.6	23.4	18.5	16.4	10.9
	P	32.8	29.3	30.3	25.5	12.5	8.0	8.3	5.0
	Q,T	35.3	32.3	34.1	30.5	14.6	9.4	10.2	6.3
	Q,P	29.4	25.4	26.9	22.1	10.9	7.0	8.4	5.1
	Pex	20.9	17.1	18.7	14.8	9.5	6.1	7.0	4.5
	Q,Pex	18.1	14.4	16.5	12.2	8.5	5.7	7.0	4.3
1.25	None	62.6	57.7	61.1	57.0	49.8	44.2	37.1	28.8
	T	54.8	50.2	53.5	48.3	38.3	31.4	24.4	16.3
	Q	58.1	55.3	57.8	54.8	44.1	38.4	35.6	28.6
	P	47.4	42.6	45.5	40.5	28.1	20.6	17.7	11.0
	Q,T	49.9	46.5	49.3	45.6	31.9	25.3	23.5	15.9
	Q,P	43.7	39.9	42.2	37.7	24.7	17.5	17.0	10.8
	Pex	33.6	29.4	31.9	27.1	19.0	13.3	13.5	8.4
	Q,Pex	30.7	26.5	29.2	24.6	17.0	11.9	13.2	8.7
1.50	None	71.1	65.8	70.0	65.2	63.7	58.2	53.5	45.4
	T	64.7	58.9	63.3	57.7	54.1	47.0	40.4	30.6
	Q	67.0	64.1	66.9	63.6	58.1	53.4	51.5	45.4
	P	57.5	51.9	55.9	50.2	43.4	36.0	30.2	21.0
	Q,T	59.9	56.2	59.6	55.5	47.3	41.2	38.8	30.5
	Q,P	53.9	49.4	52.9	48.1	38.9	31.2	29.5	20.4
	Pex	43.0	38.1	41.8	36.2	29.8	23.1	21.3	14.4
	Q,Pex	40.0	35.9	39.2	33.9	27.1	20.4	21.2	14.4
2.00	None	82.2	76.2	81.0	75.9	79.4	74.6	73.2	66.2
	T	77.4	70.5	76.0	69.8	72.7	66.7	63.8	54.3
	Q	78.7	75.4	78.4	75.2	75.1	71.1	71.1	66.2
	P	70.8	64.0	69.6	63.4	64.1	57.0	53.3	42.4
	Q,T	73.1	69.0	72.7	68.6	67.2	62.2	61.5	54.3
	Q,P	67.3	62.5	66.7	61.7	59.7	52.9	51.7	42.1
	Pex	56.4	50.0	55.2	48.9	47.6	40.9	37.4	28.6
	Q,Pex	53.4	48.5	52.8	47.2	43.8	37.4	36.9	28.5
2.50	None	88.4	82.8	87.4	82.6	87.3	83.3	83.4	77.6
	T	84.8	77.7	83.6	77.4	82.8	77.5	76.6	68.8
	Q	85.7	82.4	85.3	82.2	83.9	80.9	81.6	77.6
	P	79.2	71.9	78.1	71.6	75.6	69.4	67.7	58.2
	Q,T	81.1	77.0	80.7	76.8	78.2	74.0	74.2	68.5
	Q,P	75.9	71.0	75.4	70.7	71.6	66.1	66.2	58.1
	Pex	65.2	58.2	64.3	57.4	59.3	52.7	50.4	41.2
	Q,Pex	62.3	56.8	61.9	55.9	55.9	49.8	49.0	41.2
3.00	None	92.3	87.3	91.5	87.3	91.8	88.4	89.3	84.5
	T	89.6	82.9	88.4	82.8	88.5	84.0	84.4	77.5
	Q	90.1	87.1	89.6	87.1	89.3	86.9	87.7	84.4
	P	84.8	77.7	83.7	77.5	82.8	77.5	77.3	68.9
	Q,T	86.4	82.5	85.9	82.3	84.8	81.2	82.2	77.6
	Q,P	81.9	77.1	81.5	76.8	79.4	74.8	75.6	68.9
	Pex	71.9	64.2	70.9	63.8	67.9	61.5	60.0	51.1
	Q,Pex	69.0	63.1	68.8	62.9	64.5	58.9	58.9	51.2

Longini-like Disease Manifestation with Extended Duration of Infectiousness

It is possible that the period of viral shedding could be increased in a pandemic caused by a novel influenza virus [26, 27, 33]. We examined an extension of the duration of infectiousness through the 7-day recovery period. Compared to the Longini manifestation across the range of I_F, infection rates in epidemics without mitigation strategies were very similar (population infection rates ranged from 28% [I_F .75] to 92% [I_F 3.0] with the original Longini manifestation; 29% [I_F .75] to 92% [I_F 3.0] with Longini extended infectiousness). However the extended period of infectiousness leads to a marked increase in epidemic duration from 150 to 282 days [I_F .75] and 42 to 92 days [I_F 3.0].

Mitigation strategies are moderately impeded by the extended period of infectiousness. For the best strategy (P+S+CTsd+ASsd; 90% compliance, regionally mitigated), an I_F 1.5 epidemic infects 3% of population vs. 2% for the original Longini manifestation and with an increase from 2% to 3% in the level of coverage with antivirals. With no antivirals (Q+S+CTsd+ASsd, 90% compliance, regionally mitigated) 16% of the population is infected vs. 5% for the original Longini manifestation. If only antivirals are used, Pex (90% compliance, regionally mitigated) requires 208% population coverage vs. 192% for the original Longini manifestation. However, the P strategy requires nearly identical coverage: 63% for the extended period of infectiousness vs. 64% for the original Longini manifestation at I_F 1.5.

Average adult days at home reflect the increased duration of epidemics seen with the extended period of infectiousness. For an I_F 1.5 epidemic with best mitigation strategies applied (P+S+CTsd+ASsd; 90% compliance, regionally mitigated) adults spend 24 days at home (vs. 10 for original Longini manifestation and 6 for Ferguson-like). Required adult days at home more than double when compliance drops to 60% and increase nearly 50% when mitigation is only locally applied.

Tables 24 through 27 show infection rates for the full set of intervention combinations, at the range of I_F, 60 and 90-percent compliance, and regional vs. local-only mitigation. In these tables, interventions combinations that accomplish infection rates of less than or equal to 10 percent and between 10 and 25 percent have been colored green and pink, respectively.

Table 24: Longini extended infectiousness, for infection rates, regional mitigation, 90-percent compliance

For Longini-like disease manifestation and implementation threshold when 10 cases are diagnosed. Case-based strategy combinations vertical, network-focused strategy combinations horizontal. Green shading, infection rates 10 percent or less; pink shading, infection rates between 10 and 25 percent.

**S** - Schools closed	**CTsd** - Child/Teen social distancing	**ASsd** - Adult/Senior social distancing	**Q** - Household Quantine	**T** - Antiviral Treatment	**P** - Antiviral Prophylaxis	**PEx** - Extended Antiviral Prophylaxis

ID Factor		None	ASsd	CTsd	CTsd, ASsd	S	S, ASsd	S, CTsd	S,CTsd, ASsd
0.75	None	28.5	17.3	11.0	7.7	3.5	3.1	1.8	1.6
	T	9.1	4.8	2.7	2.6	1.8	1.6	1.2	1.2
	Q	10.5	5.0	3.6	2.6	2.1	2.1	1.7	1.5
	P	3.0	2.0	1.7	1.4	1.5	1.4	1.4	1.3
	Q,T	4.3	1.8	1.6	1.5	1.7	1.5	1.4	1.2
	Q,P	2.0	2.0	1.4	1.7	1.3	1.2	1.1	1.2
	Pex	1.7	1.4	1.4	1.5	1.4	1.5	1.2	1.4
	Q,Pex	1.7	1.5	1.4	1.4	1.4	1.4	1.2	1.3
1.00	None	51.0	38.9	40.1	31.0	28.4	20.0	3.5	2.4
	T	36.6	24.8	18.8	10.6	5.3	3.8	1.8	1.7
	Q	37.7	27.0	21.5	14.5	10.3	7.2	3.1	2.7
	P	19.8	9.7	4.5	2.9	2.3	1.9	1.6	1.4
	Q,T	22.5	9.9	5.5	3.6	2.3	2.3	1.6	1.8
	Q,P	11.5	5.4	2.4	2.3	2.0	1.9	1.7	1.4
	Pex	5.6	4.1	2.8	2.1	1.9	2.0	1.6	1.6
	Q,Pex	3.4	2.7	1.9	1.9	1.6	1.7	1.3	1.6
1.25	None	63.7	50.3	55.2	45.2	50.1	41.2	13.8	6.7
	T	52.7	38.9	39.8	29.9	27.7	17.9	3.0	2.5
	Q	52.2	41.6	40.7	33.6	34.2	27.3	7.9	5.2
	P	39.3	25.9	18.2	11.7	6.2	4.8	1.9	1.6
	Q,T	40.1	27.0	19.1	14.2	8.2	6.5	2.3	2.2
	Q,P	31.6	16.6	8.3	6.0	4.2	3.1	2.2	1.9
	Pex	20.0	11.5	6.1	4.3	3.9	2.7	2.1	2.0
	Q,Pex	13.3	7.3	3.5	3.1	2.8	2.1	1.9	1.7
1.50	None	72.2	57.7	65.2	54.4	63.1	53.7	35.8	18.6
	T	63.1	47.9	53.0	41.4	46.7	36.3	7.1	3.3
	Q	61.7	51.0	53.2	45.6	49.4	42.5	25.0	15.9
	P	50.1	36.3	35.2	25.7	21.4	12.0	3.0	2.6
	Q,T	51.3	38.3	37.3	29.3	27.8	18.9	3.9	3.1
	Q,P	43.9	29.4	23.9	15.3	12.1	8.0	2.9	2.5
	Pex	32.7	22.5	18.1	11.7	7.3	5.3	2.5	2.1
	Q,Pex	25.8	13.6	8.5	6.4	5.4	4.4	2.7	2.3
2.00	None	82.7	67.6	77.7	65.8	78.2	69.2	62.4	44.5
	T	75.9	58.9	68.7	55.2	67.8	56.6	35.2	12.9
	Q	73.9	62.6	68.2	60.0	67.8	60.4	53.1	42.6
	P	64.5	48.6	54.4	42.5	49.5	38.1	8.9	5.2
	Q,T	65.5	51.9	56.5	46.7	53.4	43.8	22.4	12.0
	Q,P	58.8	44.3	45.9	35.4	40.3	29.7	7.8	5.7
	Pex	47.6	35.5	37.7	28.2	30.6	21.6	4.5	4.0
	Q,Pex	41.5	30.3	28.6	20.0	19.7	12.8	5.1	4.2
2.50	None	88.5	73.6	84.8	72.7	86.3	78.0	76.0	59.7
	T	83.5	65.7	78.1	63.6	79.1	68.4	58.7	33.6
	Q	81.1	70.2	76.8	68.6	77.7	70.8	68.1	57.9
	P	73.7	56.5	66.1	52.6	64.8	53.0	30.6	12.4
	Q,T	74.2	60.6	67.4	57.0	67.1	58.0	46.5	31.6
	Q,P	68.4	52.7	58.6	46.9	56.9	45.5	23.3	11.0
	Pex	56.9	43.4	49.9	38.3	46.5	36.3	13.7	7.3
	Q,Pex	51.7	38.6	42.3	32.4	38.0	28.7	11.8	6.6
3.00	None	92.1	78.2	89.3	77.5	90.7	83.3	83.9	69.1
	T	88.4	70.7	84.3	69.4	85.5	75.8	71.6	48.5
	Q	86.0	75.4	82.6	74.4	83.9	77.4	77.2	67.5
	P	79.9	61.8	73.8	59.3	74.4	62.6	49.4	25.2
	Q,T	80.3	66.5	75.1	64.3	75.7	66.7	61.6	46.4
	Q,P	74.8	58.6	67.5	54.8	67.7	56.2	42.6	23.3
	Pex	64.1	48.8	58.2	45.1	57.0	46.0	29.3	11.4
	Q,Pex	59.1	45.4	51.8	40.4	49.3	39.1	23.5	10.7

Table 25: Longini extended infectiousness, for infection rates, regional mitigation, 60-percent compliance

For Longini-like disease manifestation and implementation threshold when 10 cases are diagnosed. Case-based strategy combinations vertical, network-focused strategy combinations horizontal. Green shading, infection rates 10 percent or less; pink shading, infection rates between 10 and 25 percent.

S - Schools closed	CTsd - Child/Teen social distancing	ASsd - Adult/Senior social distancing	Q - Household Quantine	T - Antiviral Treatment	P - Antiviral Prophylaxis	PEx - Extended Antiviral Prophylaxis

ID Factor		None	ASsd	CTsd	CTsd, ASsd	S	S, ASsd	S, CTsd	S,CTsd, ASsd
0.75	None	30.1	26.8	27.0	25.7	3.5	2.9	2.0	2.0
	T	9.3	7.8	5.6	5.5	1.9	1.6	1.3	1.5
	Q	25.2	24.3	23.4	22.3	2.3	2.5	1.8	2.0
	P	4.6	3.9	3.3	2.6	1.5	1.5	1.2	1.4
	Q,T	6.6	5.3	4.4	4.1	1.7	1.5	1.3	1.3
	Q,P	3.3	3.6	2.7	2.4	1.5	1.5	1.5	1.3
	Pex	1.8	2.1	1.6	1.9	1.4	1.4	1.2	1.8
	Q,Pex	2.1	1.6	1.8	1.6	1.4	1.6	1.5	1.3
1.00	None	50.6	47.6	49.5	46.7	26.1	23.1	12.0	8.8
	T	37.0	34.5	34.6	31.3	5.4	4.8	2.5	2.4
	Q	47.0	45.5	46.4	44.3	19.9	17.2	10.5	8.4
	P	27.1	24.2	22.6	18.9	3.1	2.9	2.1	2.0
	Q,T	32.6	31.1	30.0	28.0	4.0	4.0	2.5	2.6
	Q,P	23.5	19.1	16.4	15.6	2.5	2.3	1.8	1.9
	Pex	9.9	7.9	7.3	5.6	2.0	1.8	1.9	1.8
	Q,Pex	7.4	6.2	4.8	4.9	2.0	2.0	1.7	1.6
1.25	None	63.6	59.9	62.5	59.3	50.4	46.6	39.1	34.2
	T	52.4	48.6	50.6	46.9	29.4	25.6	11.9	7.3
	Q	60.1	58.2	60.2	57.9	45.4	43.0	37.5	33.8
	P	43.4	39.2	40.7	37.0	11.7	9.3	4.2	3.9
	Q,T	48.5	45.9	46.9	45.0	23.0	18.4	9.3	7.5
	Q,P	40.3	37.0	37.1	35.4	7.9	7.0	4.0	4.0
	Pex	26.7	24.0	24.4	21.5	5.2	4.3	2.8	3.0
	Q,Pex	23.3	21.1	21.2	18.0	4.5	3.8	2.8	2.4
1.50	None	72.1	68.2	71.3	67.7	64.4	60.9	56.8	51.7
	T	62.9	58.1	61.4	57.2	48.9	44.5	34.7	28.0
	Q	69.1	67.2	68.7	66.9	60.1	57.6	54.9	51.7
	P	54.2	49.6	51.6	47.7	32.5	27.3	14.3	9.2
	Q,T	59.0	56.3	58.1	55.5	42.9	39.5	31.8	28.2
	Q,P	51.4	47.6	49.0	46.3	27.8	23.6	13.8	10.5
	Pex	37.5	33.8	35.4	32.3	14.7	11.4	5.9	5.0
	Q,Pex	35.2	32.0	33.4	30.1	11.2	9.7	5.5	4.8
2.00	None	83.0	78.8	82.1	78.7	80.0	76.8	75.5	71.6
	T	76.0	70.6	74.8	70.3	69.8	65.8	62.1	55.8
	Q	80.4	78.2	80.1	78.2	76.7	74.7	73.9	71.5
	P	68.0	62.9	66.4	61.9	58.2	53.0	46.9	39.8
	Q,T	72.5	69.5	71.9	69.2	65.1	62.1	59.7	55.6
	Q,P	65.5	61.6	64.3	60.6	54.5	49.9	44.9	39.2
	Pex	51.6	46.8	50.2	46.2	39.5	35.5	27.7	20.6
	Q,Pex	49.3	46.1	48.0	45.0	36.1	32.2	25.8	21.6
2.50	None	89.1	85.3	88.3	85.2	87.8	85.2	85.1	81.8
	T	84.0	78.7	82.7	78.3	80.8	77.3	76.0	71.0
	Q	86.8	84.9	86.4	84.9	85.3	83.7	83.6	81.6
	P	77.0	71.2	75.5	70.8	71.8	67.3	64.7	57.9
	Q,T	80.8	77.9	80.2	77.7	77.0	74.7	73.7	70.6
	Q,P	74.7	70.6	73.4	70.2	68.6	65.0	62.8	57.8
	Pex	61.1	55.9	60.2	55.2	54.9	49.8	46.1	40.1
	Q,Pex	59.0	55.1	58.2	54.5	51.4	47.7	44.5	39.7
3.00	None	92.7	89.5	92.1	89.5	92.0	90.1	90.3	87.7
	T	88.8	83.9	87.8	83.8	87.3	84.1	83.8	79.4
	Q	90.9	89.4	90.6	89.2	90.1	88.8	89.0	87.6
	P	82.9	77.2	81.6	77.0	80.0	75.9	75.0	69.6
	Q,T	86.1	83.4	85.6	83.4	84.3	82.2	82.0	79.7
	Q,P	80.6	76.9	80.0	76.7	77.4	74.2	73.2	69.1
	Pex	68.1	62.4	66.9	62.1	63.8	59.6	58.0	51.5
	Q,Pex	66.0	61.7	65.3	61.5	61.3	57.7	56.2	51.3

Table 26: Longini extended infectiousness, for infection rates, local-only mitigation, 90-percent compliance

For Longini-like disease manifestation and implementation threshold when 10 cases are diagnosed. Case-based strategy combinations vertical, network-focused strategy combinations horizontal. Green shading, infection rates 10 percent or less; pink shading, infection rates between 10 and 25 percent.

**S** - Schools closed	**CTsd** - Child/Teen social distancing	**ASsd** - Adult/Senior social distancing	**Q** - Household Quantine	**T** - Antiviral Treatment	**P** - Antiviral Prophylaxis	**PEx** - Extended Antiviral Prophylaxis

ID Factor		None	ASsd	CTsd	CTsd,ASsd	S	S, ASsd	S,CTsd	,CTsd,ASs
0.75	None	29.8	19.6	17.5	11.4	9.3	5.6	4.6	3.3
	T	14.8	7.3	7.7	4.6	4.6	3.3	3.2	2.5
	Q	17.5	8.9	9.8	6.1	6.5	4.7	4.5	3.3
	P	7.2	4.0	5.0	3.2	3.7	2.8	2.9	2.5
	Q,T	8.7	4.4	5.8	3.4	4.0	2.9	3.2	2.4
	Q,P	6.0	3.6	4.4	3.0	3.5	2.8	2.9	2.6
	Pex	5.3	3.1	4.2	2.9	3.5	2.8	2.9	2.5
	Q,Pex	4.5	3.2	3.9	2.7	3.3	2.8	2.9	2.5
1.00	None	51.4	39.7	41.8	32.8	33.7	24.3	14.7	8.0
	T	39.7	28.4	28.3	18.6	19.5	11.3	9.0	4.9
	Q	40.8	30.2	30.8	22.0	23.7	15.3	13.9	8.0
	P	28.5	16.3	17.0	9.6	11.7	6.4	7.4	4.2
	Q,T	29.1	17.7	19.2	10.8	13.4	7.7	8.8	5.0
	Q,P	23.1	11.8	14.4	7.7	10.1	5.7	7.2	4.2
	Pex	17.6	8.9	12.5	7.0	9.3	5.4	6.7	4.0
	Q,Pex	14.3	7.8	11.0	6.0	8.5	5.0	6.8	4.1
1.25	None	64.3	51.0	56.8	46.4	52.4	42.5	29.9	16.7
	T	54.9	41.0	45.2	34.5	37.7	26.3	17.6	8.8
	Q	55.1	43.2	47.2	38.0	41.9	32.5	27.4	16.5
	P	43.6	30.0	32.2	21.1	24.0	14.3	13.0	6.9
	Q,T	44.8	31.6	34.8	24.0	27.9	17.7	16.5	8.7
	Q,P	38.4	25.2	27.0	16.6	20.8	11.6	12.8	6.9
	Pex	29.4	19.4	22.3	13.8	16.9	10.0	11.3	6.1
	Q,Pex	25.7	15.5	19.4	11.1	15.2	8.9	11.2	6.3
1.50	None	72.8	58.6	66.4	55.4	64.9	54.6	44.9	28.5
	T	64.6	50.0	57.0	44.8	52.2	40.3	28.5	14.5
	Q	64.4	52.8	58.5	48.6	55.2	46.2	41.3	28.4
	P	54.4	40.3	44.7	32.8	37.2	24.2	20.6	10.6
	Q,T	55.7	42.2	47.1	35.8	41.3	29.4	26.3	14.4
	Q,P	49.6	35.1	38.7	26.8	32.7	20.7	19.9	10.4
	Pex	39.6	28.0	32.3	21.5	25.8	16.7	17.0	9.3
	Q,Pex	35.4	23.6	27.9	17.9	22.9	14.3	16.2	9.1
2.00	None	83.0	68.2	78.5	66.3	79.3	69.6	66.1	48.8
	T	77.5	61.0	71.4	58.0	70.6	59.1	50.3	29.8
	Q	76.3	64.5	72.4	62.4	71.4	62.8	61.8	48.9
	P	68.3	52.5	61.3	47.9	57.6	44.9	37.3	20.4
	Q,T	69.5	55.6	63.6	51.8	60.7	49.5	46.5	30.0
	Q,P	63.8	48.7	55.8	43.1	51.9	39.1	35.7	20.3
	Pex	53.5	40.3	47.2	34.8	41.6	31.2	27.9	16.0
	Q,Pex	49.1	36.2	42.8	30.9	38.0	27.2	27.2	15.9
2.50	None	88.9	74.3	85.5	73.3	86.8	78.1	78.0	62.2
	T	84.8	67.9	80.0	66.1	80.8	70.1	65.9	45.0
	Q	83.3	71.9	80.2	70.7	80.4	72.4	74.0	61.9
	P	76.9	60.6	71.3	57.5	70.0	57.7	51.8	31.4
	Q,T	77.6	64.1	73.3	61.5	72.4	62.1	61.4	44.8
	Q,P	72.6	57.4	66.7	53.5	64.8	52.6	49.8	31.6
	Pex	62.6	48.4	57.3	44.9	53.8	42.5	38.7	23.8
	Q,Pex	58.6	45.3	53.3	40.5	49.3	38.2	37.4	23.6
3.00	None	92.4	78.5	89.8	78.1	91.2	83.5	85.0	70.6
	T	89.3	72.5	85.7	71.6	86.9	77.0	76.0	56.4
	Q	87.8	77.2	85.6	76.3	86.2	78.8	81.5	70.8
	P	82.7	66.1	78.4	64.1	78.0	66.5	63.6	42.0
	Q,T	83.2	70.0	79.8	68.4	79.7	70.2	71.7	56.0
	Q,P	78.5	63.7	74.2	61.0	73.5	61.7	61.0	42.1
	Pex	69.7	54.4	65.1	51.7	62.7	51.6	47.9	31.3
	Q,Pex	65.7	51.4	60.9	47.8	58.0	46.8	46.1	31.3

Table 27: Longini extended infectiousness, for infection rates, local-only mitigation, 60 percent compliance

For Longini-like disease manifestation and implementation threshold when 10 cases are diagnosed. Case-based strategy combinations vertical, network-focused strategy combinations horizontal. Green shading, infection rates 10 percent or less; pink shading, infection rates between 10 and 25 percent.

S - Schools closed	CTsd - Child/Teen social distancing	ASsd - Adult/Senior social distancing	Q - Household Quantine	T - Antiviral Treatment	P - Antiviral Prophylaxis	PEx - Extended Antiviral Prophylaxis

ID Factor		None	ASsd	CTsd	CTsd, ASsd	S	S, ASsd	S, CTsd	S,CTsd, ASsd
0.75	None	29.4	26.9	27.9	25.1	8.4	6.1	5.9	4.3
	T	15.6	11.8	13.4	9.6	5.0	3.5	3.8	3.0
	Q	26.2	24.6	25.2	22.4	7.1	5.2	5.8	4.1
	P	9.3	6.3	7.7	5.1	4.0	3.1	3.5	2.7
	Q,T	12.8	9.4	11.6	7.7	4.5	3.1	3.8	3.0
	Q,P	8.2	5.6	7.2	5.0	3.8	3.0	3.5	2.7
	Pex	5.8	4.1	5.6	3.6	3.6	2.9	3.4	2.7
	Q,Pex	5.4	3.8	5.0	3.7	3.5	3.0	3.1	2.7
1.00	None	51.0	47.3	49.7	45.8	32.0	26.3	23.1	16.9
	T	39.7	35.4	38.0	33.1	19.2	12.8	13.3	8.7
	Q	48.3	44.8	47.6	44.0	28.3	22.6	22.6	16.6
	P	31.8	26.3	28.8	24.0	13.8	8.7	10.4	6.5
	Q,T	36.5	31.8	35.2	30.2	16.7	11.3	13.0	8.4
	Q,P	29.8	24.3	26.8	21.9	12.7	8.0	10.4	6.6
	Pex	20.0	15.1	18.7	12.8	10.6	6.7	9.0	5.8
	Q,Pex	18.5	13.3	17.2	12.0	10.0	6.6	8.8	5.7
1.25	None	63.7	59.0	62.7	58.2	51.8	46.0	43.2	35.8
	T	54.6	49.3	53.2	48.0	38.1	30.4	28.1	19.8
	Q	61.1	57.5	60.5	57.0	47.8	42.6	42.2	35.5
	P	47.1	41.2	44.8	39.1	28.6	20.6	21.0	13.5
	Q,T	51.3	46.6	50.5	45.9	34.0	26.8	27.4	19.9
	Q,P	44.6	39.0	42.6	37.3	26.0	18.5	21.0	13.5
	Pex	33.4	27.7	31.9	25.6	20.2	14.0	16.2	10.7
	Q,Pex	31.4	25.5	29.8	23.7	18.7	13.0	16.2	10.6
1.50	None	72.3	67.1	71.4	66.7	64.9	59.3	58.3	51.3
	T	64.7	58.8	63.3	57.9	53.1	45.9	44.0	34.7
	Q	69.8	66.2	69.4	65.9	61.4	56.4	57.1	51.0
	P	57.5	51.0	55.7	49.5	42.8	34.5	33.6	24.1
	Q,T	61.5	56.8	60.9	55.9	48.8	42.3	42.7	34.1
	Q,P	55.0	49.4	53.7	47.9	39.8	31.4	33.0	24.3
	Pex	43.3	37.3	41.7	35.7	29.9	23.0	24.4	17.1
	Q,Pex	41.5	35.6	40.2	33.9	28.5	21.6	24.4	17.3
2.00	None	83.1	77.8	82.2	77.5	80.0	75.6	75.9	70.4
	T	77.4	70.8	76.1	70.3	71.8	66.0	65.5	57.3
	Q	80.9	77.3	80.6	77.0	77.3	73.6	74.7	70.1
	P	70.9	64.0	69.6	63.3	63.0	55.6	55.5	45.9
	Q,T	74.6	69.6	74.0	69.5	68.3	62.9	64.2	57.3
	Q,P	69.0	62.9	67.9	62.5	60.5	53.1	54.4	45.5
	Pex	56.8	49.8	55.6	49.2	47.6	40.3	41.2	32.1
	Q,Pex	55.0	48.7	54.4	48.4	45.4	38.3	40.1	32.4
2.50	None	89.1	84.3	88.3	84.2	87.8	84.1	85.1	80.4
	T	84.8	78.5	83.7	78.3	82.2	77.1	77.7	70.9
	Q	87.2	84.1	87.0	84.0	85.5	82.5	84.2	80.5
	P	79.4	72.3	78.1	72.0	74.9	68.4	69.3	60.8
	Q,T	82.2	77.8	81.9	77.8	79.1	74.6	76.4	70.9
	Q,P	77.3	71.6	76.5	71.1	72.5	66.3	68.5	60.8
	Pex	65.8	58.6	65.0	57.9	59.5	52.7	53.3	45.1
	Q,Pex	64.1	57.6	63.5	57.3	57.3	50.6	52.7	44.8
3.00	None	92.7	88.7	92.1	88.7	92.0	89.2	90.3	86.7
	T	89.5	83.7	88.6	83.6	88.1	83.8	85.1	79.3
	Q	91.1	88.5	91.0	88.5	90.4	88.0	89.3	86.8
	P	84.8	78.2	83.9	78.0	82.2	76.6	78.2	70.8
	Q,T	87.2	83.4	87.0	83.3	85.6	81.9	83.8	79.3
	Q,P	83.0	77.8	82.4	77.4	79.8	74.9	77.2	70.9
	Pex	72.3	65.0	71.6	64.5	67.8	61.4	62.8	54.9
	Q,Pex	70.6	64.1	70.2	64.0	65.8	59.8	62.0	54.9

Similar Transmission within Children, Teenagers and Adults

In [13] the removal of enhanced transmission for the young was found to reduce the efficacy of targeted social distancing of children and teenagers and, in order to contain infection, required the implementation of social distancing within adult groups as well. This earlier analysis evaluated containment strategy robustness in light of the removal of enhanced relative infectivity and susceptibility (both set to 1.0), or the increase of contacts within the work environment (by a factor of 4.0) to put adults on par with children and teens at school, and the combination of both. Only the combination, given by applying both removal of enhanced infectivity and connectivity of children and teenagers and the increase of contacts in the adult work environment, is analyzed here. While we believe these 2 conditions are unlikely to occur in combination, their combination forms a bounding scenario.

Tables 28 through 31 show the infection rates with less than 10-percent and 10-percent to 25-percent zones colored green and pink, respectively. Those strategies that rely on the social distancing of children and teenagers decrease in efficacy while those that rely on social distancing of adults increase in efficacy. While the model showed roughly the same number of strategies in the pink and green zones as the core analysis in each I_F region at the 90- and 60-percent compliance levels when applied regionally (**Tables 28 and 29**), the degradation of efficacy is significant when mitigation is local-only (**Tables 30 and 31**). This degradation results from the increase (by the factor of 4) in the number of contacts within the work environment, all of which were assumed to take place with adults from surrounding communities where the epidemic remains unchecked.

The increased sensitivity of the unmitigated epidemic's infection rate to I_F is also of interest. At I_F 0.75, the unmitigated epidemic only infects 7 percent of the population (rather than 28 percent as seen for the transmission network where children and teenagers are more infective and susceptible, **Tables 7 and 9**), and at I_F 1.5, 78 percent are infected (rather than 71 percent as seen for the transmission network emphasizing children and teenagers, **Tables 7 and 9**).

Table 28: Similar transmission within children, teenagers and adults for infection rates, regionally mitigated, 90 percent compliance

For Ferguson-like disease manifestation and implementation threshold when 10 cases are diagnosed. Case-based strategy combinations vertical, network-focused strategy combinations horizontal. Green shading, infection rates 10 percent or less; pink shading, infection rates between 10 and 25 percent.

S - Schools closed	CTsd - Child/Teen social distancing	ASsd - Adult/Senior social distancing	Q - Household Quantine	T - Antiviral Treatment	P - Antiviral Prophylaxis	PEx - Extended Antiviral Prophylaxis

ID Factor		None	ASsd	CTsd	CTsd,ASsd	S	S, ASsd	S,CTsd	,CTsd,ASs
0.75	None	7.6	2.3	4.2	1.5	2.0	1.4	1.3	1.2
	T	2.3	1.4	1.7	1.2	1.5	1.2	1.2	1.2
	Q	2.2	1.4	1.6	1.3	1.5	1.2	1.4	1.2
	P	1.8	1.4	1.4	1.5	1.3	1.1	1.2	1.1
	Q,T	1.5	1.3	1.5	1.2	1.3	1.4	1.2	1.0
	Q,P	1.6	1.2	1.3	1.2	1.3	1.1	1.2	1.2
	Pex	1.3	1.3	1.4	1.3	1.3	1.2	1.0	0.0
	Q,Pex	1.4	1.2	1.4	1.2	1.2	1.1	1.0	1.1
1.00	None	48.8	17.1	41.8	9.1	21.9	3.2	3.5	1.4
	T	34.0	4.8	24.3	2.9	5.3	1.9	2.0	1.3
	Q	25.5	3.6	13.3	2.8	4.5	2.1	2.3	1.4
	P	19.7	2.6	8.7	1.9	2.9	1.5	1.6	1.3
	Q,T	14.5	2.4	5.6	1.8	2.5	1.6	1.7	1.3
	Q,P	10.1	2.0	4.4	1.6	2.3	1.5	1.6	1.2
	Pex	6.8	1.9	3.3	1.5	1.8	1.4	1.3	1.2
	Q,Pex	3.8	1.6	2.4	1.4	1.9	1.4	1.4	1.2
1.25	None	67.9	41.6	63.2	34.1	51.2	21.5	25.6	2.7
	T	58.8	28.3	52.3	17.7	36.5	5.5	7.8	1.8
	Q	49.4	21.1	44.0	12.9	29.7	6.1	9.2	2.5
	P	46.3	12.1	38.5	6.5	18.1	2.7	3.7	1.4
	Q,T	41.4	9.3	32.5	4.6	14.3	2.9	3.5	1.6
	Q,P	38.1	6.8	26.6	3.5	9.2	2.5	2.9	1.5
	Pex	28.7	5.2	20.7	3.3	6.1	2.1	2.5	1.4
	Q,Pex	20.9	3.5	11.9	2.4	4.1	1.9	2.2	1.5
1.50	None	78.1	55.9	74.6	51.7	66.8	44.9	48.6	6.8
	T	71.9	44.8	67.6	38.4	56.6	25.6	31.4	2.7
	Q	63.2	40.9	59.3	34.2	50.0	24.9	31.4	4.8
	P	60.8	30.5	55.2	21.1	42.3	8.4	16.3	2.1
	Q,T	55.8	28.3	51.0	17.3	38.4	8.5	15.1	2.4
	Q,P	52.2	21.9	46.3	11.3	31.9	5.5	9.5	1.9
	Pex	42.4	16.4	37.1	9.8	23.3	4.7	6.4	1.9
	Q,Pex	36.5	9.6	28.8	5.1	14.9	3.4	4.9	1.9
2.00	None	88.6	71.5	86.2	69.7	82.5	68.3	71.0	38.2
	T	85.1	63.7	82.1	61.0	76.4	57.5	60.2	11.8
	Q	77.9	61.1	75.9	57.9	71.5	55.2	60.2	28.2
	P	76.1	52.6	72.9	47.2	65.2	40.4	48.0	5.3
	Q,T	71.9	51.5	69.3	46.4	62.9	40.8	47.7	8.9
	Q,P	67.2	44.6	64.3	37.8	57.5	30.5	40.8	4.4
	Pex	58.8	36.2	54.9	0.0	46.2	23.9	29.3	4.0
	Q,Pex	52.9	29.8	48.6	22.9	39.8	15.1	24.2	3.7
2.50	None	93.3	79.3	91.6	78.4	89.7	79.3	81.9	59.6
	T	91.2	73.8	89.0	72.4	85.9	72.0	74.4	40.0
	Q	85.8	72.4	84.4	70.5	81.6	70.1	74.5	52.2
	P	84.4	64.5	82.0	61.5	77.4	59.5	63.9	19.3
	Q,T	80.8	64.2	79.0	61.1	75.2	59.5	65.2	29.1
	Q,P	76.0	57.3	74.2	54.0	69.8	51.3	57.8	14.6
	Pex	68.9	47.9	66.0	0.0	59.2	41.0	45.4	9.5
	Q,Pex	62.9	41.7	60.0	37.2	53.9	33.4	40.2	7.7
3.00	None	95.8	83.8	94.5	83.3	93.4	85.4	87.7	71.5
	T	94.5	79.6	92.7	78.7	90.8	80.1	82.4	57.2
	Q	90.4	78.8	89.3	77.9	87.7	78.6	82.5	66.1
	P	89.5	71.8	87.6	70.3	84.3	70.2	73.7	39.1
	Q,T	86.4	72.1	85.0	70.3	82.6	70.5	75.0	48.4
	Q,P	81.8	65.5	80.5	63.4	77.5	63.1	68.4	32.5
	Pex	75.8	55.9	73.3	0.0	68.0	51.9	55.1	22.0
	Q,Pex	69.9	50.5	67.4	47.3	62.4	45.8	50.9	16.9

Table 29: Similar transmission within children, teenagers and adults for infection rates, regionally mitigated, 60 percent compliance

For Ferguson-like disease manifestation and implementation threshold when 10 cases are diagnosed. Case-based strategy combinations vertical, network-focused strategy combinations horizontal. Green shading, infection rates 10 percent or less; pink shading, infection rates between 10 and 25 percent.

**S** - Schools closed	**CTsd** - Child/Teen social distancing	**ASsd** - Adult/Senior social distancing	**Q** - Household Quantine	**T** - Antiviral Treatment	**P** - Antiviral Prophylaxis	**PEx** - Extended Antiviral Prophylaxis

ID Factor		None	ASsd	CTsd	CTsd,ASsd	S	S, ASsd	S,CTsd	,CTsd,ASs
0.75	None	10.1	3.4	10.2	3.5	2.2	1.7	1.7	1.3
	T	2.9	1.8	2.8	1.8	1.5	1.5	1.2	1.1
	Q	4.1	2.6	5.4	2.3	1.9	1.6	1.7	1.4
	P	1.7	1.4	1.8	1.5	1.3	1.3	1.4	1.3
	Q,T	2.2	1.3	2.4	1.5	1.3	1.3	1.3	1.3
	Q,P	1.7	1.3	2.1	1.5	1.3	1.1	1.2	1.2
	Pex	1.6	1.3	1.5	1.3	1.3	1.2	1.1	1.1
	Q,Pex	1.4	1.2	1.4	1.3	1.3	1.2	1.1	1.0
1.00	None	49.6	36.2	50.1	34.2	34.0	14.4	20.5	5.0
	T	34.6	15.9	35.1	13.5	12.2	3.2	4.0	1.9
	Q	40.9	30.7	42.0	29.0	24.8	10.1	12.5	4.9
	P	23.4	7.7	21.7	4.7	4.9	2.0	2.6	1.6
	Q,T	24.6	9.6	25.9	8.3	6.2	2.7	3.2	1.9
	Q,P	15.9	4.7	13.6	4.6	3.3	2.0	2.6	1.6
	Pex	7.1	2.9	6.7	2.5	2.2	1.8	1.8	1.5
	Q,Pex	3.9	2.7	4.3	2.1	2.1	1.6	1.6	1.4
1.25	None	68.6	57.8	68.8	56.7	58.9	47.1	50.4	34.4
	T	58.7	44.5	58.7	43.0	45.3	28.0	33.2	9.5
	Q	61.1	53.9	62.0	52.9	51.9	41.9	45.4	33.2
	P	49.3	33.5	48.8	30.3	33.0	11.3	18.1	4.3
	Q,T	50.4	39.6	51.0	38.4	36.4	21.0	25.9	8.8
	Q,P	43.1	29.6	42.7	25.7	27.0	8.2	14.4	3.7
	Pex	30.3	15.1	29.9	12.8	14.0	4.6	5.3	2.2
	Q,Pex	25.3	10.8	24.7	9.4	8.8	3.7	4.9	2.4
1.50	None	78.9	70.2	78.8	69.6	72.7	64.0	66.6	54.9
	T	72.0	60.3	72.0	59.1	63.2	50.8	54.1	35.9
	Q	72.8	67.3	73.2	67.1	66.6	60.1	62.5	53.9
	P	63.2	50.1	63.1	49.1	52.7	37.7	42.6	19.3
	Q,T	64.0	56.1	64.9	56.0	55.9	45.1	49.5	34.5
	Q,P	57.8	46.8	57.9	45.8	48.2	33.4	39.4	19.4
	Pex	44.0	31.7	44.0	29.8	33.6	17.6	24.4	7.2
	Q,Pex	39.6	28.7	40.2	26.8	28.8	14.4	19.0	6.1
2.00	None	89.4	82.9	88.8	82.7	86.0	80.8	82.4	75.7
	T	85.5	76.9	84.9	76.2	80.8	73.3	75.3	64.7
	Q	85.0	81.4	85.3	81.3	82.0	78.4	79.8	75.4
	P	78.7	68.6	78.4	68.4	73.2	63.7	67.0	53.6
	Q,T	79.5	74.3	80.0	74.0	75.2	69.7	71.7	64.4
	Q,P	73.5	66.4	73.8	65.9	68.4	61.0	64.0	53.1
	Pex	60.8	49.9	60.7	49.1	54.0	43.9	47.1	32.9
	Q,Pex	57.0	47.9	57.0	47.1	50.3	41.1	44.7	32.4
2.50	None	93.9	89.0	93.4	89.0	92.0	88.5	89.7	85.1
	T	91.6	84.8	91.0	84.5	88.8	83.6	85.3	78.2
	Q	91.1	88.2	91.1	88.2	89.3	86.8	87.9	85.0
	P	86.7	78.7	86.3	78.4	83.1	76.5	78.9	69.6
	Q,T	87.5	83.3	87.4	83.2	84.8	81.1	82.6	77.9
	Q,P	82.5	77.1	82.7	76.8	79.3	74.3	76.2	69.5
	Pex	70.9	61.0	70.6	60.5	65.6	57.3	59.8	49.1
	Q,Pex	67.0	59.3	67.5	59.0	62.3	55.3	58.0	49.0
3.00	None	96.3	92.5	95.9	92.5	95.1	92.6	93.6	90.2
	T	94.7	89.4	94.2	89.3	93.0	89.2	90.5	85.5
	Q	94.4	92.0	94.3	92.0	93.3	91.3	92.2	90.0
	P	91.3	84.7	90.9	84.5	88.7	83.8	85.7	78.9
	Q,T	91.9	88.5	91.7	88.5	90.1	87.3	88.5	85.1
	Q,P	88.0	83.6	87.9	83.5	85.8	81.9	83.5	78.7
	Pex	77.4	68.5	77.3	68.4	73.4	66.3	68.5	59.6
	Q,Pex	73.9	67.4	74.2	67.2	70.5	64.4	66.6	59.1

Table 30: Similar transmission within children, teenagers and adults for infection rates, local-only mitigation, 90-percent compliance

For Ferguson-like disease manifestation and implementation threshold when 10 cases are diagnosed. Case-based strategy combinations vertical, network-focused strategy combinations horizontal. Green shading, infection rates 10 percent or less; pink shading, infection rates between 10 and 25 percent.

S - Schools closed	CTsd - Child/Teen social distancing	ASsd - Adult/Senior social distancing	Q - Household Quantine	T - Antiviral Treatment	P - Antiviral Prophylaxis	PEx - Extended Antiviral Prophylaxis

ID Factor		None	ASsd	CTsd	CTsd,ASsd	S	S, ASsd	S,CTsd	,CTsd,ASs
0.75	None	17.9	8.5	15.4	7.5	11.5	5.9	9.1	4.7
	T	13.8	6.6	12.6	6.0	9.8	4.9	7.8	4.1
	Q	14.1	6.8	13.0	6.5	10.5	5.6	8.8	4.7
	P	11.9	5.7	11.0	5.4	8.7	4.6	7.3	3.9
	Q,T	11.9	5.8	11.1	5.6	8.9	4.7	7.7	4.1
	Q,P	11.2	5.4	10.5	5.2	8.4	4.5	7.3	3.9
	Pex	10.5	5.3	10.0	5.2	8.1	4.4	7.0	3.9
	Q,Pex	10.0	5.1	9.7	4.9	7.9	4.3	6.9	3.9
1.00	None	46.2	27.5	41.4	24.0	33.6	18.8	24.8	12.8
	T	39.6	21.6	35.4	19.0	28.2	15.2	21.1	10.8
	Q	37.2	21.1	34.5	19.3	28.7	16.1	23.6	12.7
	P	33.1	17.2	30.0	15.9	24.4	12.9	19.5	10.0
	Q,T	32.1	17.3	30.0	15.9	24.7	13.3	20.5	10.7
	Q,P	30.2	15.8	27.7	14.7	23.0	12.4	19.0	10.1
	Pex	25.5	14.0	24.2	13.2	20.1	11.4	17.0	9.3
	Q,Pex	24.0	13.3	22.8	12.7	19.2	10.9	16.7	9.3
1.25	None	64.7	44.6	60.5	40.9	53.1	35.0	40.8	22.4
	T	58.6	38.0	54.4	34.5	46.2	28.3	35.3	18.8
	Q	54.5	36.5	51.7	33.5	45.5	29.3	38.4	22.2
	P	50.4	31.2	46.8	28.1	39.6	23.4	31.6	17.1
	Q,T	48.6	30.8	46.2	28.2	39.9	24.4	33.1	18.7
	Q,P	45.2	27.8	42.5	25.4	36.6	21.9	30.5	17.0
	Pex	37.7	23.0	35.8	21.6	30.2	18.7	25.9	14.8
	Q,Pex	34.8	21.3	33.3	20.1	28.7	17.9	25.2	14.8
1.50	None	76.2	57.2	72.9	53.9	67.3	49.7	54.7	33.2
	T	71.5	51.1	67.7	47.9	60.9	42.3	48.4	27.7
	Q	66.7	49.4	64.6	46.9	59.5	42.8	51.6	32.7
	P	63.0	43.8	59.7	40.3	53.1	35.1	43.3	25.0
	Q,T	60.6	43.2	58.6	40.4	53.1	36.2	45.2	27.7
	Q,P	56.7	39.2	54.6	36.6	49.2	32.4	41.5	24.7
	Pex	48.6	32.4	46.3	30.3	40.1	26.6	34.2	20.9
	Q,Pex	44.6	29.6	43.1	28.2	37.8	25.1	33.2	20.6
2.00	None	87.7	71.3	85.3	69.7	82.5	69.0	72.7	51.4
	T	84.9	66.7	82.2	64.7	78.0	62.5	66.4	44.3
	Q	80.2	65.9	79.0	64.3	75.6	62.1	69.0	51.0
	P	77.9	59.6	75.3	57.4	70.1	53.8	59.8	39.0
	Q,T	75.1	60.0	73.7	57.7	69.9	54.9	62.3	43.5
	Q,P	70.5	55.0	69.2	52.6	64.9	49.8	57.0	38.7
	Pex	63.7	46.4	61.3	44.2	54.9	40.0	46.8	31.1
	Q,Pex	58.2	42.8	56.7	40.9	51.2	37.5	45.3	30.7
2.50	None	93.0	78.8	91.3	77.9	89.7	79.2	82.4	64.3
	T	91.2	75.3	89.3	74.2	86.7	74.6	77.4	57.1
	Q	87.3	75.3	86.4	74.3	84.4	73.7	79.2	64.0
	P	86.0	69.6	83.9	68.0	80.3	66.5	70.8	51.0
	Q,T	83.1	70.2	82.1	68.6	79.6	67.6	73.4	56.3
	Q,P	78.7	65.1	77.7	63.7	74.6	61.8	67.6	50.2
	Pex	73.5	56.8	71.3	54.9	65.4	51.0	56.7	40.0
	Q,Pex	67.9	52.9	66.1	51.0	61.2	47.8	54.6	39.7
3.00	None	95.7	83.3	94.5	82.7	93.4	85.1	88.0	72.9
	T	94.5	80.3	93.0	79.6	91.4	81.4	84.0	66.3
	Q	91.4	80.8	90.6	80.2	89.2	80.6	85.3	72.3
	P	90.6	75.7	89.0	74.6	86.2	74.6	77.9	59.9
	Q,T	88.1	76.5	87.2	75.5	85.4	75.4	80.2	65.5
	Q,P	84.0	71.7	83.2	70.6	80.9	70.2	74.8	58.8
	Pex	80.1	64.1	78.0	62.4	72.8	59.6	64.0	47.3
	Q,Pex	74.2	60.4	73.0	58.7	68.6	56.0	61.6	46.8

Table 31: Similar transmission within children, teenagers and adults for infection rates, local-only mitigation, 60 percent compliance

For Ferguson-like disease manifestation and implementation threshold when 10 cases are diagnosed. Case-based strategy combinations vertical, network-focused strategy combinations horizontal. Green shading, infection rates 10 percent or less; pink shading, infection rates between 10 and 25 percent.

S - Schools closed	CTsd - Child/Teen social distancing	ASsd - Adult/Senior social distancing	Q - Household Quantine	T - Antiviral Treatment	P - Antiviral Prophylaxis	PEx - Extended Antiviral Prophylaxis

ID Factor		None	ASsd	CTsd	CTsd,ASsd	S	S, ASsd	S,CTsd	,CTsd,ASs
0.75	None	17.4	10.4	17.6	10.1	12.3	7.3	10.7	6.3
	T	13.5	7.6	13.9	7.6	10.4	6.1	9.3	5.4
	Q	15.5	9.2	16.1	9.2	11.7	6.9	10.7	6.3
	P	11.9	6.8	12.3	6.8	9.7	5.7	8.7	5.1
	Q,T	12.4	7.3	13.2	7.0	10.0	6.0	9.1	5.6
	Q,P	11.5	6.6	11.8	6.4	9.4	5.7	8.6	5.2
	Pex	10.6	6.1	10.7	6.0	8.9	5.4	8.1	4.9
	Q,Pex	10.1	5.9	10.5	5.9	8.8	5.4	8.2	5.0
1.00	None	45.7	34.4	45.7	33.3	35.5	24.1	30.6	19.3
	T	38.8	26.7	39.0	25.8	30.2	19.7	26.0	16.0
	Q	41.3	31.4	42.3	30.6	32.9	22.2	29.8	19.3
	P	34.2	22.4	34.3	21.5	27.1	17.0	23.7	14.6
	Q,T	34.9	24.4	35.9	23.5	28.1	18.1	25.4	16.1
	Q,P	31.6	21.0	32.3	20.3	25.7	16.5	23.5	14.6
	Pex	26.5	17.3	27.0	16.7	22.6	14.4	20.6	13.0
	Q,Pex	25.4	16.6	25.9	16.2	21.8	14.1	20.4	13.0
1.25	None	64.1	53.1	64.3	52.1	55.4	43.0	49.3	35.5
	T	58.0	45.7	58.0	44.5	49.1	36.0	43.1	29.4
	Q	59.2	49.8	60.0	49.2	51.3	40.1	47.8	35.4
	P	51.9	39.0	51.9	37.7	43.7	30.9	38.8	25.9
	Q,T	52.8	42.0	53.8	41.2	45.5	33.3	41.6	29.2
	Q,P	48.2	36.8	48.9	35.9	41.6	29.4	38.1	25.9
	Pex	39.1	28.5	39.8	27.9	34.2	24.0	31.1	21.2
	Q,Pex	37.1	26.9	37.9	26.5	32.8	23.3	30.5	21.2
1.50	None	76.0	65.7	75.7	65.1	69.5	58.8	64.2	51.0
	T	71.2	59.3	71.1	58.4	64.2	51.6	57.9	43.5
	Q	71.4	62.9	72.1	62.8	65.6	55.6	62.3	50.7
	P	64.8	52.7	64.9	51.8	57.8	45.1	52.6	38.2
	Q,T	65.8	55.8	66.4	55.5	59.5	48.3	55.8	43.3
	Q,P	60.6	50.2	61.6	49.4	54.8	42.9	50.9	38.0
	Pex	50.5	38.8	50.8	38.2	44.8	33.9	41.1	29.9
	Q,Pex	47.8	37.2	48.6	36.7	43.0	32.7	40.2	29.6
2.00	None	87.9	79.6	87.3	79.4	84.6	76.9	80.8	70.9
	T	85.0	75.2	84.3	74.7	80.6	71.4	75.8	64.1
	Q	84.4	78.3	84.6	78.1	81.1	74.5	78.8	70.9
	P	79.7	69.4	79.5	68.7	75.0	64.6	70.3	57.8
	Q,T	80.4	73.0	80.7	72.8	76.5	68.2	73.7	64.1
	Q,P	75.6	67.2	76.2	67.0	71.5	62.5	68.4	57.6
	Pex	65.6	54.0	65.7	53.5	59.9	49.0	55.6	43.8
	Q,Pex	62.3	52.2	62.9	51.9	57.5	47.3	54.2	43.4
2.50	None	93.2	86.9	92.7	86.7	91.3	86.1	88.8	82.2
	T	91.4	83.6	90.8	83.4	88.7	82.3	85.4	76.8
	Q	90.7	86.2	90.8	86.1	88.8	84.3	87.4	82.0
	P	87.6	79.1	87.2	78.7	84.3	76.7	80.5	70.8
	Q,T	88.0	82.3	88.1	82.1	85.5	79.7	83.5	76.6
	Q,P	84.0	77.4	84.3	77.4	81.2	74.4	78.8	70.5
	Pex	75.1	64.5	75.0	64.0	70.1	60.4	65.9	54.9
	Q,Pex	71.7	62.9	72.2	62.6	67.5	58.7	64.6	54.7
3.00	None	95.9	91.1	95.5	91.0	94.7	91.0	93.0	88.1
	T	94.6	88.4	94.1	88.3	93.0	88.1	90.5	84.1
	Q	94.2	90.6	94.0	90.6	93.0	89.7	91.9	88.0
	P	92.0	84.8	91.5	84.6	89.6	83.8	86.7	79.1
	Q,T	92.2	87.6	92.1	87.5	90.5	86.2	89.0	83.9
	Q,P	89.0	83.7	89.1	83.5	87.0	81.8	85.2	78.9
	Pex	81.4	71.9	81.2	71.4	77.1	68.7	73.2	63.4
	Q,Pex	78.3	70.2	78.5	70.0	74.6	66.9	71.9	63.2

Augmented Contact Network

Breaking pre-schoolers out into their own groups, adding additional contact groups to all age classes within the community (following the findings of a survey by Glass and Glass [29]) and removing enhanced relative infectivity and susceptibility of children and teenagers (to keep the age class specific attack rates similar) did not yield large differences in the overall results. **Tables 32 through 35** show infection rates for the augmented social networks with less than 10-percent and 10-percent to 25-percent zones colored green and pink, respectively. The percentage of the population infected as a function of I_F for the unmitigated epidemic is the same as in the base contact network. However, the number of intervention combinations that are within the green zone decrease for all I_F from the base to the augmented contact network. The largest differences between augmented and base contact networks occur for strategies that were green for the base and are now pink for the augmented. Far within the green zone for the augmented contact network, where strategies are most effective (to the right and downward), differences are small.

At an I_F of 1.5, when the best strategy is implemented (P+S+CTsd+ASsd), the epidemic remains well controlled with the number of infected, deaths and antiviral courses the same as for the base contact network (within the *SD* of the measure). Adult days out of work are also similar for augmented network and core (7 vs. 6 days). When compliance is reduced to 60%, required adults days out triple for both networks, and double when mitigation is local-only. There are no differences in number of cycles required at any I_F between the 2 networks and time to peak symptomatic cases varies little between the two networks with best strategies applied (13 days core vs 15 days augmented), but the difference is extended with reduced compliance (33 days core vs 43 days augmented).

Table 32: Augmented Contact Network, for infection rates, regional mitigation, 90-percent compliance

For Ferguson-like disease manifestation and implementation threshold when 10 cases are diagnosed. Case-based strategy combinations vertical, network-focused strategy combinations horizontal. Green shading, infection rates 10 percent or less; pink shading, infection rates between 10 and 25 percent.

**S** - Schools closed	**CTsd** - Child/Teen social distancing	**ASsd** - Adult/Senior social distancing	**Q** - Household Quantine	**T** - Antiviral Treatment	**P** - Antiviral Prophylaxis	**PEx** - Extended Antiviral Prophylaxis

ID Factor		None	ASsd	CTsd	CTsd, ASsd	S	S, ASsd	S, CTsd	S,CTsd, ASsd
0.75	None	31.3	28.1	24.8	12.5	6.8	5.3	1.5	1.4
	T	23.3	21.0	11.5	5.5	3.6	2.5	1.3	1.1
	Q	19.6	20.3	8.1	5.1	4.0	3.1	1.5	1.2
	P	17.3	16.8	4.6	3.0	2.4	2.3	1.4	1.2
	Q,T	15.5	15.5	3.9	3.0	2.3	2.4	1.3	1.2
	Q,P	15.0	13.3	3.4	2.5	2.3	1.9	1.2	1.1
	Pex	7.8	7.8	3.5	2.5	1.9	1.8	1.3	1.4
	Q,Pex	5.9	6.3	2.5	1.8	1.8	1.4	1.5	1.1
1.00	None	50.8	42.8	49.4	35.0	30.4	20.0	4.2	1.8
	T	43.1	36.6	40.5	26.6	20.3	12.2	2.3	1.4
	Q	38.2	35.0	35.2	25.5	18.9	13.4	2.4	1.8
	P	34.6	30.1	30.4	17.9	11.6	7.9	1.8	1.4
	Q,T	32.2	29.4	26.2	15.6	11.4	8.1	1.8	1.5
	Q,P	29.7	27.3	22.5	11.6	9.7	7.3	1.5	1.3
	Pex	21.7	19.2	17.3	9.0	6.1	4.2	1.8	1.5
	Q,Pex	18.8	17.3	10.9	5.2	4.4	3.5	1.5	1.5
1.25	None	62.9	52.1	62.9	47.5	48.5	33.6	22.7	3.4
	T	56.4	46.2	56.1	39.8	38.5	24.8	7.6	2.2
	Q	50.9	45.2	50.0	38.6	36.3	27.5	10.5	3.1
	P	47.1	39.4	46.2	31.7	27.4	17.4	3.5	1.8
	Q,T	44.5	39.3	43.1	31.3	26.4	18.4	4.2	1.9
	Q,P	41.2	35.6	39.5	26.4	22.8	15.5	2.7	1.6
	Pex	32.5	26.9	31.7	19.1	17.2	9.8	3.0	1.6
	Q,Pex	29.0	24.4	26.7	14.8	12.6	7.8	2.6	1.7
1.50	None	71.5	58.7	71.5	55.6	60.9	44.2	43.1	8.2
	T	65.9	53.3	66.2	48.8	51.8	34.9	26.7	3.3
	Q	59.9	52.7	60.4	48.1	49.3	37.8	29.9	7.1
	P	56.3	45.9	56.5	40.5	40.8	26.7	12.1	2.3
	Q,T	53.6	46.4	53.8	40.5	40.0	28.7	15.0	2.8
	Q,P	49.5	41.7	49.6	35.8	35.3	23.7	8.8	2.3
	Pex	41.3	32.8	41.2	26.8	27.3	16.6	8.3	2.1
	Q,Pex	37.2	30.3	36.7	23.6	22.5	14.3	6.3	2.0
2.00	None	81.7	67.7	81.6	66.2	75.7	59.5	66.0	34.9
	T	77.9	62.5	77.8	60.5	69.5	51.0	54.3	11.8
	Q	72.5	63.0	72.9	60.8	66.3	53.6	56.7	31.0
	P	69.3	55.3	69.9	52.4	59.0	40.8	41.4	5.8
	Q,T	66.6	56.7	67.3	53.5	58.5	43.7	44.6	9.8
	Q,P	62.0	51.3	63.0	48.0	52.6	36.9	36.5	5.6
	Pex	54.1	42.3	54.4	38.5	41.9	27.4	28.2	4.3
	Q,Pex	49.4	39.1	49.3	35.0	37.9	24.8	24.4	4.6
2.50	None	87.8	73.5	87.5	72.9	84.0	69.1	77.8	53.4
	T	84.7	68.8	84.8	67.8	79.3	61.7	69.4	33.9
	Q	80.4	69.7	80.9	68.3	76.7	64.1	71.1	49.4
	P	77.7	61.9	78.1	60.2	70.7	51.9	58.3	15.5
	Q,T	75.4	63.8	75.9	62.0	70.1	55.1	61.1	29.0
	Q,P	70.6	58.2	71.5	56.0	64.6	48.0	53.6	14.1
	Pex	62.7	48.7	63.1	46.0	53.2	36.2	41.2	8.8
	Q,Pex	58.4	45.9	58.5	42.6	49.3	33.3	38.2	8.6
3.00	None	91.4	77.4	91.2	77.1	89.0	75.4	84.8	64.8
	T	89.2	73.2	88.9	72.6	85.6	69.1	78.7	49.4
	Q	85.9	74.3	86.0	73.6	83.4	70.9	79.7	61.1
	P	83.5	66.7	83.8	65.9	78.5	60.1	68.9	32.4
	Q,T	81.5	69.0	82.0	67.8	77.8	63.3	71.9	45.2
	Q,P	76.9	63.2	77.8	61.9	72.8	56.3	64.7	30.0
	Pex	69.3	54.1	69.9	52.2	61.2	43.4	50.9	19.3
	Q,Pex	64.4	51.2	65.1	48.9	57.3	40.5	47.4	17.4

Table 33: Augmented Contact Network, for infection rates, regional mitigation, 60 percent compliance

For Ferguson-like disease manifestation and implementation threshold when 10 cases are diagnosed. Case-based strategy combinations vertical, network-focused strategy combinations horizontal. Green shading, infection rates 10

S - Schools closed	CTsd - Child/Teen social distancing	ASsd - Adult/Senior social distancing	Q - Household Quantine	T - Antiviral Treatment	P - Antiviral Prophylaxis	PEx - Extended Antiviral Prophylaxis

ID Factor		None	ASsd	CTsd	CTsd,ASsd	S	S, ASsd	S,CTsd	,CTsd,ASs
0.75	None	31.7	31.7	33.3	27.6	8.4	6.0	2.1	1.9
	T	22.9	23.9	21.3	16.9	3.6	2.7	1.6	1.3
	Q	27.6	28.5	27.8	24.1	5.3	4.4	1.8	1.8
	P	18.9	19.3	14.9	11.6	2.5	2.5	1.5	1.4
	Q,T	18.9	20.6	15.9	14.2	2.9	2.1	1.4	1.3
	Q,P	15.8	16.3	12.2	8.8	2.5	2.5	1.4	1.3
	Pex	9.2	9.5	6.1	4.9	1.9	2.0	1.5	1.2
	Q,Pex	7.7	8.8	4.7	3.7	1.8	1.6	1.4	1.2
1.00	None	51.1	48.2	53.4	46.1	33.5	25.8	18.8	8.8
	T	42.8	41.0	45.1	37.9	22.2	15.6	5.4	2.6
	Q	45.4	45.3	47.7	43.6	28.8	23.8	15.6	8.8
	P	36.1	35.1	37.8	31.0	14.9	11.3	3.2	2.1
	Q,T	36.9	36.9	39.3	34.6	15.9	13.7	4.2	2.6
	Q,P	32.8	32.6	33.8	29.3	12.2	8.5	2.7	2.1
	Pex	23.6	22.9	24.8	19.6	8.0	6.1	2.5	2.0
	Q,Pex	21.9	21.3	22.1	17.4	6.8	5.2	2.4	1.9
1.25	None	63.4	59.2	65.4	58.0	52.0	43.7	43.8	32.1
	T	56.4	52.2	58.8	50.5	40.9	32.4	29.2	13.9
	Q	58.0	56.8	60.4	56.0	46.5	41.3	39.9	31.5
	P	49.0	45.8	51.5	43.5	33.4	24.0	17.4	6.3
	Q,T	50.6	49.5	53.2	47.5	35.8	29.6	24.6	13.4
	Q,P	45.2	43.5	47.5	41.3	29.4	22.8	15.0	5.9
	Pex	34.4	31.9	36.6	29.1	22.2	15.5	10.7	4.0
	Q,Pex	32.2	29.9	34.1	28.0	19.3	13.6	9.2	4.3
1.50	None	71.7	66.8	73.5	66.1	64.0	56.2	58.6	48.4
	T	66.1	60.6	68.1	59.5	55.8	46.0	47.5	33.3
	Q	67.0	65.3	69.0	64.6	59.3	53.9	54.8	47.8
	P	58.8	54.1	61.2	52.4	47.1	37.4	37.0	21.9
	Q,T	60.2	58.5	62.6	57.1	49.9	43.1	42.8	32.8
	Q,P	54.3	51.9	56.9	50.7	42.8	35.2	34.2	20.9
	Pex	43.1	39.0	45.4	37.7	32.8	24.8	25.1	12.6
	Q,Pex	40.8	37.4	42.6	36.1	30.1	22.7	23.3	12.2
2.00	None	82.2	76.6	83.1	76.5	78.5	71.7	75.3	67.7
	T	78.0	71.6	79.3	71.1	72.5	64.2	67.9	57.0
	Q	78.4	75.9	79.5	75.8	74.6	70.3	72.6	67.2
	P	71.7	65.2	73.3	64.6	64.9	55.6	59.2	46.9
	Q,T	73.3	70.2	74.7	69.8	67.7	62.0	64.2	56.6
	Q,P	67.5	63.8	69.5	63.3	61.0	53.9	56.4	46.9
	Pex	56.0	49.7	57.6	48.7	48.2	39.7	43.0	32.2
	Q,Pex	53.0	48.4	54.8	47.6	45.5	38.1	41.0	32.1
2.50	None	88.2	82.8	88.6	82.6	86.0	80.5	84.1	77.9
	T	84.9	78.4	85.6	78.2	81.7	74.7	78.9	70.2
	Q	85.2	82.2	86.0	82.1	83.2	79.5	82.0	77.7
	P	79.8	72.8	81.0	72.5	75.7	67.4	72.0	62.2
	Q,T	81.2	77.6	82.1	77.3	78.1	73.1	75.9	70.0
	Q,P	76.1	72.0	77.6	71.7	72.3	65.9	69.6	61.7
	Pex	64.4	57.7	65.9	57.1	58.4	49.8	54.5	44.8
	Q,Pex	61.5	56.5	63.2	56.0	56.0	48.8	52.4	44.6
3.00	None	91.7	86.8	92.0	86.8	90.5	85.9	89.4	84.0
	T	89.4	83.2	89.7	82.9	87.4	81.1	85.5	78.2
	Q	89.6	86.5	90.1	86.4	88.4	85.0	87.5	83.8
	P	85.3	78.3	86.0	78.1	82.5	75.1	79.9	71.6
	Q,T	86.4	82.6	87.0	82.5	84.4	80.0	83.1	78.0
	Q,P	82.0	77.6	83.2	77.5	79.8	74.1	77.8	71.1
	Pex	70.7	63.6	72.2	63.3	66.2	57.9	62.3	53.7
	Q,Pex	68.2	62.5	69.6	62.4	63.5	56.8	60.9	53.5

percent or less; pink shading, infection rates between 10 and 25 percent.

Table 34: Augmented Contact Network, for infection rates, local-only mitigation, 90 percent compliance

For Ferguson-like disease manifestation and implementation threshold when 10 cases are diagnosed. Case-based strategy combinations vertical, network-focused strategy combinations horizontal. Green shading, infection rates 10 percent or less; pink shading, infection rates between 10 and 25 percent.

S - Schools closed	CTsd - Child/Teen social distancing	ASsd - Adult/Senior social distancing	Q - Household Quantine	T - Antiviral Treatment	P - Antiviral Prophylaxis	PEx - Extended Antiviral Prophylaxis

ID Factor		None	ASsd	CTsd	CTsd, ASsd	S	S, ASsd	S, CTsd	S,CTsd, ASsd
0.75	None	30.5	28.0	24.5	15.6	12.5	7.7	5.4	3.1
	T	24.2	22.3	17.3	9.4	8.3	4.6	4.5	2.4
	Q	23.5	21.8	15.4	9.6	8.9	5.6	5.4	3.0
	P	19.9	17.7	12.1	6.2	6.4	3.8	4.2	2.4
	Q,T	18.9	16.8	11.5	5.6	6.7	3.6	4.5	2.4
	Q,P	17.7	15.7	10.3	5.1	5.9	3.4	4.2	2.4
	Pex	12.6	10.1	9.1	4.4	5.5	3.2	4.0	2.3
	Q,Pex	11.4	9.2	8.0	4.1	5.2	3.1	4.0	2.4
1.00	None	49.8	43.0	48.0	36.0	33.7	23.4	17.7	7.6
	T	43.8	37.7	41.0	29.4	26.6	17.3	13.8	6.1
	Q	41.8	37.0	38.4	28.6	27.4	19.1	16.2	7.6
	P	37.6	32.3	33.9	22.7	21.2	13.6	12.0	5.4
	Q,T	36.3	31.8	32.2	22.5	21.6	13.9	12.8	6.0
	Q,P	34.3	29.7	29.8	19.5	19.4	12.4	11.6	5.4
	Pex	26.8	22.2	23.6	14.7	15.6	9.1	10.6	5.2
	Q,Pex	24.4	20.2	20.8	12.3	14.4	8.5	10.4	5.4
1.25	None	62.1	52.4	61.5	48.0	49.6	36.6	33.0	15.0
	T	56.8	47.7	56.1	42.3	42.3	29.4	26.0	11.2
	Q	53.9	47.4	52.7	41.7	42.6	31.9	30.0	15.1
	P	49.9	41.9	48.2	35.3	35.0	24.1	21.5	9.6
	Q,T	48.3	41.8	46.7	35.2	35.5	25.0	23.7	11.2
	Q,P	45.1	38.7	43.4	31.8	31.8	22.0	20.7	9.7
	Pex	37.0	30.4	34.8	24.4	24.7	16.5	17.5	8.7
	Q,Pex	34.1	28.1	31.4	21.7	22.9	15.1	16.9	8.7
1.50	None	70.8	59.1	70.5	56.4	61.8	47.0	47.6	25.3
	T	66.3	54.8	66.2	51.1	54.8	40.0	39.0	18.5
	Q	62.9	55.2	62.8	51.1	54.3	42.5	43.9	25.0
	P	59.1	49.2	58.6	44.9	46.6	33.1	32.5	15.2
	Q,T	57.5	49.8	57.2	44.9	47.3	35.1	35.4	17.8
	Q,P	53.8	46.0	53.1	40.8	42.7	30.8	31.0	15.1
	Pex	45.6	37.3	44.4	32.2	33.7	23.3	24.5	12.9
	Q,Pex	42.2	34.9	40.4	29.4	31.1	21.5	23.7	12.6
2.00	None	81.3	68.0	81.1	66.7	76.3	61.3	67.2	44.0
	T	78.1	64.1	77.9	62.3	70.9	54.6	58.8	33.7
	Q	74.9	65.4	75.1	63.6	69.9	57.7	63.1	44.3
	P	71.6	59.2	71.6	56.7	63.0	47.5	50.4	27.3
	Q,T	69.9	60.4	70.3	57.8	63.3	50.4	54.5	33.1
	Q,P	65.4	55.9	65.8	53.1	58.4	44.7	47.8	27.2
	Pex	58.1	47.2	57.7	43.9	47.1	34.2	37.0	21.0
	Q,Pex	53.7	44.7	53.0	41.0	44.0	32.6	35.3	20.8
2.50	None	87.7	73.6	87.3	72.9	84.3	70.2	78.6	57.9
	T	85.0	70.2	84.8	69.3	80.6	64.8	71.9	47.6
	Q	82.2	71.9	82.3	70.9	79.2	67.3	74.9	57.7
	P	79.6	65.7	79.7	64.3	73.6	58.0	63.5	39.4
	Q,T	77.9	67.5	78.5	66.0	74.0	60.8	67.5	47.3
	Q,P	73.5	62.8	74.0	61.1	68.8	55.2	60.7	39.2
	Pex	66.7	54.7	66.8	52.3	57.5	43.6	47.2	29.2
	Q,Pex	62.1	51.8	61.9	49.0	53.9	41.2	45.3	28.7
3.00	None	91.3	77.4	91.1	77.0	89.3	76.0	85.2	67.0
	T	89.3	74.4	89.1	73.9	86.4	71.5	80.1	58.0
	Q	87.1	76.3	87.2	75.6	85.1	73.8	82.0	66.7
	P	84.9	70.2	85.1	69.6	80.6	65.1	72.5	49.5
	Q,T	83.4	72.3	83.8	71.5	80.7	68.2	76.1	57.5
	Q,P	79.3	67.9	79.8	66.8	76.0	62.7	69.7	49.4
	Pex	73.1	60.1	73.2	58.2	65.5	50.9	55.7	36.5
	Q,Pex	68.5	57.7	68.6	55.4	61.5	48.7	53.7	36.5

Table 35: Augmented Contact Network, for infection rates, local-only mitigation, 90 percent compliance

For Ferguson-like disease manifestation and implementation threshold when 10 cases are diagnosed. Case-based strategy combinations vertical, network-focused strategy combinations horizontal. Green shading, infection rates 10 percent or less; pink shading, infection rates between 10 and 25 percent

**S** - Schools closed	**CTsd** - Child/Teen social distancing	**ASsd** - Adult/Senior social distancing	**Q** - Household Quantine	**T** - Antiviral Treatment	**P** - Antiviral Prophylaxis	**PEx** - Extended Antiviral Prophylaxis

ID Factor		None	ASsd	CTsd	CTsd, ASsd	S	S, ASsd	S, CTsd	S,CTsd, ASsd
0.75	None	30.9	30.6	31.5	26.9	13.5	9.4	8.1	4.9
	T	24.3	24.3	23.2	18.5	9.3	6.0	6.1	3.7
	Q	28.1	27.9	27.8	24.6	11.8	8.4	7.9	4.8
	P	21.1	20.4	19.2	14.8	7.5	5.0	5.6	3.4
	Q,T	21.6	21.5	20.1	16.0	8.4	5.2	6.0	3.6
	Q,P	19.3	18.8	17.2	12.8	7.3	4.6	5.8	3.4
	Pex	14.2	12.8	12.9	8.5	6.7	4.4	5.3	3.3
	Q,Pex	13.1	11.0	11.5	7.9	6.4	4.1	5.3	3.2
1.00	None	50.4	47.0	51.8	45.0	35.3	27.5	27.1	17.5
	T	44.3	41.5	46.0	38.4	28.8	21.3	20.7	11.9
	Q	46.6	44.5	48.1	42.9	32.1	25.8	26.1	17.5
	P	39.4	36.7	40.4	33.2	24.5	17.8	17.7	10.2
	Q,T	40.7	38.1	41.8	35.7	26.0	19.1	19.6	12.2
	Q,P	37.0	34.5	38.1	31.6	23.2	16.8	17.2	10.0
	Pex	29.2	26.0	29.6	23.0	19.0	13.0	14.9	9.1
	Q,Pex	27.6	24.8	27.8	21.5	18.0	12.4	14.4	8.9
1.25	None	62.5	57.7	64.2	56.5	52.0	43.4	45.2	34.1
	T	57.5	52.6	59.4	51.0	45.3	35.7	37.1	25.3
	Q	58.9	55.7	60.6	54.7	48.3	41.1	43.5	34.1
	P	52.1	47.2	53.8	45.6	39.5	30.8	32.0	20.7
	Q,T	53.2	49.9	55.1	48.5	41.3	33.7	36.0	25.4
	Q,P	49.1	45.3	50.5	43.6	37.3	29.1	31.1	20.9
	Pex	39.5	35.1	40.9	32.9	29.2	21.9	24.1	16.3
	Q,Pex	37.7	33.8	38.5	31.7	27.9	20.6	23.9	16.1
1.50	None	71.1	65.3	72.6	64.8	64.0	55.3	59.1	48.3
	T	67.0	60.8	68.5	59.8	58.0	48.4	51.6	39.3
	Q	67.8	64.0	69.4	63.5	60.5	53.4	57.0	48.0
	P	61.7	55.7	63.2	54.6	51.9	42.1	45.3	32.7
	Q,T	62.9	58.9	64.5	57.9	54.0	45.8	49.4	39.0
	Q,P	58.5	53.9	60.1	52.8	49.0	40.6	43.7	32.8
	Pex	48.5	43.0	49.7	41.2	38.9	30.6	33.7	24.0
	Q,Pex	46.2	41.3	47.4	39.6	36.8	29.5	32.8	23.9
2.00	None	81.8	75.4	82.6	75.2	78.0	70.3	75.3	66.2
	T	78.6	71.6	79.7	71.1	73.7	64.6	69.5	58.8
	Q	79.2	74.6	79.9	74.4	75.2	68.7	73.4	66.1
	P	74.1	66.9	75.1	66.3	68.1	58.4	63.3	51.8
	Q,T	75.1	70.3	76.4	70.1	70.2	62.8	67.5	58.7
	Q,P	70.8	65.7	72.3	65.2	65.2	56.8	61.8	51.6
	Pex	60.9	53.8	62.2	52.8	52.8	44.0	48.1	37.9
	Q,Pex	58.2	52.4	59.7	51.5	50.8	42.7	46.9	37.6
2.50	None	88.1	81.6	88.4	81.5	86.1	79.2	84.2	76.5
	T	85.6	78.2	86.1	78.1	82.7	74.6	79.9	70.6
	Q	85.8	81.3	86.3	81.2	83.6	78.4	82.6	76.3
	P	81.8	74.2	82.7	73.9	77.9	69.1	74.7	64.5
	Q,T	82.6	77.5	83.4	77.4	79.6	73.4	78.1	70.7
	Q,P	78.8	73.4	79.8	73.1	75.2	68.0	73.1	64.4
	Pex	69.3	61.6	70.4	60.9	62.8	53.6	58.7	48.6
	Q,Pex	66.7	60.4	67.9	59.7	60.5	52.5	57.3	48.5
3.00	None	91.8	86.0	92.0	85.9	90.6	84.8	89.4	83.0
	T	89.9	82.7	90.1	82.7	87.9	80.9	86.3	78.2
	Q	89.9	85.7	90.3	85.7	88.8	84.1	88.0	82.8
	P	86.9	79.3	87.4	79.1	84.3	76.4	82.0	72.9
	Q,T	87.4	82.5	87.9	82.4	85.6	80.0	84.5	78.1
	Q,P	84.2	78.8	85.0	78.6	81.9	75.2	80.4	72.9
	Pex	75.6	67.4	76.4	66.9	70.1	61.2	66.6	56.8
	Q,Pex	73.0	66.4	74.1	66.0	68.0	60.2	65.2	56.9

Administering Pre-pandemic Vaccine

In [12], vaccination focused on children and teens was found to be most effective in limiting infection and illness with the least vaccine in the base contact network. Targeting these groups with the full proposed stockpile (7 percent of the population or 700 doses in our community of 10,000) of partially effective vaccine (assuming 50-percent efficacy at reducing transmission) yields much greater benefit than administering it either randomly throughout age classes or focused entirely on adults.

In **Tables 36 through 39**, the expanding zones of green and pink show that vaccination targeting children and teens enlarges the pool of effective (less than 10-percent and less than 25-percent infection attack rate) strategies available for influenza strains at each I_F. The greatest relative benefit is found in the 10- to 25-percent infection attack rate zone (pink zone) where vaccination has reduced infection rates by as much as 14 percent (compared to the core analysis with no vaccination), moving many of the combined interventions into the green zone (10-percent or less attack rate). Therefore, required antiviral courses decreased (by up to 47-percent coverage) as did days adults are at home (by up to 7 days).

In the zone where the infection rate was already 10 percent or less without vaccination (core analysis, green zone), added benefit from targeted vaccination with pre-pandemic vaccine is, in general, much less with only small reductions in needed antiviral courses given or days adults are at home. Thus, the increased benefit afforded by pre-pandemic vaccination does not influence the choice of best community mitigation strategy.

Table 36: Children and teenager targeted pre-pandemic vaccination on infection rates, regionally mitigated, 90 percent compliance

For Ferguson-like disease manifestation and implementation threshold when 10 cases are diagnosed. Case-based strategy combinations vertical, network-focused strategy combinations horizontal. Green shading, infection rates 10 percent or less; pink shading, infection rates between 10 and 25 percent.

S - Schools closed	CTsd - Child/Teen social distancing	ASsd - Adult/Senior social distancing	Q - Household Quantine	T - Antiviral Treatment	P - Antiviral Prophylaxis	PEx - Extended Antiviral Prophylaxis

ID Factor		None	ASsd	CTsd	CTsd,ASsd	S	S, ASsd	S,CTsd	,CTsd,ASs
0.75	None	13.8	6.7	3.6	2.5	1.6	1.6	1.3	1.2
	T	6.0	2.4	1.8	1.6	1.3	1.3	1.2	1.1
	Q	4.0	2.4	1.7	1.6	1.4	1.4	1.3	1.2
	P	3.0	1.9	1.5	1.4	1.3	1.3	1.0	1.2
	Q,T	2.1	1.7	1.3	1.4	1.4	1.2	1.2	0.0
	Q,P	2.4	1.4	1.6	1.4	1.2	1.2	1.4	1.2
	Pex	1.9	1.5	1.3	1.3	1.3	1.3	1.4	0.0
	Q,Pex	1.8	1.4	1.2	1.2	1.3	1.2	0.0	0.0
1.00	None	39.8	27.8	26.8	16.2	8.3	4.9	1.6	1.4
	T	31.1	18.7	13.2	6.2	3.5	2.0	1.3	1.3
	Q	24.6	14.0	7.2	4.6	3.1	2.4	1.6	1.5
	P	21.8	9.6	4.5	3.2	2.1	1.7	1.3	1.2
	Q,T	16.5	7.7	3.4	2.6	1.9	1.8	1.2	1.4
	Q,P	14.0	5.5	2.5	2.4	1.7	1.5	1.4	1.3
	Pex	9.6	4.6	2.9	2.2	1.6	1.5	1.6	1.2
	Q,Pex	5.4	3.1	2.1	1.7	1.7	1.5	1.2	1.2
1.25	None	54.7	40.0	44.8	32.7	36.3	23.0	2.9	1.9
	T	47.1	33.1	34.7	22.3	19.8	8.7	1.8	1.6
	Q	41.5	31.0	27.9	18.9	15.3	8.3	2.5	1.9
	P	37.8	25.5	21.6	11.6	7.2	3.8	1.5	1.3
	Q,T	34.3	23.2	16.9	9.3	6.1	3.5	1.6	1.4
	Q,P	31.4	19.6	11.2	6.7	4.3	2.9	1.5	1.4
	Pex	24.2	15.1	10.8	5.0	3.7	2.5	1.6	1.4
	Q,Pex	18.9	9.9	5.6	2.9	2.8	2.2	1.4	1.4
1.50	None	64.4	47.9	56.6	42.7	52.4	38.9	9.4	3.0
	T	58.1	41.6	48.3	34.4	40.0	26.3	3.3	1.9
	Q	52.3	40.6	42.2	32.6	34.8	24.7	5.2	2.8
	P	48.4	34.4	36.9	25.3	24.8	11.4	2.2	1.7
	Q,T	45.8	33.6	33.4	22.5	22.0	10.2	2.5	1.9
	Q,P	42.1	29.7	27.8	17.4	14.7	6.6	1.9	1.8
	Pex	33.6	23.1	23.8	13.7	10.7	5.4	2.0	1.4
	Q,Pex	29.2	19.0	14.2	8.3	6.5	3.8	1.8	1.6
2.00	None	76.8	58.1	71.3	55.3	70.5	57.8	43.5	12.4
	T	72.2	52.3	65.3	48.4	63.5	49.0	21.5	4.3
	Q	66.7	52.9	59.8	48.3	58.0	47.6	28.1	10.8
	P	63.3	45.5	55.2	40.3	51.4	36.8	7.3	2.8
	Q,T	60.7	46.2	52.4	39.5	49.0	36.4	9.3	3.8
	Q,P	56.3	41.7	47.2	34.2	43.3	29.2	5.5	2.9
	Pex	46.8	33.4	39.7	27.8	34.4	22.4	4.8	2.5
	Q,Pex	42.2	29.9	33.0	22.4	27.5	15.8	3.8	2.6
2.50	None	83.8	64.5	79.6	62.7	80.2	68.7	63.7	31.7
	T	80.2	59.3	75.1	56.7	75.3	61.8	49.2	11.1
	Q	75.5	60.6	70.5	57.6	70.6	60.6	52.0	28.6
	P	72.7	52.7	66.5	49.3	65.6	51.3	30.5	5.6
	Q,T	70.4	54.2	64.0	50.0	63.6	51.2	34.2	9.6
	Q,P	65.7	49.4	59.4	44.4	58.2	45.1	22.2	5.6
	Pex	56.1	40.7	50.6	36.2	48.0	35.9	15.8	4.1
	Q,Pex	51.0	37.4	44.7	31.9	41.9	29.9	11.4	4.1
3.00	None	88.0	69.2	84.8	68.1	85.7	75.3	74.6	46.5
	T	85.4	64.0	81.6	62.5	82.2	69.7	64.6	25.2
	Q	81.3	65.9	77.4	64.0	78.2	68.7	65.4	43.5
	P	79.0	58.3	74.3	55.8	74.6	61.0	50.3	10.9
	Q,T	76.8	60.1	72.2	57.2	72.5	61.3	52.9	21.7
	Q,P	72.6	55.0	67.8	51.3	68.0	55.3	42.5	10.6
	Pex	63.2	45.7	58.5	42.6	57.3	44.7	32.6	7.3
	Q,Pex	58.0	42.8	52.6	38.4	51.4	39.2	26.0	6.3

Table 37: Children and teenager targeted pre-pandemic vaccination on infection rates, regionally mitigated, 60 percent compliance

For Ferguson-like disease manifestation and implementation threshold when 10 cases are diagnosed. Case-based strategy combinations vertical, network-focused strategy combinations horizontal. Green shading, infection rates 10 percent or less; pink shading, infection rates between 10 and 25 percent.

S - Schools closed	CTsd - Child/Teen social distancing	ASsd - Adult/Senior social distancing	Q - Household Quantine	T - Antiviral Treatment	P - Antiviral Prophylaxis	PEx - Extended Antiviral Prophylaxis

ID Factor		None	ASsd	CTsd	CTsd,ASsd	S	S, ASsd	S,CTsd	,CTsd,ASs
0.75	None	15.9	13.6	12.4	9.6	1.7	1.6	1.3	1.5
	T	5.5	4.6	4.3	2.8	1.2	1.4	1.2	1.1
	Q	9.2	8.4	5.8	6.6	1.5	1.4	1.3	1.3
	P	3.0	2.6	2.7	2.2	1.2	1.2	1.2	1.2
	Q,T	3.7	2.6	2.9	2.5	1.3	1.4	1.2	1.4
	Q,P	2.5	2.1	1.9	1.9	1.3	1.2	1.1	1.2
	Pex	1.9	1.8	1.7	1.8	1.1	1.3	1.1	1.3
	Q,Pex	1.4	1.6	1.6	1.6	1.0	1.5	1.2	0.0
1.00	None	40.5	36.6	39.1	34.6	12.5	8.4	2.9	2.1
	T	30.8	27.6	27.5	23.5	3.7	2.9	1.7	1.6
	Q	34.8	33.4	33.7	31.1	6.8	4.5	2.6	2.4
	P	21.9	19.4	18.2	13.6	2.2	2.1	1.4	1.4
	Q,T	23.0	22.6	22.1	18.7	2.4	2.0	1.6	1.6
	Q,P	18.3	16.0	15.1	11.3	2.2	2.0	1.4	1.3
	Pex	10.1	8.0	6.9	5.6	1.9	1.6	1.4	1.4
	Q,Pex	7.3	5.8	5.5	4.2	1.5	1.6	1.5	1.4
1.25	None	54.7	49.7	53.8	48.4	40.1	33.8	19.6	11.3
	T	47.1	41.9	45.0	39.8	24.9	16.3	4.6	3.6
	Q	49.5	47.1	48.8	46.0	30.9	27.7	15.8	11.4
	P	39.5	35.0	36.8	32.1	12.3	7.5	2.8	2.1
	Q,T	41.2	38.0	39.8	35.7	14.8	10.8	4.0	2.9
	Q,P	34.8	32.4	33.1	29.3	7.6	5.2	2.6	2.1
	Pex	25.7	22.2	23.5	19.3	4.6	3.6	2.0	1.8
	Q,Pex	22.8	20.5	20.5	16.0	3.5	3.1	1.8	1.8
1.50	None	65.1	58.9	63.6	58.0	55.8	49.8	42.2	32.6
	T	58.1	51.6	56.6	50.2	44.8	37.8	23.2	11.9
	Q	59.8	56.9	59.4	55.8	49.6	44.9	38.1	32.1
	P	50.9	44.9	48.8	43.1	33.3	26.2	9.7	4.5
	Q,T	52.3	48.8	51.3	47.7	36.4	30.8	19.2	11.2
	Q,P	46.6	42.8	45.1	40.9	26.8	19.9	7.9	4.6
	Pex	35.4	31.1	33.8	29.4	18.8	11.8	5.2	3.0
	Q,Pex	32.5	29.2	30.7	27.0	13.4	9.1	3.6	3.0
2.00	None	77.1	70.3	76.1	70.0	73.4	68.5	65.9	58.1
	T	72.2	64.4	70.6	63.7	66.9	60.3	54.7	43.7
	Q	72.9	69.5	72.4	69.0	68.6	65.3	62.9	57.9
	P	65.5	58.1	63.8	57.0	57.8	50.9	43.2	30.3
	Q,T	66.9	62.8	66.3	62.0	60.3	55.9	50.7	43.7
	Q,P	61.2	56.5	60.5	55.6	53.0	47.3	39.3	30.0
	Pex	48.6	42.8	47.6	41.5	39.9	34.3	25.0	14.9
	Q,Pex	45.5	41.4	44.8	40.0	36.2	31.0	23.0	15.0
2.50	None	84.2	77.8	83.0	77.6	82.5	78.2	77.8	71.6
	T	80.4	72.6	79.1	72.2	77.8	72.1	70.5	61.5
	Q	80.7	77.3	80.4	77.0	78.9	76.1	75.2	71.2
	P	74.7	66.9	73.4	66.3	71.0	64.7	61.5	50.9
	Q,T	76.1	71.6	75.3	71.2	73.0	68.7	67.1	61.1
	Q,P	71.0	65.8	70.1	65.2	66.8	61.7	58.9	50.7
	Pex	57.8	50.9	56.8	50.3	53.3	46.8	42.8	32.9
	Q,Pex	54.7	49.8	54.1	48.9	49.5	44.2	40.5	32.7
3.00	None	88.4	82.9	87.6	82.6	87.5	84.0	84.6	79.3
	T	85.6	78.3	84.3	78.0	84.1	79.4	79.5	72.0
	Q	85.8	82.5	85.2	82.3	84.7	82.3	82.4	79.4
	P	81.0	73.1	79.8	72.8	78.8	73.3	72.6	63.6
	Q,T	81.8	77.6	81.3	77.5	80.2	76.9	76.4	71.6
	Q,P	77.7	72.4	76.9	72.3	75.3	71.1	70.0	63.1
	Pex	64.8	57.1	63.8	56.9	61.9	55.7	53.9	44.5
	Q,Pex	62.0	56.2	61.3	55.8	58.5	53.5	51.7	44.0

Table 38: Children and teenager targeted pre-pandemic vaccination on infection rates, local-only mitigation, 90 percent compliance

For Ferguson-like disease manifestation, implementation when 10 cases are diagnosed, and 700 doses of 50-percent efficacy pre-pandemic vaccine is distributed among 2900 children and teens. Case-based strategy combinations vertical, network-focused strategy combinations horizontal with green shading, infection attack rate 10 percent or less; pink shading, infection attack rate between 10 and 25 percent.

S - Schools closed	CTsd - Child/Teen social distancing	ASsd - Adult/Senior social distancing	Q - Household Quantine	T - Antiviral Treatment	P - Antiviral Prophylaxis	PEx - Extended Antiviral Prophylaxis

ID Factor		None	ASsd	CTsd	CTsd,ASsd	S	S, ASsd	S,CTsd	,CTsd,ASs
0.75	None	17.0	8.8	8.9	4.5	4.5	2.8	2.8	2.0
	T	9.8	4.6	5.4	2.8	3.4	2.1	2.3	1.7
	Q	8.3	4.0	5.4	3.0	3.7	2.4	2.8	2.1
	P	6.3	3.1	4.1	2.4	2.8	2.0	2.2	1.8
	Q,T	5.6	2.8	3.7	2.4	3.0	2.0	2.3	1.7
	Q,P	5.2	2.7	3.6	2.2	2.8	1.9	2.2	1.7
	Pex	4.6	2.7	3.6	2.3	2.7	1.9	2.2	1.7
	Q,Pex	3.9	2.3	3.1	2.1	2.6	1.9	2.1	1.7
1.00	None	40.7	28.8	30.1	20.7	19.8	11.2	8.4	4.1
	T	32.9	22.3	22.2	13.2	13.6	6.9	6.6	3.4
	Q	29.3	19.1	19.8	11.6	13.5	7.4	7.8	4.1
	P	25.5	15.6	15.8	7.8	9.8	5.1	5.9	3.2
	Q,T	23.5	13.4	14.4	7.5	10.1	5.3	6.3	3.4
	Q,P	20.9	11.3	12.7	6.4	8.8	4.7	5.8	3.1
	Pex	16.7	9.2	11.4	6.1	8.0	4.4	5.4	3.1
	Q,Pex	13.9	7.2	9.8	5.1	7.2	4.2	5.3	3.0
1.25	None	54.7	41.1	46.6	34.6	39.2	27.2	17.0	7.8
	T	48.4	34.9	38.9	26.7	30.2	18.2	12.8	5.9
	Q	44.5	33.2	35.6	25.3	28.8	18.5	15.8	7.8
	P	40.4	28.2	30.8	19.5	21.9	11.9	10.8	5.3
	Q,T	38.2	26.9	28.4	18.4	21.9	12.2	12.1	6.0
	Q,P	34.8	23.5	25.2	15.0	18.9	10.3	10.7	5.3
	Pex	27.8	18.8	20.6	12.9	15.2	8.7	9.4	4.9
	Q,Pex	23.8	15.4	17.9	10.2	13.7	7.9	9.3	5.0
1.50	None	64.6	48.8	57.7	44.5	54.0	41.5	28.6	13.7
	T	59.1	43.4	51.0	38.0	45.6	32.1	21.5	9.7
	Q	54.8	42.8	47.9	37.0	43.2	31.8	26.0	13.5
	P	51.0	37.3	42.6	30.1	35.4	22.5	17.8	8.3
	Q,T	49.1	36.8	41.0	29.7	34.9	23.0	19.8	9.9
	Q,P	45.4	33.0	36.9	25.1	30.7	18.8	16.9	8.2
	Pex	36.9	26.5	30.0	20.5	24.0	15.6	14.5	7.5
	Q,Pex	32.8	23.2	26.2	16.9	21.6	13.4	14.1	7.3
2.00	None	76.6	58.7	71.6	56.3	71.4	58.8	50.6	27.8
	T	72.6	54.2	66.7	50.9	65.3	51.7	40.6	19.1
	Q	68.8	55.3	63.8	51.5	62.1	51.0	45.8	27.5
	P	65.2	48.7	58.9	44.3	56.0	42.2	33.0	15.4
	Q,T	63.3	49.5	57.6	44.6	55.1	42.6	36.4	19.1
	Q,P	59.0	45.1	53.1	39.7	49.6	37.0	31.2	15.3
	Pex	50.2	37.1	44.2	32.5	39.5	28.3	24.1	12.8
	Q,Pex	45.5	33.8	39.5	28.7	35.1	24.7	23.2	12.6
2.50	None	83.6	65.2	79.9	63.9	80.5	69.0	66.2	41.8
	T	80.7	60.9	76.2	59.0	76.2	63.2	57.2	30.8
	Q	77.2	63.0	73.4	60.6	73.2	62.7	61.0	41.7
	P	74.2	56.0	69.4	53.3	68.4	54.9	47.7	24.3
	Q,T	72.4	57.5	68.3	54.3	67.5	55.5	51.9	30.5
	Q,P	68.2	53.2	63.6	49.4	62.5	50.0	45.3	24.1
	Pex	59.4	44.6	54.2	41.0	51.2	39.6	33.6	18.7
	Q,Pex	54.8	41.6	49.5	37.2	46.5	35.4	32.4	18.7
3.00	None	87.9	69.6	84.9	68.9	85.8	75.6	75.6	52.5
	T	85.6	65.6	81.9	64.3	82.7	71.0	68.6	41.1
	Q	82.5	68.1	79.6	66.6	80.1	70.5	71.0	52.4
	P	80.3	61.3	76.3	59.5	76.4	63.6	59.4	33.3
	Q,T	78.5	63.2	75.2	61.2	75.1	64.1	63.2	41.0
	Q,P	74.6	59.0	71.1	56.2	70.9	59.2	56.3	33.1
	Pex	66.1	50.1	61.7	47.3	59.9	47.7	42.7	24.3
	Q,Pex	61.5	47.3	57.5	43.9	55.3	43.8	40.6	24.6

Table 39: Children and teenager targeted pre-pandemic vaccination on infection rates, regionally unmitigated, 60 percent compliance

For Ferguson-like disease manifestation, implementation threshold when 10 cases are diagnosed, and 700 doses of 50-percent efficacy pre-pandemic vaccine is distributed among 2900 children and teens. Case-based strategy combinations vertical, network-focused strategy combinations horizontal with green shading, infection rate 10 percent or less; pink shading, infection rate between 10 and 25 percent.

S - Schools closed	CTsd - Child/Teen social distancing	ASsd - Adult/Senior social distancing	Q - Household Quantine	T - Antiviral Treatment	P - Antiviral Prophylaxis	PEx - Extended Antiviral Prophylaxis

ID Factor		None	ASsd	CTsd	CTsd,ASsc	S	S, ASsd	S,CTsd	,CTsd,ASs
0.75	None	18.1	15.7	16.1	12.0	4.7	3.2	3.5	2.6
	T	9.7	7.3	8.4	5.5	3.6	2.4	2.9	2.0
	Q	13.2	10.6	12.3	8.7	4.4	3.0	3.5	2.4
	P	6.7	4.6	6.0	3.6	3.1	2.2	2.6	2.0
	Q,T	7.2	4.5	6.7	4.4	3.2	2.3	2.8	2.0
	Q,P	5.6	3.4	5.1	3.3	3.0	2.2	2.6	1.9
	Pex	4.5	2.9	4.4	2.9	3.0	2.2	2.5	1.9
	Q,Pex	4.1	2.9	3.9	2.7	2.8	2.1	2.5	1.9
1.00	None	40.8	36.5	39.5	34.7	21.7	15.1	13.3	7.8
	T	33.0	28.7	31.1	25.7	15.1	9.6	9.5	5.6
	Q	36.2	33.3	35.5	31.7	17.8	12.4	12.9	8.0
	P	27.6	22.5	25.0	19.0	11.5	7.1	8.0	4.7
	Q,T	27.8	24.7	26.9	21.6	12.4	8.0	9.2	5.6
	Q,P	23.9	19.5	21.7	16.4	10.6	6.5	8.0	4.8
	Pex	17.6	13.7	16.2	11.6	9.2	5.8	7.1	4.4
	Q,Pex	15.7	11.3	14.7	9.6	8.6	5.4	7.0	4.3
1.25	None	54.7	49.3	53.5	48.0	41.9	34.4	28.9	20.0
	T	48.2	42.4	46.4	40.3	32.8	24.6	20.8	13.2
	Q	50.6	46.9	50.0	45.6	36.7	30.3	28.0	20.6
	P	42.5	36.4	40.5	34.4	25.5	17.7	17.0	10.3
	Q,T	43.4	39.2	42.9	37.6	27.7	20.3	20.2	12.6
	Q,P	38.9	33.9	37.2	31.7	23.1	15.6	16.6	10.0
	Pex	29.3	25.1	28.1	22.4	17.9	12.1	13.2	8.4
	Q,Pex	26.9	22.5	25.8	20.8	16.6	11.1	13.0	8.5
1.50	None	64.6	58.2	63.4	57.0	56.5	49.5	45.3	35.2
	T	58.9	52.1	57.5	50.8	48.4	39.8	35.4	24.8
	Q	60.4	56.2	60.0	55.6	51.3	45.0	43.9	35.6
	P	52.9	46.3	51.1	44.3	40.6	31.8	28.8	19.0
	Q,T	54.2	49.4	53.7	48.2	42.8	35.3	33.8	24.4
	Q,P	49.5	44.0	48.3	42.5	36.9	28.4	27.7	19.0
	Pex	38.9	33.2	37.6	31.4	28.0	20.9	20.9	13.6
	Q,Pex	36.4	31.4	35.5	29.9	25.8	19.4	20.4	13.6
2.00	None	76.8	69.4	75.7	69.0	73.3	67.2	66.3	57.4
	T	72.6	64.4	71.3	63.6	67.7	60.1	58.1	46.9
	Q	73.3	68.6	72.8	68.4	69.2	64.3	64.2	57.3
	P	67.1	58.9	65.8	58.1	60.7	52.9	50.3	37.8
	Q,T	68.4	62.9	67.9	62.5	62.5	56.5	56.1	46.8
	Q,P	63.8	57.7	63.0	56.8	56.8	49.5	48.9	38.3
	Pex	52.1	45.0	50.6	43.9	44.0	36.9	35.3	26.1
	Q,Pex	49.1	43.5	48.6	42.7	41.1	34.8	34.3	26.0
2.50	None	83.8	76.9	82.9	76.7	82.2	77.2	77.7	70.2
	T	80.7	72.4	79.3	72.0	78.1	71.9	71.8	62.2
	Q	80.9	76.5	80.6	76.2	79.1	74.9	76.1	70.4
	P	76.0	67.7	74.7	67.0	72.4	65.4	65.0	53.9
	Q,T	77.1	71.5	76.5	71.2	74.0	68.7	69.6	62.2
	Q,P	72.8	66.6	72.3	66.3	69.0	62.8	63.2	54.3
	Pex	61.2	53.1	59.9	52.5	55.6	48.6	47.4	37.7
	Q,Pex	58.5	51.9	57.9	51.2	52.8	46.2	46.4	37.4
3.00	None	88.0	81.7	87.2	81.8	87.2	83.0	84.2	78.2
	T	85.6	77.8	84.5	77.5	84.2	79.0	80.0	71.7
	Q	85.8	81.6	85.2	81.5	84.7	81.5	82.7	78.2
	P	81.9	73.6	80.7	73.4	79.8	73.7	74.2	64.8
	Q,T	82.6	77.5	82.1	77.2	80.9	76.6	77.8	71.6
	Q,P	79.0	73.0	78.4	72.7	76.8	71.6	72.6	64.8
	Pex	67.8	59.5	66.9	58.9	63.8	56.9	56.7	47.2
	Q,Pex	65.4	58.4	64.8	58.1	61.2	54.8	55.5	47.2

Appendix A: Unmitigated Base Case Analysis

Analysis of the unmitigated epidemics for the base infectious contact network with the Ferguson-like and Longini-like disease manifestations is compiled in the excel workbook:

http://www.sandia.gov/nisac/docs/PLoSOne-SIFiles/UnmitigatedBaseCase.xls

The first worksheet of the Excel workbook contains a stand-alone description of the simulation set and what is contained within the workbook. The next worksheet gives summary statistics for:

1. Infection attack rates, illness attack rates, death rates by age class and overall
2. Peak infected, peak symptomatic
3. Number of epidemics
4. Timescales: times to peak, epidemic duration, total time of effects
5. Days adults are home

This is followed by a set of 9 worksheets where the infectious contact sequences generated from 100 runs are analyzed to obtain:

6. Branching factors by age class and generation
7. Maximum branching factors by age class and overall
8. Overall and average branching factor for combined population of 1M people by generation
9. Maximum overall branching factor below generation 10 for combined population of 1M people (approximates R_o) [13]
10. Generation time by age class and generation
11. Generation time by age class (averaged over generation)
12. Average generation time over all age classes
13. Fraction of total transmission within and between each age class
14. Fraction of total transmission within each group type

The Ferguson-like disease manifestation with the base infectious contact network was analyzed in "Targeted Social Distancing Design for Pandemic Influenza" [13], and results here conform closely to those with slight differences due to the inclusion of babysitting within the community. We list summary observations below on the influence of 1) disease manifestation, 2) I_F, and 3) compliance.

Observations on Disease Manifestation (comparing Ferguson-like to Longini-like):

- There is no difference in the number of total infected (or symptomatic) by age class or overall between the two disease manifestations.
- The Longini-like manifestation demonstrates slightly higher numbers of peak infected and peak symptomatic.
- The Longini-like manifestation shows an approximately 33-percent longer time scale. Times to peak, epidemic duration, and total time of epidemic effects are longer than the Ferguson-like manifestation.
- The Longini-like manifestation results in approximately 50-percent more adult days out than the Ferguson-like.

See plots below (**Figure A-1 through A-3**)

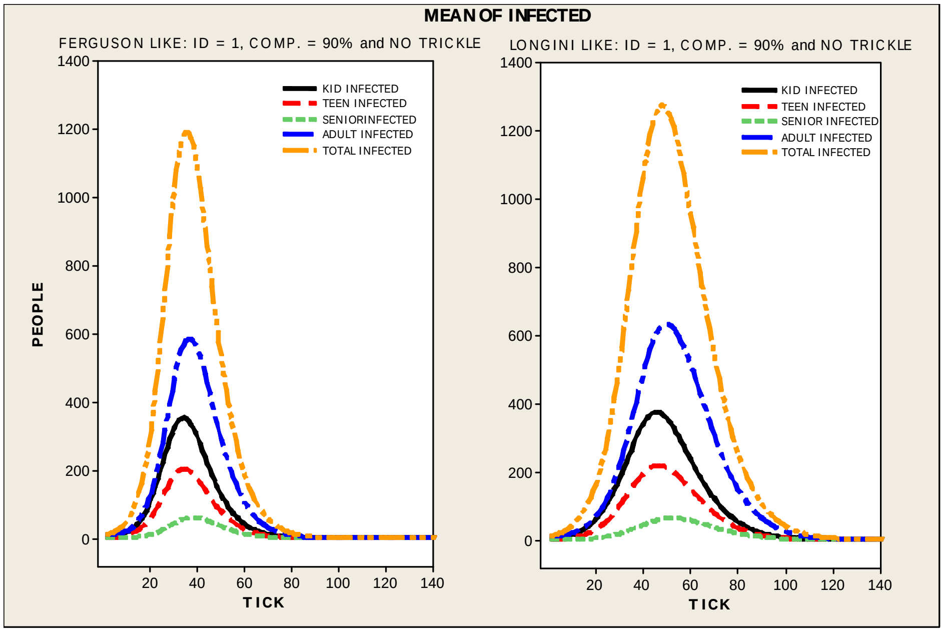

Figure A-1: Epidemic curves for infection by age class and total population (averages of 100 simulations) for Ferguson-like (left) vs Longini-like (right) disease manifestations. Tick refers to day of epidemic.

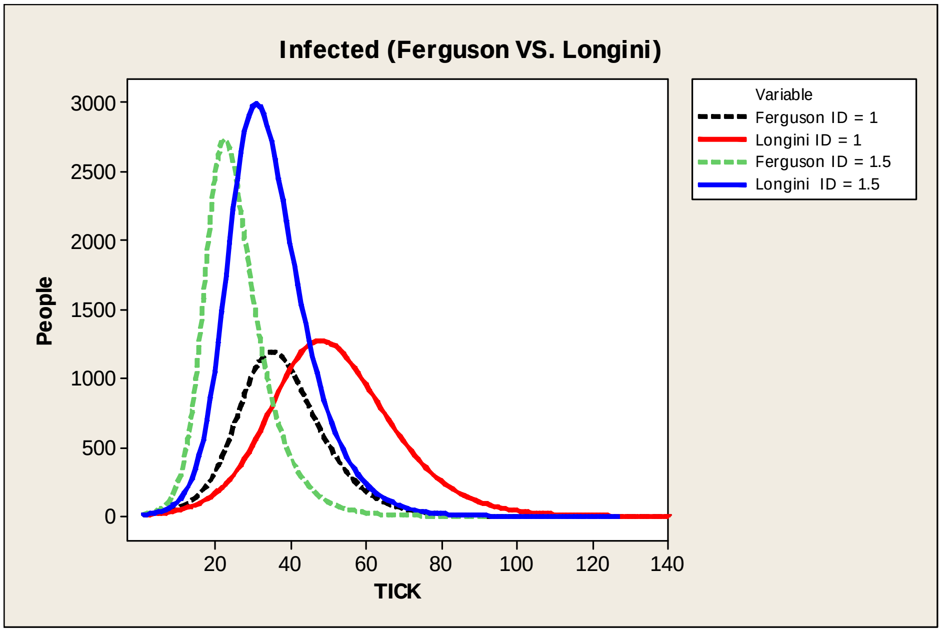

Figure A-2: Epidemic curves for numbers of individuals infected in population of 10,000 for Ferguson-like and Longini-like disease manifestation at I_F1.0 and 1.5 (averages of 100 simulations). Tick refers to day of epidemic.

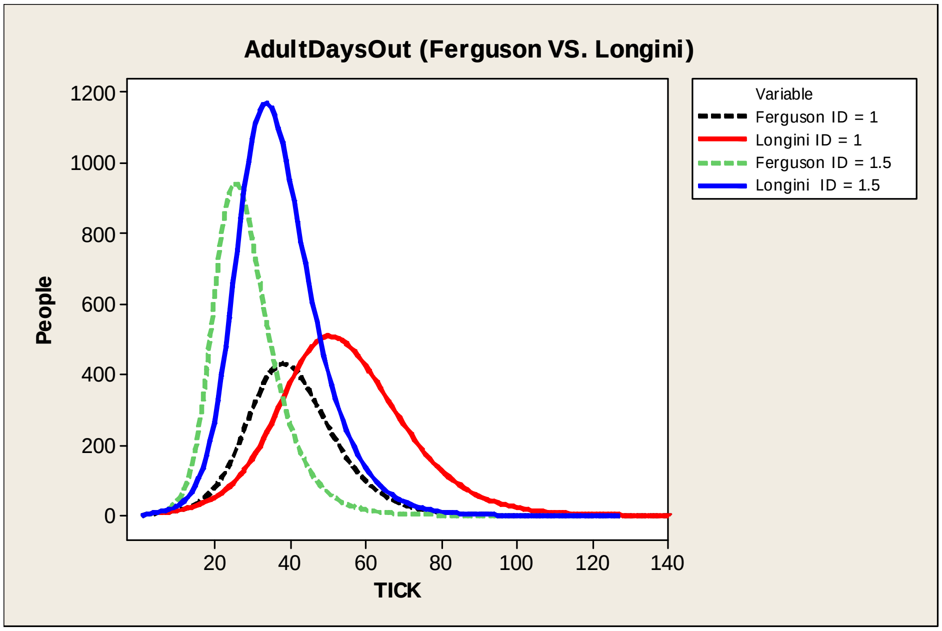

Figure A-3: Number of adult days at home by day for Ferguson-like and Longini-like disease manifestations at I_F 1.0 and 1.5 (averages of 100 simulations). Tick refers to day of epidemic.

Observations on I_F for Ferguson- and Longini-like disease manifestations:

- Increasing I_F increases the infection rates, peak values for infected and symptomatic, and the number of days adults are at home, while decreasing the times to peak and total time of the epidemic.
- Increasing I_F also shifts the *from-to* contact fractions toward adults and the infectious context fraction from household and school to neighborhood and work. These shifts occur because the branching factors for adults are pushed above 1 as I_F increases and so adults become a primary substrate for transmission over children and teens.

See plots below (**Figures A-4 through A-6**).

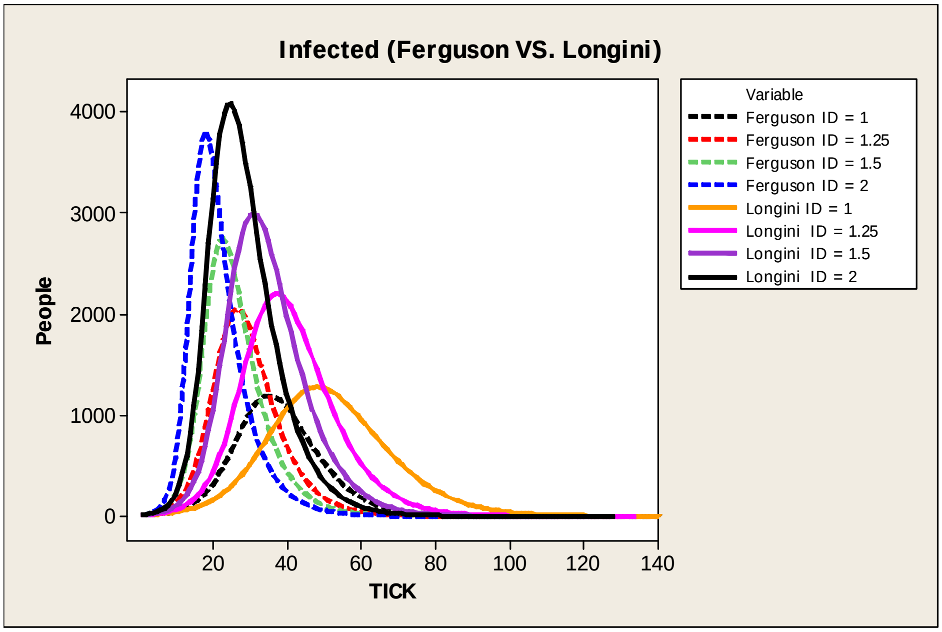

Figure A-4: Epidemic curves for number of infected individuals by I_F for Ferguson-like and Longini-like disease manifestations.

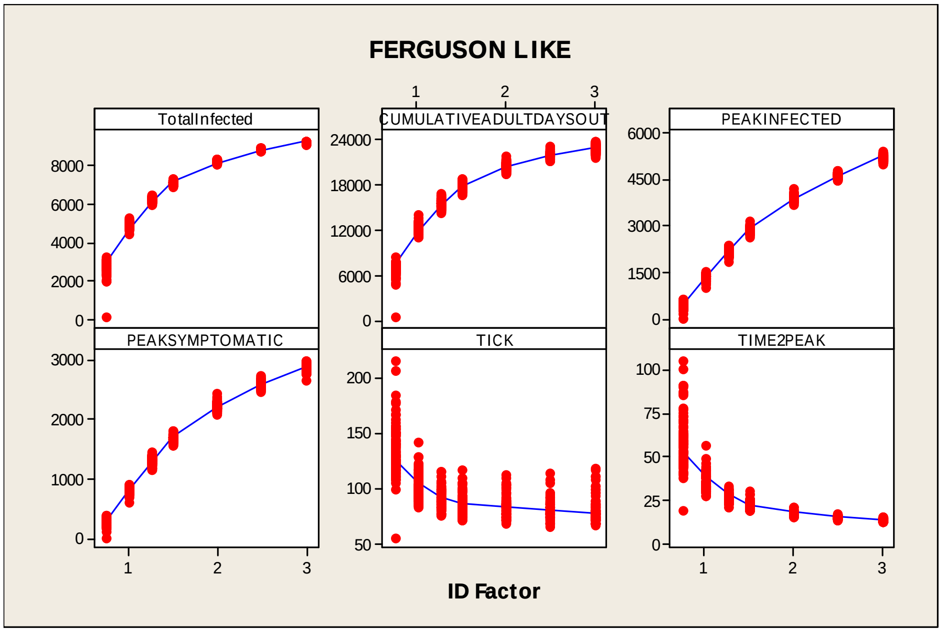

Figure A-5: Results for 100 simulations (Ferguson-like disease manifestation) on outcome measures of total infected, adult days out, peak infected, peak symptomatic, length of epidemic (tick), and time to peak infections by I_F. Each point represents the results of a single simulation.

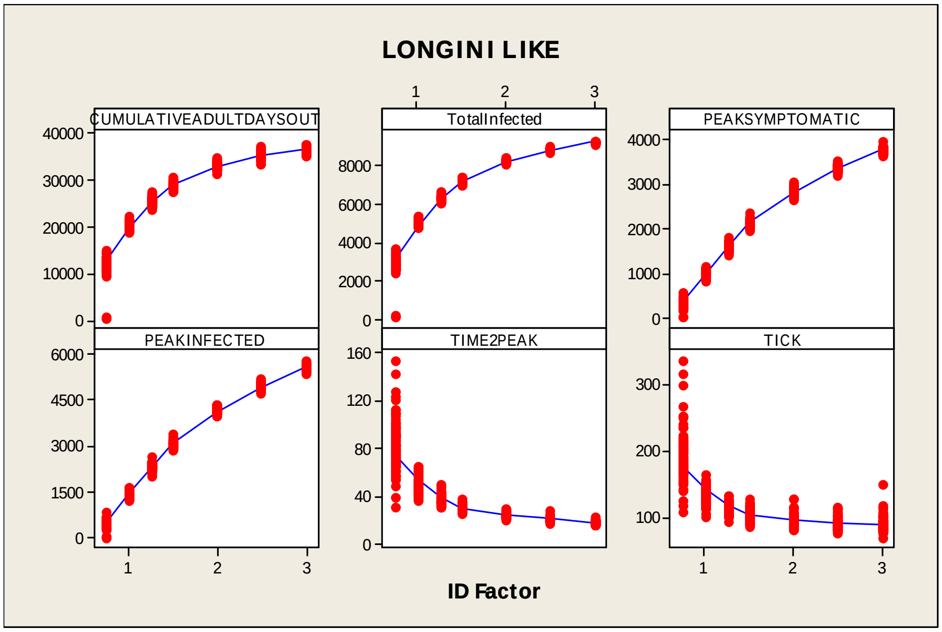

Figure A-6: Results for 100 simulations (Longini-like disease manifestation) on outcome measures of total infected, adult days out, peak infected, peak symptomatic, length of epidemic (tick), and time to peak infections by I_F. Each point represents the results of a single simulation.

Observations on 90-percent and 60-percent compliance for Ferguson- and Longini-like disease manifestations:

- Compliance has essentially no influence on results once I_F has been tuned to a 50-percent infection attack rate except that a change from 90-percent to 60-percent compliance shifts infectious contact fraction a bit (approximately 3–5 percent) from household to non-household contexts. This is consistent with the increase in non-household contacts at 60 percent compliance.

See plot below (**Figure A-7**).

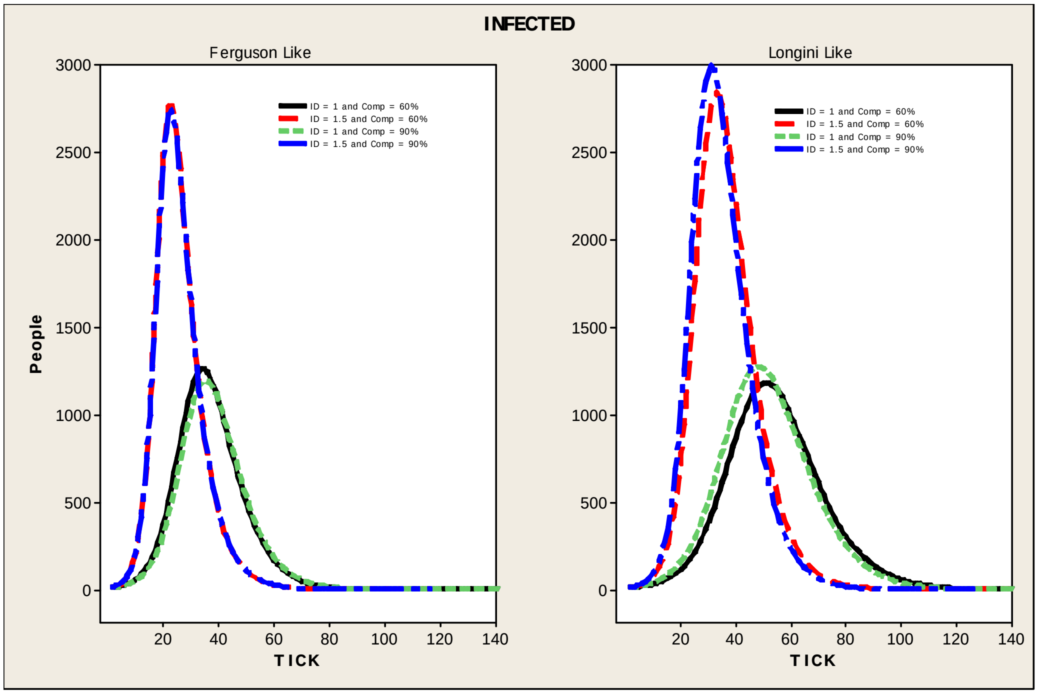

Figure A-7: Epidemic curves for infection rates for Ferguson-like (left) and Longini-like (right) disease manifestations by I_F and level of compliance (60 or 90%). Tick refers to day of epidemic.

Appendix B: Core Strategy Matrix Results

Statistical measures over 100 runs for the core matrix of mitigation strategies are compiled in 2 Excel workbooks separated as averages and standard deviations (*SD*):

http://www.sandia.gov/nisac/docs/PLoSOne-SIFiles/FergusonEpidemicCases.xls
http://www.sandia.gov/nisac/docs/PLoSOne-SIFiles/FergusonEpidemicCases-SD.xls

The first worksheet of each Excel workbook contains a stand-alone description of the simulation matrix and what is contained within the worksheet. Subsequent worksheets create a set of tables with 3-dimensional (3D) bar graphs below for the following 15 measures:

1. Number of simulations that yield epidemics (defined as greater than 1 percent of population infected)
2. Infection rate
3. Illness rate
4. Deaths
5. Peak infected
6. Time to peak infected
7. Peak symptomatic
8. Time to peak symptomatic
9. Epidemic duration (from first 10 diagnosed to last diagnosed)
10. Total time of effects (from initial seeding to last person recovered)
11. Number of days strategies imposed
12. Number of mitigation cycles
13. Number of external infections
14. Number of antiviral courses given
15. Number of days adults are at home (either sick, quarantined, or tending sick children or children sent home from school)

In each table and 3D bar graph, strategies were organized with network-focused interventions of S, CTsd, and ASsd in columns and case-based interventions Q, T, P, and PEx in rows, yielding the 64 possible combinations at each of 7 I_F. To aid in viewing these data, those strategies that yield an attack rate that is 10 percent or less are shaded green and those up from 10 to 25 percent are shaded pink in the tables.

Time series plots for daily measures averaged over the set of 100 simulations may be made for any of the 64 combinations of interventions at 7 I_F, 2 levels of compliance, or 2 boundary conditions. An example set of such time series plots for I_F of 1.5 and Ferguson-like disease manifestation that consider the measures of people infected, given antiviral, adults at home, and symptomatic are presented in the following files:

http://www.sandia.gov/nisac/docs/PLoSOne-SIFiles/Sequence1.5-90.pdf
http://www.sandia.gov/nisac/docs/PLoSOne-SIFiles/Sequence1.5-60.pdf
http://www.sandia.gov/nisac/docs/PLoSOne-SIFiles/Sequence1.5-90ExternalBaseEpidemic.pdf
http://www.sandia.gov/nisac/docs/PLoSOne-SIFiles/Sequence1.5-60ExternalBaseEpidemic.pdf

Appendix C: Matrix Perturbation and Extension Results

We considered the following perturbations and extensions to test the robustness of the core mitigation strategy matrix:

Perturbations

1. 2 delayed implementation thresholds (day after 30 or 100 cases diagnosed within the community)
2. 1 relaxed rescinding threshold (3 cases/7 days and if epidemic recurs (10 additional cases), strategies are re-implemented)

Extensions

3. 2 alterations in natural history of disease manifestation
   - Longini-like [22,23]
   - Longini-like with an extended (7-day) infectious recovery period
4. Similar transmission within children, teens, and adults (uniform relative infectivity and susceptibility and identical number of contacts within workplaces and schools)
5. Augmented contact network
6. 3 pre-pandemic vaccination interventions with 7-percent coverage of 50-percent efficacy vaccine:
   - 1) administered randomly
   - 2) targeted to children and teens or
   - 3) targeted to adults

For each extension, we conducted 100 runs for the full set of 64 containment intervention combinations, 7 I_F, 2 compliances (90 percent, 60 percent) and 2 boundary conditions (regional or local-only mitigation). Statistical measures over 100 runs are complied in Excel workbooks, separated as averages and standard deviations.

Excel workbooks for implementation threshold relaxed to day after 30 or 100 cases diagnosed within the community:

http://www.sandia.gov/nisac/docs/PLoSOne-SIFiles/FergusonT1EpidemicCases.xls
http://www.sandia.gov/nisac/docs/PLoSOne-SIFiles/FergusonT1EpidemicCases-SD.xls
http://www.sandia.gov/nisac/docs/PLoSOne-SIFiles/FergusonT2EpidemicCases.xls
http://www.sandia.gov/nisac/docs/PLoSOne-SIFiles/FergusonT2EpidemicCases-SD.xls

Excel workbooks for rescinding threshold:

http://www.sandia.gov/nisac/docs/PLoSOne-SIFiles/FergusonRescind3EpidemicCases.xls
http://www.sandia.gov/nisac/docs/PLoSOne-SIFiles/FergusonRescind3EpidemicCases-SD.xls

Excel workbooks for Longini-like disease manifestation:

http://www.sandia.gov/nisac/docs/PLoSOne-SIFiles/LonginiEpidemicCases.xls
http://www.sandia.gov/nisac/docs/PLoSOne-SIFiles/LonginiEpidemicCases-SD.xls

Excel workbooks for Longini-like with an extended (7-day) infectious recovery period:

http://www.sandia.gov/nisac/docs/PLoSOne-SIFiles/LonginiExtShedEpidemicCases.xls
http://www.sandia.gov/nisac/docs/PLoSOne-SIFiles/LonginiExtShedEpidemicCases-SD.xls

Excel workbooks for similar transmission within children, teenagers and adults:

http://www.sandia.gov/nisac/docs/PLoSOne-SIFiles/FergusonU1EpidemicCases.xls
http://www.sandia.gov/nisac/docs/PLoSOne-SIFiles/FergusonU1EpidemicCases-SD.xls

Excel workbooks for augmented social network:

http://www.sandia.gov/nisac/docs/PLoSOne-SIFiles/FergusonAugNet1EpidemicCases.xls
http://www.sandia.gov/nisac/docs/PLoSOne-SIFiles/FergusonAugNet1EpidemicCases-SD.xls

Excel workbooks for pre-pandemic vaccination (V1=distributed randomly to all age classes; V2=distributed to children and teenagers; V3=distributed to adults):

http://www.sandia.gov/nisac/docs/PLoSOne-SIFiles/FergusonV1EpidemicCases.xls
http://www.sandia.gov/nisac/docs/PLoSOne-SIFiles/FergusonV1EpidemicCases-SD.xls
http://www.sandia.gov/nisac/docs/PLoSOne-SIFiles/FergusonV2EpidemicCases.xls
http://www.sandia.gov/nisac/docs/PLoSOne-SIFiles/FergusonV2EpidemicCases-SD.xls
http://www.sandia.gov/nisac/docs/PLoSOne-SIFiles/FergusonV3EpidemicCases.xls
http://www.sandia.gov/nisac/docs/PLoSOne-SIFiles/FergusonV3EpidemicCases-SD.xls

As in **Appendices A and B**, the first worksheet of each Excel workbook contains a stand-alone description of the simulation matrix and what is contained within the workbook. Subsequent worksheets create a set of tables and 3D bar graphs for the following 15 measures:

1. Number of simulations that yield epidemics (defined as greater than 1 percent of population infected)
2. Infection rate
3. Illness rate
4. Deaths
5. Peak infected
6. Time to peak infected
7. Peak symptomatic
8. Time to peak symptomatic
9. Epidemic duration (from implementation threshold to last diagnosed)
10. Total time of effects (from initial seeding to last person recovered)
11. Number of days strategies imposed
12. Number of mitigation cycles
13. Number of external infections
14. Number of antiviral courses given
15. Number of days adults are at home (either sick, quarantined, or tending sick children or children sent home from school)

In each table and 3D bar graph, we organized strategies with network-focused interventions of S, CTsd, and ASsd in columns and case-based interventions of Q, T, P, and PEx in rows, yielding the 64 possible strategies at each of 7 I_F. To aid in viewing these data, those combinations that yield an infection rate that is 10 percent or less are shaded green and those that yield an infection rate from 10 to 25 percent are shaded pink in the tables. Time series plots for daily measures averaged over the set of 100 simulations may be made for any of the combinations of strategies (64), I_F (7), compliance (2), or boundary condition (2), for any of the perturbations or extensions.

References

1. Glass RJ, Beyeler W, Stamber K, Glass L, LaViolette R, et al. (2005) Simulation and analysis of cascading failure in critical infrastructure. SAND2005-2877 C. National Infrastructure & Analysis Center, Sandia National Laboratories. Available: http://www.sandia.gov/nisac/docs/Glass_annotatedpresentation.pdf Accessed 11 April 2008.

2. Feigenbaum J (2003) Financial physics. Rep Prog Phys 66: 1611-1649 doi:10.1088/0034-4885/66/10/R02

3. Goldstone RL, Janssen MA (2005) Computational models of collective behavior. Trends Cog Sci 9: 425-430.

4. Eubank S, Guclu H, Kumar VSA, Marathe MV, Srinivasan A, et al. (2004) Modelling disease outbreaks in realistic urban social networks. Nature 429: 180-184.

5. Barthelemy M, Barrat A, Pastor-Satorras R, Vespignani A (2005) Dynamical patterns of epidemic outbreaks in complex heterogeneous networks. J Theor Biol 235: 275-288.

6. Newman MEJ (2002) Spread of epidemic disease on networks. Phys Rev E Stat Nonlin Soft Matter Phys 66: 1-11.

7. Laviolette RA, Beyeler W, Glass RJ, Stamber KL, Link H (2006) Sensitivity of the resilience of congested random networks to rolloff and offset in truncated power-law degree distributions. Physica A 368: 287-293.

8. Glass RJ, Beyeler WE, Stamber KL (2004) Advanced simulation for analysis of critical infrastructure: abstract cascades, the electrical power grid, and Fedwire. SAND2004-4239. National Infrastructure Simulation and Analysis Center, Sandia National Laboratories. Available: http://www.prod.sandia.gov/cgi-bin/techlib/access-control.pl/2004/044239.pdf Accessed 11 April 2008.

9. Beyeler W, Glass RJ, Bech M, Soramaki K (2007) Congestion and cascades in payment systems. Physica A 384: 693-718.

10. Soramaki K, Bech ML, Arnold J, Glass RJ, W.E. B (2007) The topology of interbank payment flows. Physica A 379: 317-333.
.
11. Backus GA, Glass RJ (2006) An agent-based model component to a framework for the analysis of terrorist group dynamics. SAND2006-0860P. National Infrastructure & Analysis Center, Sandia National Laboratories. Available: https://cfwebprod.sandia.gov/cfdocs/CCIM/docs/06_0860P_ConceptualModel1.pdf Accessed 11 April 2008.

12. Glass RJ, Glass LM, Beyeler W (2005) Local mitigation strategies for pandemic influenza. SAND2005-7955J. National Infrastructure & Analysis Center, Sandia National Laboratories.

Available: http://www.sandia.gov/nisac/docs/NISAC_FluMitigationPaperWithFullSOMTables.doc Accessed 11 April 2008.

13. Glass RJ, Glass LM, Beyeler WE, Min HJ (2006) Targeted social distancing design for pandemic influenza. Emerg Infect Dis 12: 1671-1681. Available: http://www.cdc.gov/ncidod/EID/vol12no11/06-0255.htm Accessed 11 April 2008.

14. Glass RJ, Min J, Beyeler WE, Glass LM (2007) Design of community containment for pandemic influenza with Loki-Infect. SAND2007-1184P. National Infrastructure & Analysis Center, Sandia National Laboratories. Available: http://www.sandia.gov/nisac/docs/MatrixReport/RJGMatrixReport.doc Accessed 11 April 2008.

15. Davey VJ, Glass RJ (2008) Rescinding community mitigation strategies in an influenza pandemic. Emerg Infect Dis 14: 365-372. Available: http://www.cdc.gov/eid/content/14/3/365.htm Accessed 11 April 2008.

16. Watts DJ, Strogatz SH (1998) Collective dynamics of 'small-world' networks. Nature 393: 440-442.

17. Newman MEJ, Park J (2003) Why social networks are different from other types of networks. Phys Rev E Stat Nonlin Soft Matter Phys 68 (3 Pt 2): 036122.

18. Palla G, Derenyi I, Farkas I, Vicsek T (2005) Uncovering the overlapping community structure of complex networks in nature and society. Nature 435: 814-818.

19. U.S. Census Bureau (2005) United States Census 2000. Available: http://www.census.gov/main/www/cen2000.html Accessed 11 April 2008.

20. Ferguson NM, Cummings DA, Cauchemez S, Fraser C, Riley S, et al. (2005) Strategies for containing an emerging influenza pandemic in Southeast Asia. Nature 437: 209-214.

21. Ferguson NM, Cummings DA, Fraser C, Cajka JC, Cooley PC, et al. (2006) Strategies for mitigating an influenza pandemic. Nature 442: 448-452.

22. Germann TC, Kadau K, Longini IM, Jr., Macken CA (2006) Mitigation strategies for pandemic influenza in the United States. Proc Natl Acad Sci U S A 103: 5935-5940.

23. Longini IM, Jr., Nizam A, Xu S, Ungchusak K, Hanshaoworakul W, et al. (2005) Containing pandemic influenza at the source. Science 309: 1083-1087.

24. Moser MR, Bender TR, Margolis HS, Noble GR, Kendal AP, et al. (1979) An outbreak of influenza aboard a commercial airliner. Am J Epidemiol 110: 1-6.

25. Hayden FG, Fritz R, Lobo MC, Alvord W, Strober W, et al. (1998) Local and systemic cytokine responses during experimental human influenza A virus infection: relation to symptom formation and host defense. J Clin Invest 101: 643-649.

26. Writing Committee of the Second World Health Organization Consultation on Clinical Aspects of Human Infection with Avian Influenza (H5N1) Virus (2008) Update on avian influenza A (H5N1) virus infection in humans. New Engl J Med 358: 261-273.

27. Leekha S, Zitterkopf NL, Espy MJ, Smith TF, Thompson RL, et al. (2007) Duration of influenza A virus shedding in hospitalized patients and implications for infection control. Infect Control Hosp Epidemiol 28: 1071-1076.

28. Cauchemez S, Carrat F, Viboud C, Valleron AJ, Boelle PY (2004) A Bayesian MCMC approach to study transmission of influenza: application to household longitudinal data. Stat Med 23: 3469-3487.

29. Glass LM, Glass RJ (2008) Social contact networks for the spread of pandemic influenza in children and teenagers. BMC Public Health, 8:61. Available: http://www.biomedcentral.com/1471-2458/8/61 Assessed 11 April 2008.

30. Diekmann O, Heesterbeek JAP (2000) Mathematical epidemiology of infectious diseases. Chichester, West Sussex, England: Wiley.

31. Longini Jr. IM, Koopman JS, Haber M, Cotsonis GA (1988) Statistical inference for infectious diseases. Risk-specific household and community transmission parameters. Am J Epidemiol 128: 845-859.

32. Mills CE, Robins JM, Lipsitch M (2004) Transmissibility of 1918 pandemic influenza. Nature 432: 904-906.

33. Centers for Disease Control and Prevention (2007) Infection control guidance for the prevention and control of influenza in acute care facilities. Available: http://www.cdc.gov/flu/professionals/infectioncontrol/pdf/acutecare.pdf Accessed 11 April 2008.
